# Dynamic balance of a bipedal robot using neural network training with simulated annealing

**DOI:** 10.3389/fnbot.2022.934109

**Published:** 2022-07-28

**Authors:** Yoqsan Angeles-García, Hiram Calvo, Humberto Sossa, Álvaro Anzueto-Ríos

**Affiliations:** ^1^Computational Cognitive Sciences Laboratory, Center for Computing Research, Instituto Politécnico Nacional, Mexico City, Mexico; ^2^Robotics Laboratory, Center for Computing Research, Instituto Politécnico Nacional, Mexico City, Mexico; ^3^Unidad Profesional Interdisciplinaria en Ingeniería y Tecnologías Avanzadas, Instituto Politécnico Nacional, Mexico City, Mexico

**Keywords:** simulated annealing, bipedal robot, neural network control, neurorobotics, machine learning

## Abstract

This work proposes using an evolutionary optimization method known as simulated annealing to train artificial neural networks. These neural networks are used to control posture stabilization of a humanoid robot in a simulation. A total of eight multilayer perceptron neural networks are used. Although the control is used mainly for posture stabilization and not displacement, we propose a posture set to achieve this, including right leg lift in sagittal plane and right leg lift in frontal plane. At the beginning, tests are carried out only considering gravitational force and reaction force between the floor and the humanoid; then tests are carried out with two disturbances: tilted ground and adding a mass to the humanoid. We found that using simulated annealing the robot maintains its stability at all times, decreasing the number of epochs needed to converge, and also, showing flexibility and adaptability to disturbances. The way neural networks learn is analyzed; videos of the movements made, and the model for further experimentation are provided.

## 1. Introduction

Since ancient times, machines have been created that attempt to replicate the human form (Boden, 2006). With the development of robotics, this search for the development of machines with human characteristics has continued. This search is of vital importance as we aim for robots that can carry out tasks that at the moment are achievable only by human beings. A very useful feature that human morphology possesses is the ability to locomote and this feature is the focus of this work. Research has been done that shows that human morphology is the best option when using legged robots due to its energy efficiency (Kuo A., 2007).

To program the locomotion of a biped, classical control strategies were previously used, making use of complex equations to model the dynamics of the robot, however, with the development of some areas in artificial intelligence, and especially with the rise of artificial neural networks, significant progress has been made in simplifying the locomotion control process of bipedal robots. In Jha et al. (2005), a method combining fuzzy control and genetic algorithms was proposed to control a stair-climbing biped in a simulation. Another work in which fuzzy logic was also used is in Murakami et al. (1995) where a fuzzy controller was used for each leg in a biped. In Miller (1994) and Kim et al. (2005, 2012) the authors use trained neural networks with supervised learning to control the balance of a biped. In Lin et al. (2006) and Wu et al. (2007) unsupervised learning is used to control the biped, however these proposals have the disadvantage of additionally needing a controller to compensate the torque with a PID controller. In other applications, Sun et al. (2021) uses a neural network-based adaptive control approach to stabilize the airgap of the nonlinear maglev vehicle.

In this work, what is sought is to use a posture stability control method for a biped in a simulated environment. This control is based on artificial neural networks and evolutionary optimization, but unlike other works, the use of transfer equations and other classical control methods is ruled out. It is sought that the control system has a simpler implementation allowing the algorithm to be easily understood and reproducible while its performance meets the assigned task. As a contribution to stability control strategies in bipeds, this paper proposes a method in which only artificial neural networks trained by evolutionary optimization methods are used to achieve stabilization in the posture of a simulated biped in a computer-generated environment (MATLAB's Simulink). The novelty of this work is that it uses an optimization method in which prior knowledge is not required, since its operation is similar to reinforcement learning, being an important difference that learning is done in real time, so that fewer iterations are required.

This work focuses on the goal to achieve a stable standing position in each moment. A sequence of these positions creates movement. With the knowledge of this positions, it is possible to produce a faster motion using reinforcement learning (Gil et al., 2019), among other techniques; not only for walking on a straight line but also to make another movements including movement with disturbances. The prediction of movement can be applied changing the architecture to a recurrent neural network, this could also help to achieve a faster motion.

The benefits of having this technology are that there is no need of large datasets to train the neural network, it can be trained online while the robot is moving. It can also be implemented in other morphologies. So it can be applied in robots with different shapes and the performance should be the same. Nevertheless, more research is necessary.

The rest of the document is organized as follows: In Section 2, the state of the art is presented, works related to the area of bipeds are described. The works are presented starting with classical approaches, then works with more modern control methods are shown. Section 3 describes the methodology followed for the development of this work. Section 4 details the experiments carried out and the results obtained. Finally, in Section 5 we draw our conclusions, and some proposals are made to continue developing the project proposed here.

## 2. Related work

Bipedal locomotion is an area that has been developed for several decades and for locomotion, balance control is an indispensable requirement. Bipedal robots are high dimensional systems, the dimension varies depending on the configuration of the robot but even those systems that only have the legs and waist, have many dimensions. A common way to solve the dimensionality problem is to represent the biped as a low-dimensional inverted pendulum (Kajita et al., 2001; Kuo A. D., 2007; Pratt and Drakunov, 2007). The robot is thus controlled so that its center of mass follows a specific target. However, this approach has some challenges: finding the stable solution in the complete model, deciding how to associate the states of the pendulum with the complete high-dimensional system, even realizing the correct model of the inverted pendulum is not an easy task (Da and Grizzle, 2019). These are the reasons why a different non-linear control option is proposed. The ability of neural networks to approximate functions makes them a valuable tool for the design of nonlinear controllers (Plumer, 1996; Zhang et al., 2003; Geng et al., 2006).

Movement trajectories can be generated depending on the application. For example, that a bipedal robot moves in a straight line. However, these trajectories usually do not consider disturbances that may exist in the environment. Therefore, the adaptability of the bipedal control system to environmental disturbances is an important aspect to take into account. To solve this problem, classical control options have been considered. In Cho and Kim (2018), the authors create a dynamic model of a biped based on an inverted pendulum with a spring and damper. Later they calculated the transfer equations to make a closed control loop.

The next sections present a brief summary of some works related to the control of bipedal robots. These works focus on stabilization of bipedal posture and not on the gait cycle.

### 2.1. Dynamic posture stabilization of a biped robot SUBO-1 on slope-changing grounds

This work (Cho and Kim, 2018) describes a dynamic posture stabilization model for a bipedal robot on a tilted floor. The work uses the Zero Moment Point method to stabilize posture. It also makes use of an altering spotter that was designed to counteract alteration due to ground tilt. This work focuses on the control of *blind walk* on globally sloped terrain. Without using a vision system or any mechanism in the foot. It is assumed that the slope of the terrain changes continuously and that the floor is flat and without local slopes. Combining Zero Moment Point control and disturbance observer, a stabilization strategy using force sensors in the feet and an inertial sensor in the pelvis is proposed.

The control frequency used in that work is 200 Hz, for which the central controller receives the sensory data and sends the positions in a period of 5 ms. The structure of the walking algorithm consists of a walking pattern generation and a posture stabilizer with feedback. The pattern generator is a feedforward control and the posture stabilizer is a feedback control. Finally, the desired angle is calculated by solving the inverse kinematics for the two control inputs.

The robot was modeled as an inverted pendulum with a flexible joint consisting of a spring and a damper. Transfer equations were calculated to apply the control loop. At the end of the work it was concluded that adding an observer was very useful to deal with sloping floors. In addition to stabilization with ZMP control. It is hoped that in the future a control system including vision can be built for use in rough terrain.

### 2.2. Nearly optimal neural network stabilization of bipedal standing using genetic algorithm

In this work (Ghorbani et al., 2007), the stability control for a biped was studied. The model of the biped was simplified as an inverted pendulum with one joint. The controller consists of a general regression neural network with feedback that stabilizes the biped in a vertical position, and a PID control with feedback that maintains the pendulum in a vertical position. The neural network is also designed to minimize energy cost.

For that work, a General Regression Neural Network (GRNN) is used, which has the advantage that it is not necessary to define the number of hidden layers or the number of neurons per layer. When generating the trajectory, it is assumed that the biped moves in a sagittal plane and is simplified as an inverted pendulum with a rigid joint that is the foot.

As a first step, a closed-loop control with a GRNN was designed to move the pendulum in a region around the vertical position while minimizing the energy related to the cost function (torque). To increase stabilization, a PID control tuned by trial and error is activated to keep the biped upright. Three restrictions were considered: there is no lifting of the foot, there is no sliding, and the center of pressure is always maintained in the region of contact between the ground and the foot.

It has been reported that when standing subjects are exposed to small disturbances, they typically respond by moving in the sagittal plane and tend to keep their knees, neck, and hips straight, moving primarily at the ankle (Kuo, 1995). In conclusion, in the work it was possible to minimize energy consumption by comparing the proposed system with a previously proposed one (Yang and Wu, 2006). By comparison, the new system managed to cut energy consumption in half.

We have discussed several works that use modern control methods such as artificial neural networks or bioinspired algorithms; however, they still rely on classical techniques to ensure correct control of the biped. In Ghorbani et al. (2007), the authors use a general regression neural network with feedback for vertical stabilization of a biped, and a PID control with feedback to maintain the pendulum in a vertical position. Again, modeling the biped as an inverted pendulum, and using Lyapunov exponents to analyze the stability control. In the next section, we present our proposal, where we seek to use only neural networks for the purpose of moving a biped humanoid without losing its balance.

## 3. Methodology

Our proposed method that allows a biped humanoid to move without losing its balance consists of six stages.

1. Calculation of direct kinematics equations of the biped
2. Encoding of training algorithm
3. Modeling of the biped
4. Sense data filtering
5. Neural network training
6. Testing.

The next sections with provide more details on our proposed model.

### 3.1. Kinematic model of the biped humanoid

For this work, a kinematic model of the biped was conceived in order to calculate the center of mass and thus perform a stability analysis. Link lengths were modeled on average adult male limb measurements. The model has 20 degrees of freedom, however some of these will remain rigid during the simulation. Of the 20 degrees of freedom, 16 have a biological counterpart and four are necessary to make the complete model. The joints with a biological counterpart are: ankle, knee, hip, shoulder and elbow. Of these joints, the knee and elbow have one degree of freedom, while the ankles, hips, and shoulders have two degrees of freedom each.

In the model, both legs are modeled as a single powertrain. This indicates that the foot that is resting on the ground is the base of the robot. Therefore, the point *X*_0_*Y*_0_*Z*_0_ corresponds to the center of the foot of the supporting leg. In case both legs serve as support, *X*_0_*Y*_0_*Z*_0_ is taken from the center of the foot of the left leg.

With the kinematic model, the position equations were calculated by making the necessary multiplications of the homogeneous transformation matrices. The position equations of each joint in the X, Y, and Z axes are taken from the last column of the matrix resulting from said matrix multiplications. Once the position of each joint is known and assuming the body has a uniform mass distribution, the center of mass can be calculated with respect to the first link (which will default to the left foot unless otherwise stated). In this model there are 13 different elements, which are:

- Left foot with mass *m*_1_= 578 g
- Right foot with mass *m*_2_= 578 g
- Left tibia with mass *m*_3_= 4,000 g
- Right tibia with mass *m*_4_= 4000 g
- Left femur with mass *m*_5_= 5,000 g
- Right femur with mass *m*_6_= 5,000 g
- Hip with mass *m*_7_= 6,000 g
- Torso with mass *m*_8_= 18,000g
- Left humerus with mass *m*_9_= 3,000 g
- Right humerus with mass *m*_10_= 3,000 g
- Left radius with mass *m*_11_= 2 000 g
- Right radius with mass *m*_12_= 2,000 g
- Head with mass *m*_13_= 4,189 g

Considering that each *cm*^3^ equals 1 g. The formula to calculate the center of mass on the X axis is:


(1) $$\begin{array}{r} X=\overset{13}{\underset{i=1}{\sum }}X_{i}*m_{i} \end{array}$$

And the same procedure is followed for the Y and Z axes.

### 3.2. Using simulated annealing for training artificial neural networks

The problem we are trying to solve at this point is that we have a movement to perform (for example, raising the right leg laterally) and we have the data on the angles of each joint to achieve this movement, but the available data does not consider any stability criteria. So, if such data is entered into the simulation, it is likely that the biped will fall as it is not in a stable position. Therefore, the control system must find the value of the angle for each joint that allows the biped to remain stable. As there are no environmental disturbances, the stability criterion only considers that the center of mass of the biped is within the support polygon, which is delimited by the position of the feet of the humanoid.

The control system chosen to solve this task is a multilayer perceptron type neural network. This neural network requires unsupervised training, since, although the data at the input (the desired position) is known, there is no set of training data at the output.

For the training of neural networks in an unsupervised way, simulated annealing was used. As seen in the theoretical framework, this algorithm has shown great performance in optimization. Simulated annealing has been used in a wide variety of problems. However, depending on the problem, it can be encoded in different ways.

The first variable to define is an error function. For convenience we will call this function *E*[*f*(*s*)], where **s** is the output of the network and **f(s)** is a function that represents the environment in which the element is evaluated. output performance. If the training is not online, at each iteration the input data set can be evaluated and fed into the function f(s). Then calculate the error of each of the outputs and, as is common in backpropagation training, calculate the mean square error. With the value of the error it is evaluated if the change is accepted or not. To calculate the mean square error, Algorithm 1 is followed.

**Algorithm 1:**
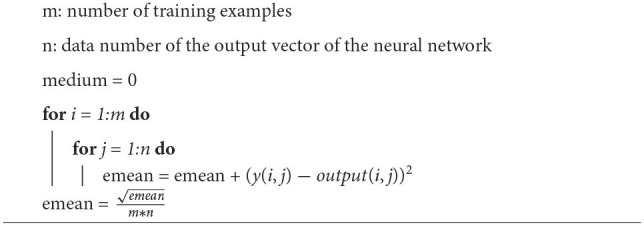
Calculation of the mean error (emean).

To use simulated annealing in neural network training, each weight and each bias are considered as a dimension of the problem. The calculation of the neighbor of a given point will be done for each of the variables of the neural network. Ideally, in each iteration, the neighbor of each one of the variables would have to be calculated and see if with the neighbor there is a better performance, however, due to the fact that in the perceptron the network is completely connected, there is a large number of variables that increase with each neuron added to each layer. This is why the algorithm is coded so that it only calculates the neighbor of K variables for each iteration. Each modified variable is chosen randomly. The training algorithm for the neural network is shown in Algorithm 2.

**Algorithm 2:**
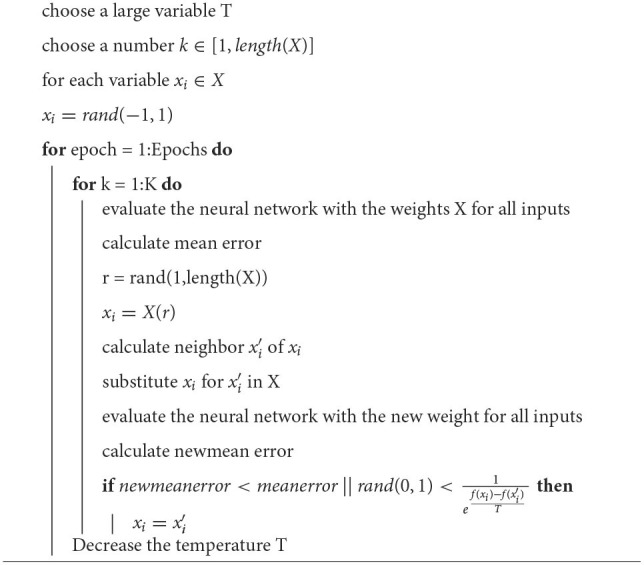
Neural network training method.

**Figure 1:**
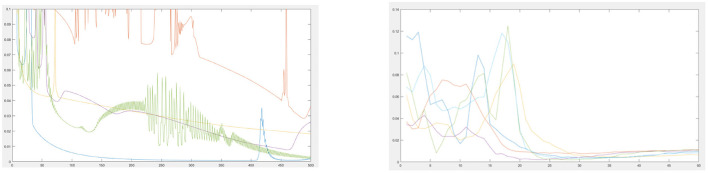
Error by epoch when training without **(left)** and with **(right)** simulated annealing.

Figure 1 shows an example of the error by epoch without (left) and with (right) simulated annealing for a sample of joint points. Without simulated annealing, the number of epochs is greater, and an unstable progression can be observed.

### 3.3. Modeling the biped

**Figure 2:**
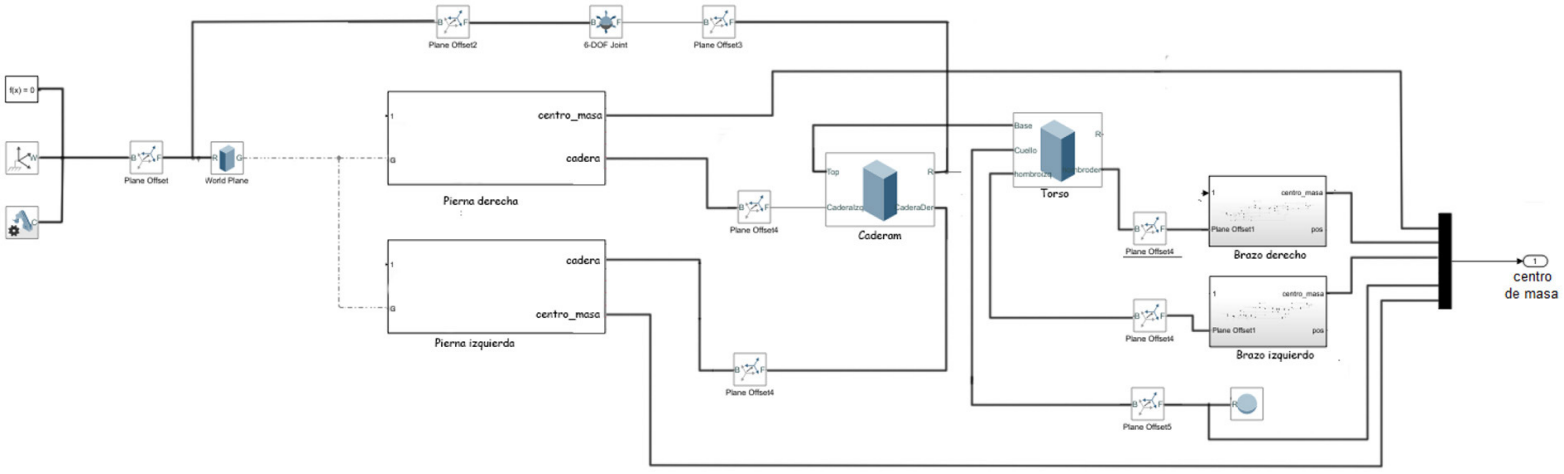
Block model of the complete system.

The biped model consists of a series of rectangular blocks joined by rotational joints modeled in Simulink. Simulink was chosen because, being part of MATLAB, it does not need to connect to external programs and all the code is written in MATLAB. Simulink also allows to obtain useful information since it has blocks of pressure sensors or inertial sensors. Additionally, the position of each block in the three axes can be known, but this last feature will not be used. For the distance of each block, the average measurements of the extremities of an adult man are taken into account. The humanoid model consists of two legs, made up of three blocks each, a torso, hips, two arms, made up of two blocks each, and a head. Figure 2 shows the total connection of the humanoid, however, the blocks of the arms and legs are kept as a subsystem to make the diagram more understandable.

In order to illustrate the posture control system applied to movement, in the following section we present details for right leg raise in frontal plane.

## 4. Experiments and results

### 4.1. First neural network training for biped balance

**Figure 3:**
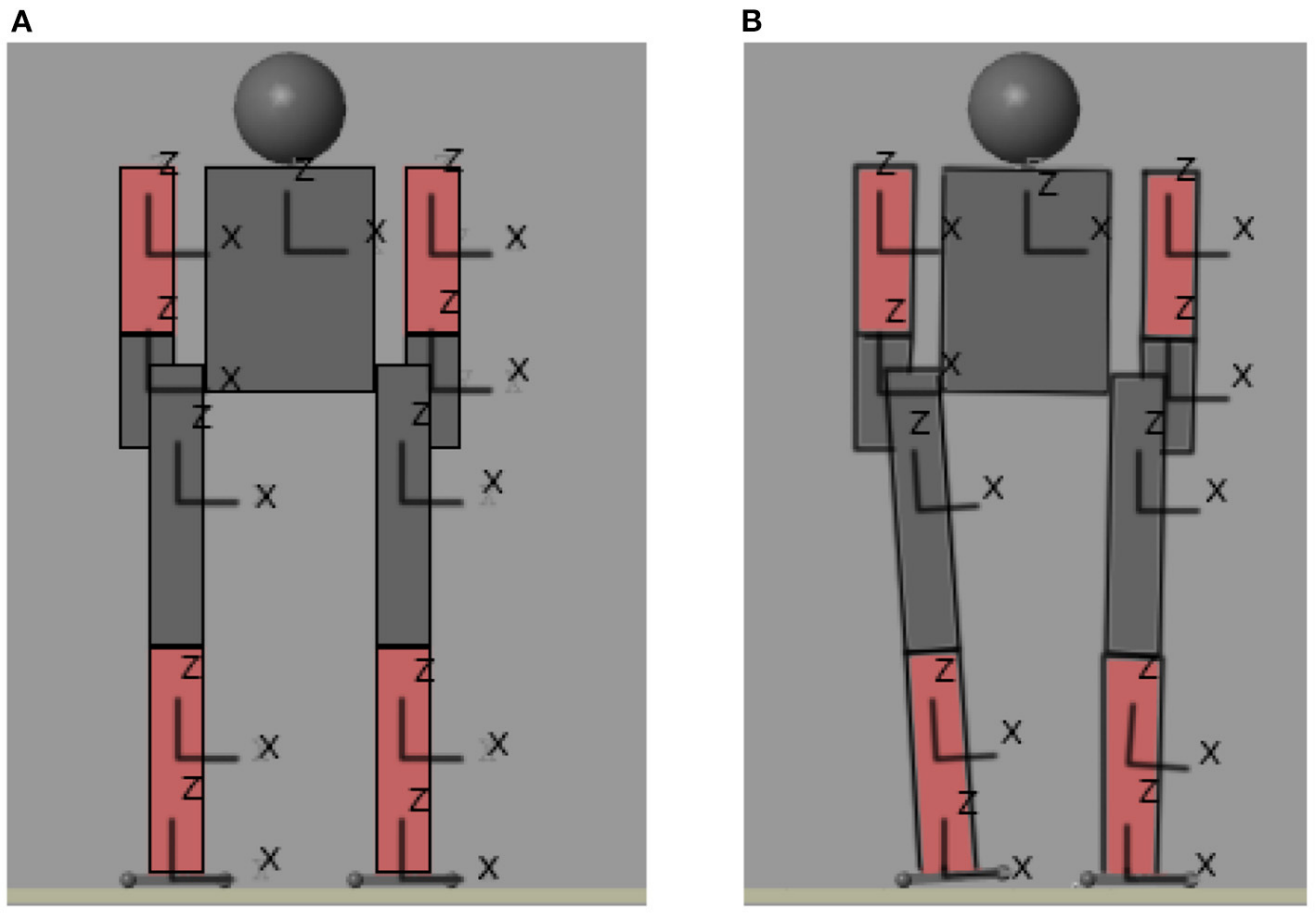
Movement of the biped. **(A)** Initial position. **(B)** First stable position.

As a first approximation for balance control of a biped, we continue with the procedure presented in the previous section. A MATLAB simulation of the biped was performed. Figure 3A shows this biped and a small movement in Figure 3B. This simulation is a plot of the position of each joint of the biped calculated in Section 3.1. In this simulation, the only information obtained to calculate the control is the center of mass. But thanks to the calculation of the Zero Moment Point, it is possible to calculate the biped's error and determine whether or not it is in a balanced position.

The purpose of this simulation is to train a neural network whose input is a series of positions of the biped to perform a movement, these positions do not consider any stability criteria, so the movement may or may not be in a stable region. In the case presented in this section, the movement consists of raising the right leg in the frontal plane. The movement consists of 50 positions in which the lateral hip joint opens from an angle 0 to π/2*rad*.^1^

**Figure 4:**
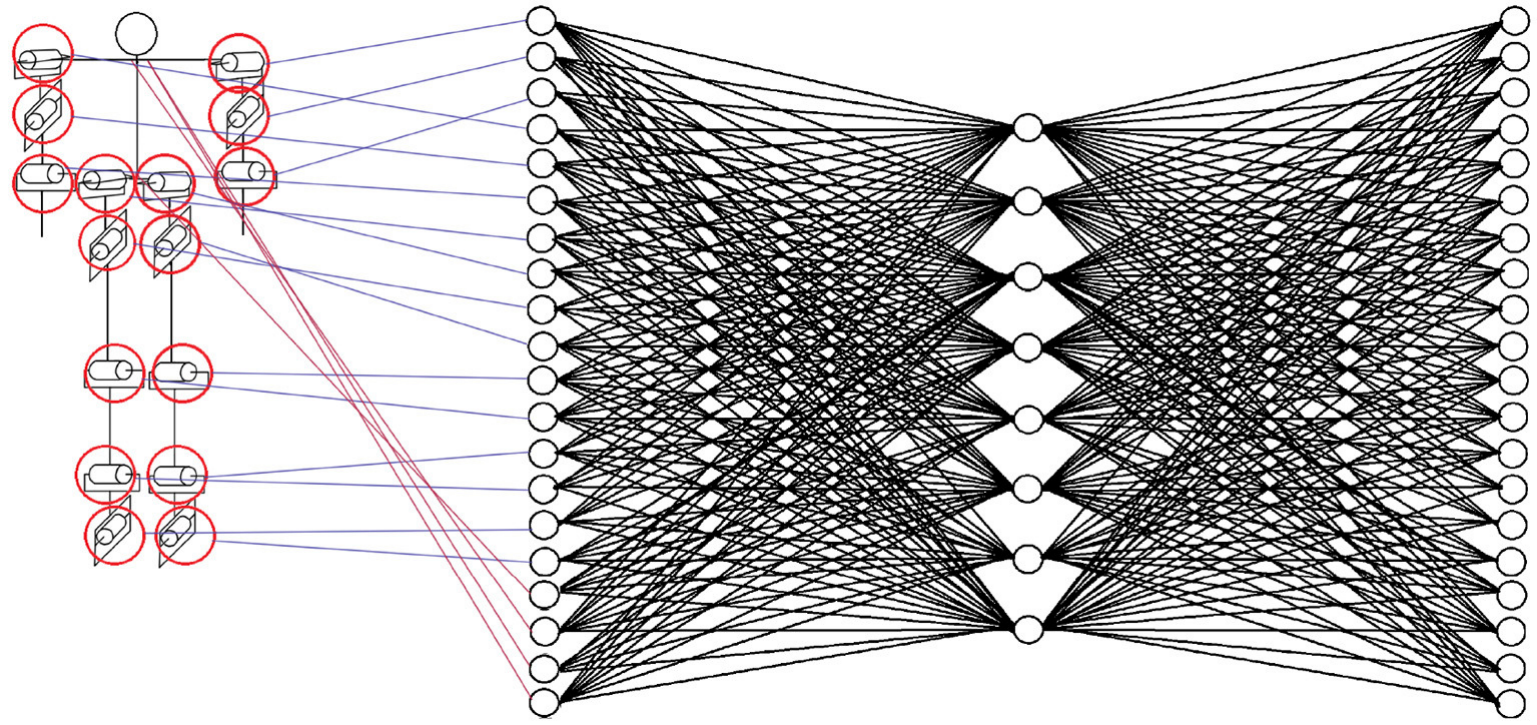
Correspondence of each joint with each neuron in the input layer.

The neural network proposed to solve this task is a fully connected multilayer perceptron-type neural network. This network has 20 neurons in the input layer and 20 neurons in the output layer. Figure 4 shows the correspondence of each joint with each neuron in the input layer. Sixteen blue lines and four red lines are shown in the image. The blue lines correspond to mobile joints that the biped has, the red lines are rigid joints that are necessary to show the complete model. The joints in red could have been omitted in the training of the neural network, but keeping them in the simulations did not affect the model. It is important to clarify that during training a limit is not considered in the angle of each joint, a limit that would exist in the case of testing with a physical robot.

Apart from the joints, there are 15 points of interest in the humanoid that are useful to calculate the similarity in the input and output trajectories of the neural network. These points coincide with the joints of the humanoid except for the points on the hands and on the head.

The problem to be solved in this neural network is the minimization of the error. The calculation of the error is made considering two metrics: the proximity of the center of mass with the 0 point of the XZ plane and the average error between the input and output joints of the network. The calculation of the mean error is done by subtracting the total of the training data set from the output data set and squaring the result. For more details on the calculation of the mean error, refer to Algorithm 1.

To calculate the center of mass error, the direct kinematics function is used, which gives the position of the center of mass in the three axes X,Y,Z. Since the humanoid stands on the XZ plane, it is on this plane that the position of the center of mass must be minimized. For this, we calculate the distance from 0 to the point of the center of mass with the equation $cm=\sqrt{x^{2}+z^{2}}$.

Both the mean error of the joints and the position of the center of mass are multiplied by a constant to give greater or lesser importance to each metric. The error related to the difference between coordinates, called ***error***_***equal***_ is multiplied by a constant α. The error related to the position of the center of mass, called ***error***_***cm***_ is multiplied by a constant β. The total error is the sum of both errors, as shown in Equation (3).


(2) $$\begin{array}{l} T_{e}\left(ep,n,m,p\right)=ep\left(4mnp+2m\right)error=\alpha *error_{equal}\\ \;+\beta *error_{cm} \end{array}$$

The parameters related to simulated annealing are: **T** s the initial temperature, **TempVar** indicates every how many epochs the temperature decreases, and **DeltaTemp** indicates the percentage of temperature that will remain after **TempVar** epochs. The variables that must be defined are:

1. T Initial temperature
2. DeltaT Temperature Change
3. TempVar, or the number of epochs before the temperature drops
4. DeltaEpoch or the number of epochs until the temperature is increased again
5. Epochs
6. LimitsW or the range in which the weights and bias are initialized
7. LimiteV or the maximum value of neighbor value for each weight
8. K or the number of weights and bias that are modified for each epoch
9. alpha and beta or the importance value given to each error parameter.

The parameters of the algorithm used in this work are shown below.

1. *T*= 1,000
2. *TempVar* = 5
3. *DeltaT* = 0.85
4. *DeltaEpoch* = 100
5. *LimitsW* = [−2, 2]
6. *LimitsV* = [−0.5, 0.5]
7. *K* = 100
8. α = 0.75
9. β = 0.25

To know if the center of mass is within the support polygon, it must not exceed 0.085 both in the x axis and in the z axis. This due to the architecture of the humanoid that is taken as a reference.^2^

### 4.2. First standing training (front plane right leg raise)

The biped must always start in position 0 (in which all joints are at 0), then take it to some initial position other than 0 (if required) and from there start doing the experiments. This is because if the robot is initialized in a different position, the pressure sensors can appear inside the ground and mark very high values that decrease over time until the biped “goes up” and is positioned on the ground. It is possible to initialize the biped to a position other than 0, however there are sometimes errors, so it is preferable to follow the instructions above. Figure 3A shows the biped in position 0, which is when all its joints are at 0 position. For this simulation, we chose to perform the movement which is to raise the right leg 90° in the frontal plane. To accomplish this, the biped was started at position 0. Once in this position, instructions are given for the biped to move each of its joints to a range of [−0.2, 0.2] radians, moving one joint at a time. This to know what is the position in which the center of mass is placed on the left leg, which will be the one that remains on the ground^3^. To know the position of the center of mass, the Inertial Sensor block of the Simscape Multibody Body Elements library was used. This block gives information on the position of the center of mass of each body. Therefore, calculating the average of the center of mass of each body, gives the total center of mass of the humanoid. This position is the absolute position of the center of mass with respect to point 0 of the world in which we are working. To calculate the position relative to the supporting leg it is necessary to subtract the total center of mass minus the center of mass of the foot of the supporting leg.

Once the degree configuration of the joints necessary to place the center of mass on the supporting leg is known, the neural networks are trained to reach said position. A total of eight neural networks were used: two for right hip (one for each joint) and other two for left hip, one for each knee and one for each arm.

**Figure 5:**
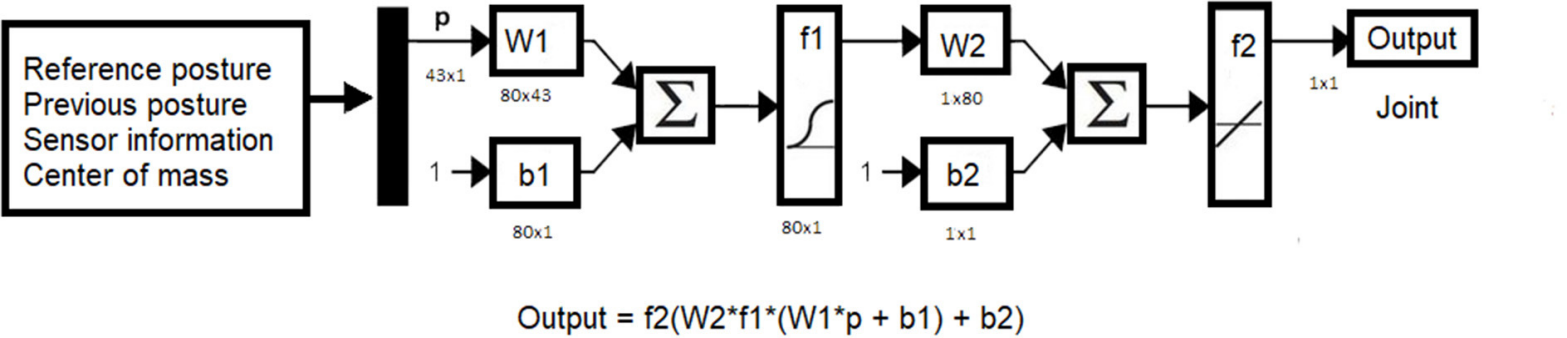
Architecture of the neural networks used.

For the control of the ankle joints, a different algorithm was used, which is explained later. The architecture is the same for all neural networks and is shown in Figure 5. The inputs of the neural network are:

1. Reference path consisting of 16 data.
2. Previous position consisting of 18 data.
3. Left and right sensors consisting of 8 data.
4. Relative position of the center of mass of the body with respect to the position of the foot of the supporting leg in the x,y plane.

**Figure 6:**
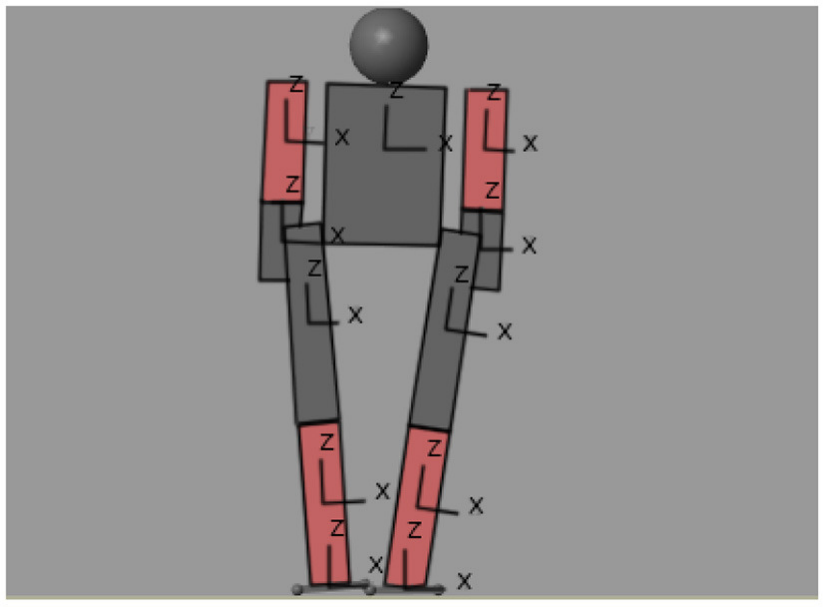
First stable position of the humanoid.

**Figure 7:**
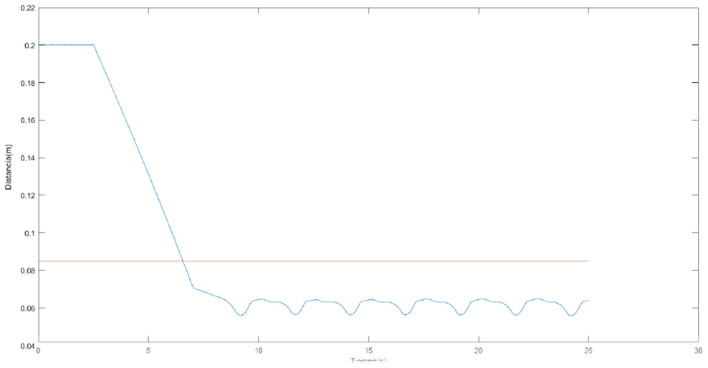
Trajectory of the center of mass on the X axis.

Hyperbolic tangent was used as activation function. The neural networks were trained so that, while at the input the position is position 0, each one delivers the necessary value to place the center of mass of the biped on the left leg, we will call this position the first position of stability, which is shown in Figure 6. Once the neural network is trained, it runs until it reaches the first stability position. Figure 7 shows the trajectory of the center of mass in the X axis of the body relative to the support leg. A red line is shown, which is the limit at which a stable position is considered if only the left leg is supported.

In this problem of optimization, we are trying to minimize the error, for this we are taking into account two parameters: the center of mass of the body and the difference between angle joint input and output. We know the center of mass of the body using the function block described called Inertia Sensor, which gives the position of the center of mass in the three axes X,Y,Z. Since the humanoid stands on the XY plane, it is on this plane that the position of the center of mass must be minimized. For this, we calculate the distance from 0 to the point of the center of mass with the equation $cm=\sqrt{x^{2}+y^{2}}$.


(3) $$\begin{array}{r} error=\alpha *error_{equal}+\beta *error_{cm} \end{array}$$

Both the error of the joint and the position of the center of mass are multiplied by a constant to give greater or lesser importance to each metric. The error related to the difference between angle joint, called ***error***_***equal***_ is multiplied by a constant α. The error related to the position of the center of mass, called ***error***_***cm***_ is multiplied by a constant β. The total error is the sum of both errors, as shown in Equation (3).

Once the biped is in the first position of stability, the first value of the position vector, which is specific for each joint, is entered into each neural network as a target. Once the difference between the network output and the target is small enough (remembering that the output may not be the same since the neural network considers the center of mass error). The target is updated to the next value in the array of positions. A brief description of the learning algorithm is shown in Algorithm 3. This ensures that it will not advance to the next position until the previous position has been learned first, and will not advance beyond the last indicated input.

**Algorithm 3:**
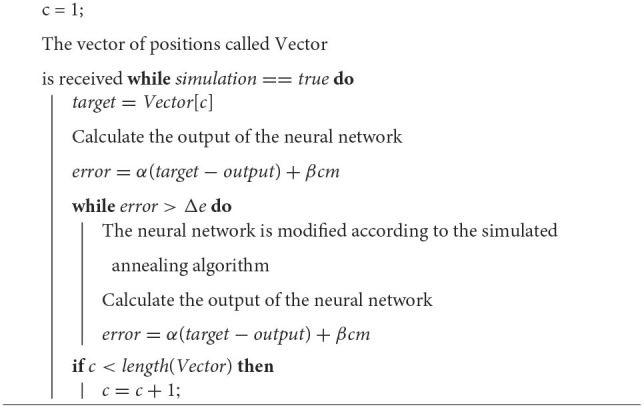
Learning algorithm in Simulink.

**Figure 8:**
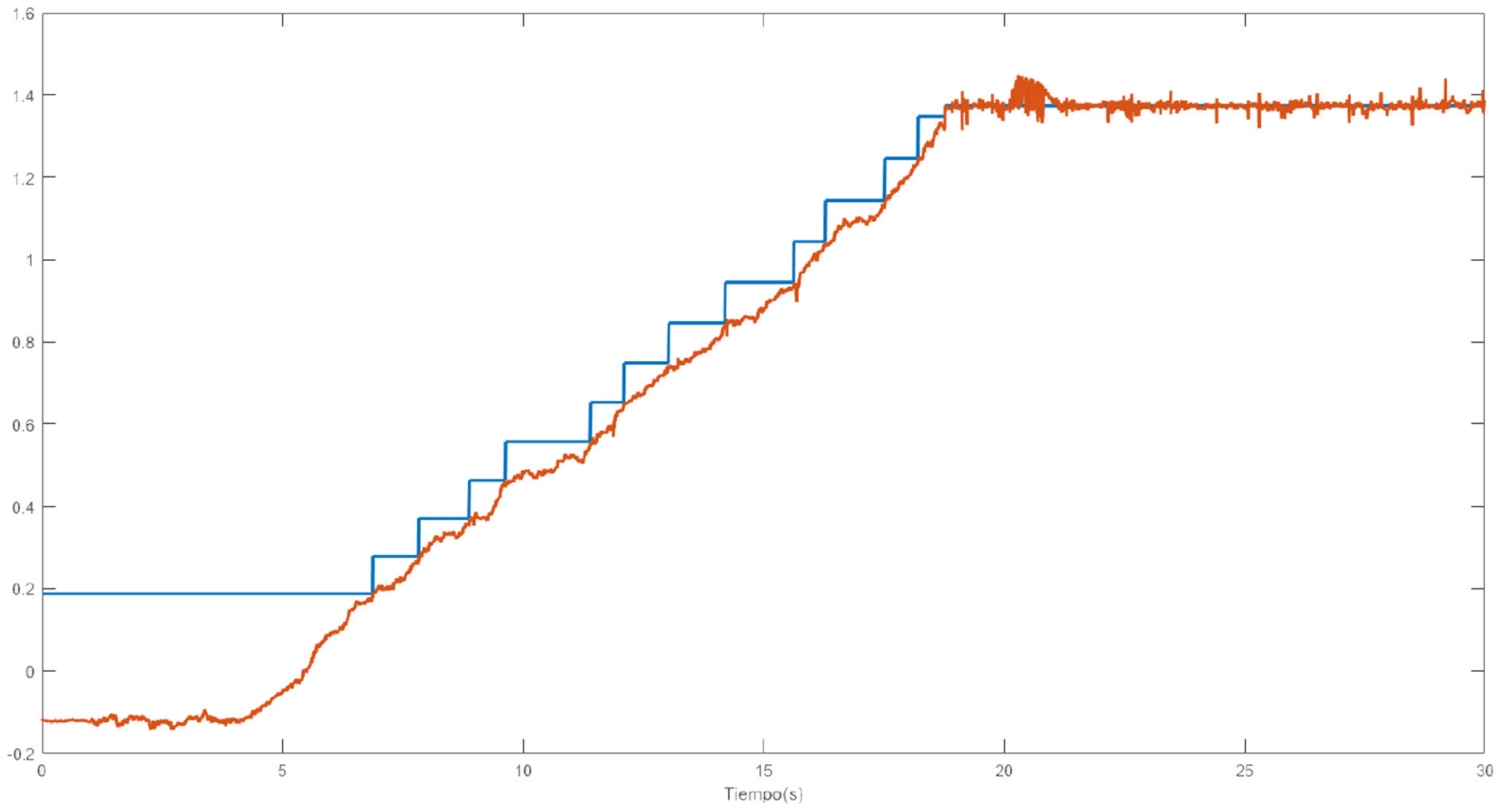
Output trajectory and target of the neural network.

Figure 8 shows how one of the neural networks learns. In this case it is the neural network that controls the lateral right hip joint. The blue signal is the target, the red signal is the output delivered by the neural network. The staggered shape of the target is due to the fact that it will not change to the next position until the error between the two signals is not small enough, that is why when the red signal approaches the blue one, the target signal jumps.

Since the neural networks ensure a stable movement for the current position, a series of inputs and their corresponding output are known to move in a stable way. So the information is stored in case you want to train a subsequent neural network in a supervised way. To store such information, a vector of inputs and outputs is initialized to zero. As the epochs progress, the inputs collected from the sensors are saved. Only those outputs that ensure a sufficiently small error are saved and not all the outputs delivered by the neural network. Algorithm 4 shows the steps to follow to collect the data that can be used later to train a neural network in a supervised way.

**Algorithm 4:** Storage method.

The output is calculated by doing the matrix multiplication. The information is sent to the model and the error is evaluated. If the error is less than or equal to a given value e, the input and output are saved in the respective vector and it is repeated from Step 1 with a new position. If the error is still not small enough, another modification is made to the neural network and steps 2–5 are repeated.

It is important to clarify that the output delivered by the neural network is not directly input to the algorithm. This is due to the sensitivity of the simulator to sudden changes in the joints.^4^ Therefore, once the neural network delivers an output with a sufficiently small error, the joints are instructed to reach this value by changing their value by Δ*c* each cycle. For this work, Δ*c* = 0.00007*rad* was selected. This value may seem small but it is necessary so that the change in position of the joint is not made abruptly.

It should be sought that the four sensors of each foot are in contact with the ground and that the force is evenly distributed in each sensor. This is why the algorithm is relatively simple. For the four joints, an algorithm similar to the one shown in Algorithm 5 is followed.

**Algorithm 5:** Ankle control algorithm.

**Figure 9:**
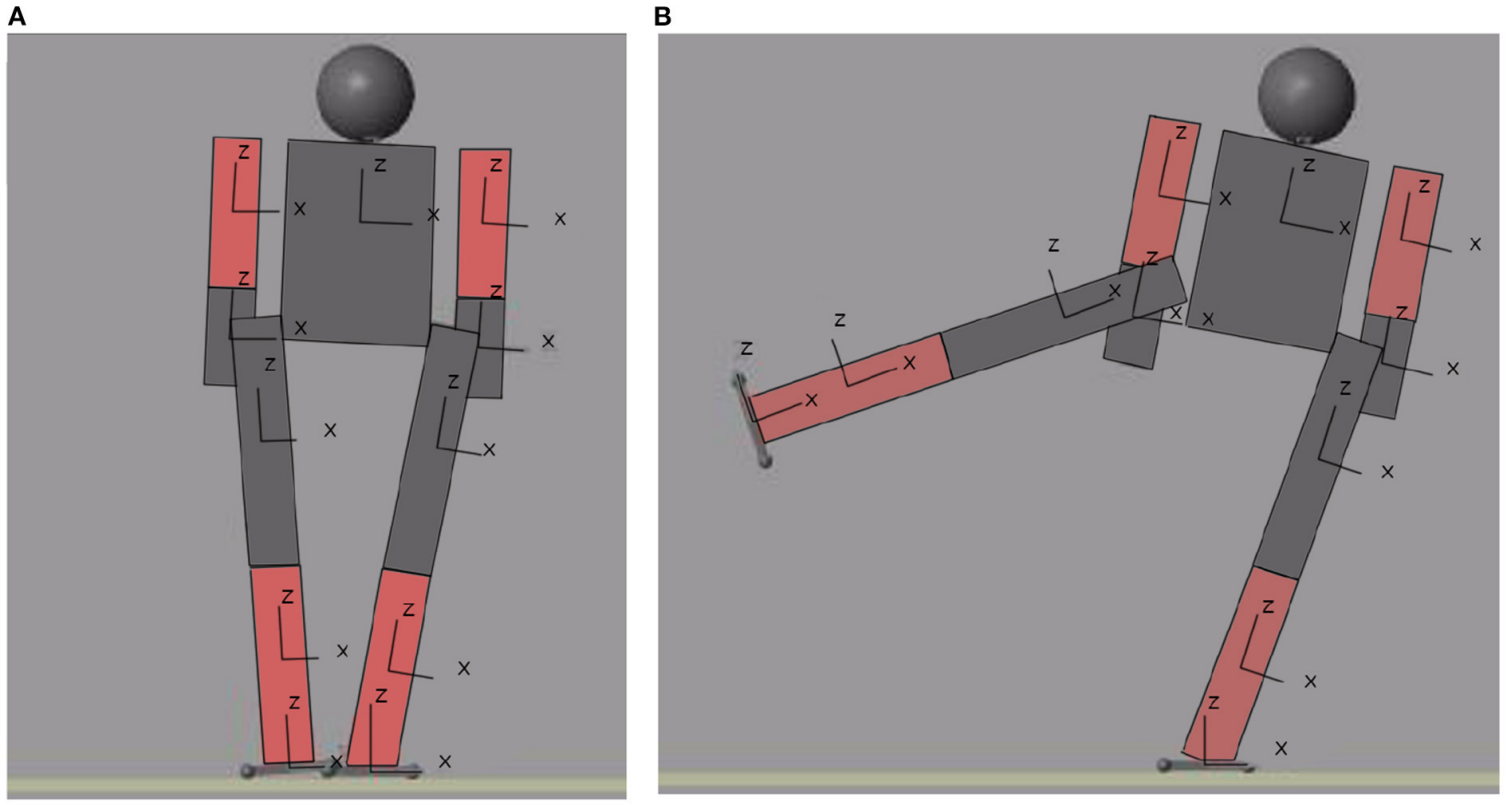
Initial **(A)** and final **(B)** position of the movement performed.

**Figure 10:**
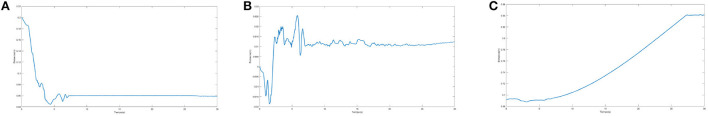
**(A)** Trajectory of the center of mass in X axis, **(B)** Y axis, and **(C)** Z axes of movement 1.

**if** *s*1 < *s*2|*s*4 < *s*3 **then**
| joint = joint + num;
**if** *s*1 > *s*2|*s*4 > *s*3 **then**
| joint = joint - num;

Figure 9 shows the initial and final position of the biped performing the movement of raising a the right leg in frontal plane^5^. The trajectory of the center of mass in the X axis is observed in Figure 10A. Recall that the biped is standing on the X,Y plane, the X axis is the frontal plane of the biped and the Y axis is the sagittal plane. Therefore, for this movement the axis that has more importance is the X axis. Figures 10B,C also shows the trajectory of the center of mass in the Y and Z axes^6^.

**Figure 11:**
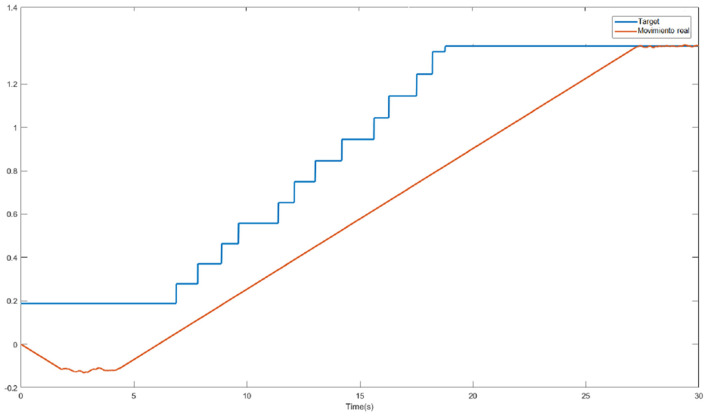
Comparison of desired move (blue) with performed movement (red).

Figure 11 shows the output of the neural network in blue as well as the trajectory of the joint that moves the right hip laterally in red. There is a clear time lag between the output of the neural network and the articulation. This displacement is due to what was previously mentioned, by having to slowly modify the angle of the articulations, the neural network converges faster than the joint reaches the desired position.

### 4.3. Training the biped with added perturbation (ground tilt)

As explained in previous sections, the training used for neural networks is a learning style that does not require full knowledge of the desired output. This **flexibility** in learning allows the neural network to learn in an environment with certain disturbances. As an added disturbance, the platform on which the humanoid is standing tilted. The tilt was 1° and was done on the X axis.

**Figure 12:**
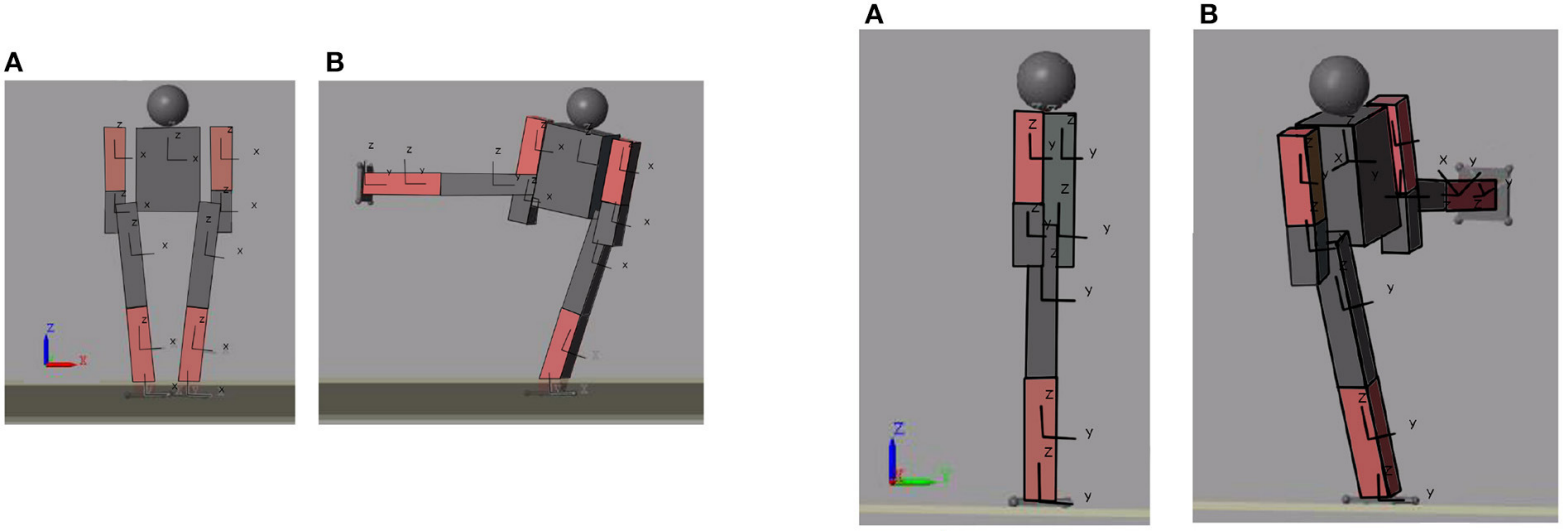
Initial and final position of the biped seen in the XZ (left–**A,B**) and YZ (right–**A,B**) planes for ground tilt.

**Figure 13:**
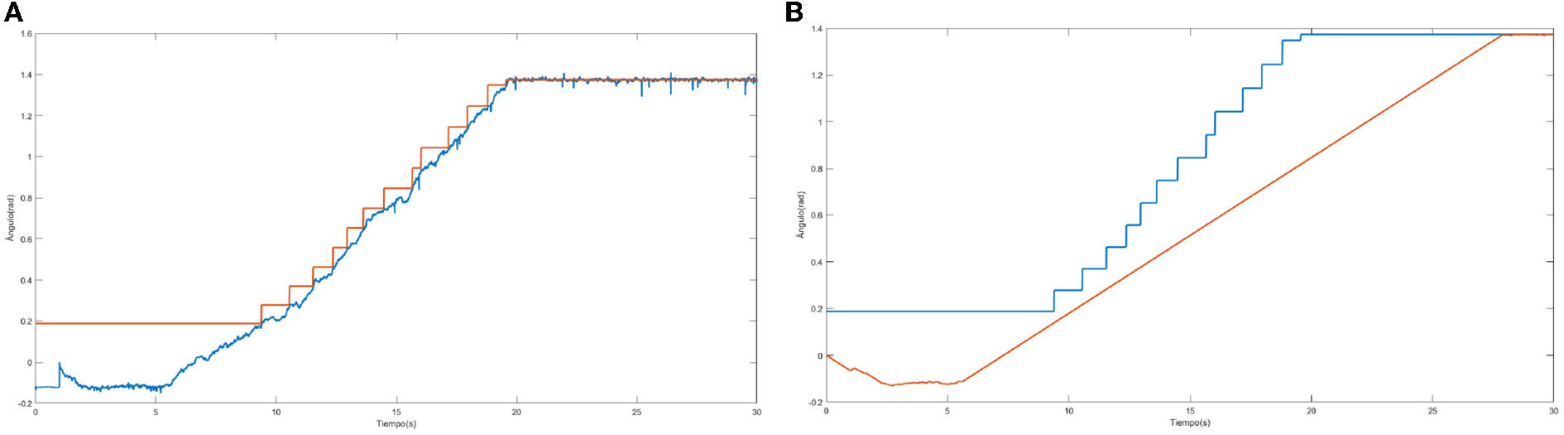
**(A,B)** Learning path for ground tilt and desired and actual motion path for ground tilt.

Figure 12 shows the initial and final position of the biped seen from the front (left) and the same positions seen from the side for a better visualization of the slope of the ground (right).^7^ The learning trajectory and the actual motion path, compared with regard to the desired one, can be seen in Figure 13. This figure shows the output trajectory of the neural network in blue, this is to visualize how the trajectory of the neural network is modified according to the desired target. Figure 13B shows the same target, this time in blue but with the actual output of the joint. In this figure, the disturbance of inclined ground is considered.

### 4.4. Training the biped with added mass perturbation

**Figure 14:**
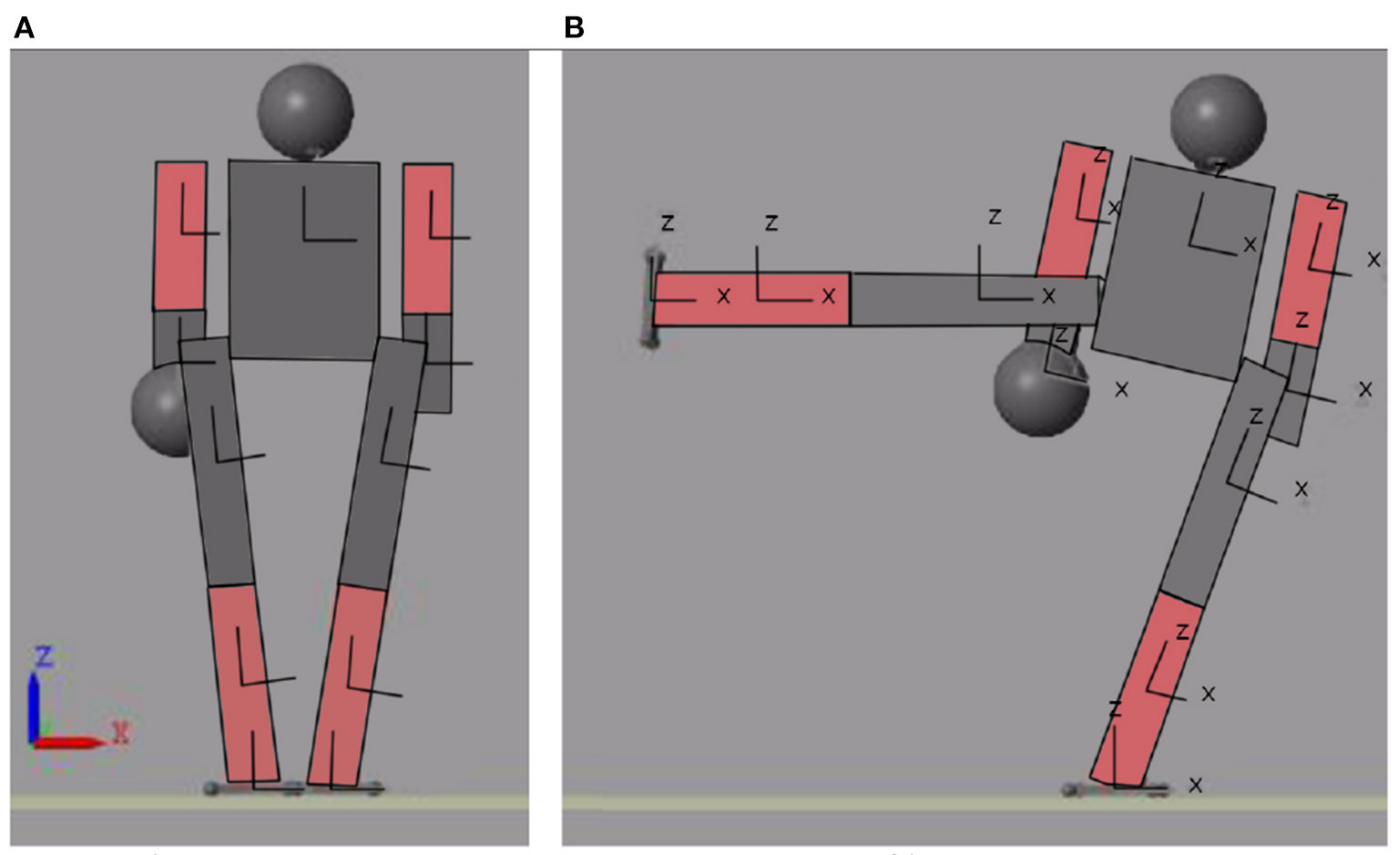
Initial and final position of the movement seen from the XZ plane. **(A)** Initial position of motion. **(B)** Final position of motion.

**Figure 15:**
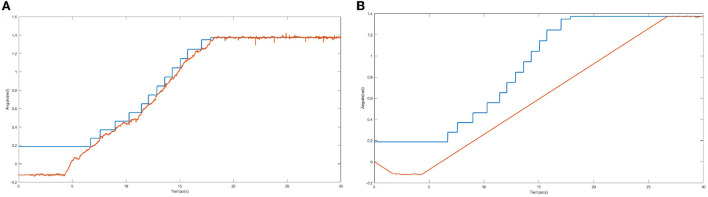
Learning Path for Added Mass and Trajectory of the desired (blue) and actual (red) movement for Added Mass **(B)**. **(A)** Desired trajectory (blue) and output network (red).

As an added disturbance a 0.1 m sphere was added to the biped's right arm. This represents 7.5% of the humanoid's weight. Figure 14 shows the initial (Figure 14A) and final (Figure 14B) position of the biped's movement. The movement he performs is the lifting of the right leg in the frontal plane. The learning trajectory and the trajectory of the desired (blue) and actual (red) movement are shown in Figure 15. This figure allows to visualize how the trajectory of the neural network is modified according to the desired target. Figure 15B shows the same target in blue, with the actual output of the joint when a mass is added to the biped.

## 5. Conclusions

This work has presented the use of neural network control for robot stabilization and displacement. Although there are already several works that use neural networks in control, they usually require the help of external control methods or complicated training of many iterations to solve the assigned task. In this case the control was implemented in a simple way with little prior knowledge. The method we have presented allows having both flexibility and adaptability, as it has been presented in the last two sections above: the neural network adapted its weights despite having changing conditions such as a floor inclination, or adding a mass. The presented method is a mixture of supervised and reinforcement learning, combined in a novel way, allowing to implement several neural network applications easily and with little prior knowledge. Simulated annealing was used as a means of training the neural networks; although this optimization method has been used previously, it is not common to apply this method for training this kind of application. We have found that using this method in the application of robot control can help to reach a goal position while keeping the biped humanoid stable. Although the presented solution is slower at some steps than other better known methods, by using simulated annealing, in overall there is a noticeable reduction in the epochs needed to converge, making this method feasible for many applications. A difficulty to consider is that the learning time is variable because, being a heuristic method, the random component in learning means that the learning time is not always optimal.

One of the applications of postural stability is that, by knowing or calculating the stable positions, it is possible to implement passive locomotion. Although this type of locomotion is more inefficient than active locomotion, it allows movement and has a robust response to disturbances. Although there is software specialized in robot simulation, sometimes it is difficult to know how to run or control these simulators. If it is not necessary to have many sensors, and the purpose is to test robots whose constitution is relatively simple, MATLAB is a good option, since within this program the necessary actuators can be controlled without the need for an external program. There is a drawback, however, in that the simulation time can be long. In this work, a humanoid was developed that can be used for future projects. The link to download the humanoid can be found at Yoqsan (2022).

### 5.1. Future work

This work presented a robot control method that had not been presented before, so it is still in its infancy and can be optimized in many areas. The simulated annealing method can be mixed with other optimization methods to find more optimal neighbors than looking for close neighbors, which is what was done in this work. Other types of architectures can also be tested, in this work a multilayer perceptron was used, however, there are other neural architectures that may help improve the response.

The objective of this work was to achieve a balance in the posture of a humanoid and, although the objective was achieved, slow movements were required to achieve it. If, in addition to the anterior position of the joint, information on the speed and acceleration of the joints is included, it would be possible to increase the speed at which the biped moves. If the error also considers a criterion of energy expenditure, it would also be possible to make the optimal movements energetically, just as human beings do.

Furthermore, having this type of locomotion, it is possible to optimize it to achieve dynamic locomotion. Experiments of this latter point are left as future work. According to the data obtained from the experiments, it is known that this method allows a robot to function in an environment where there are unknown disturbances. Motion prediction is not done yet, however, it is possible to implement another neural network or modify existing ones by adding memory and recursion to predict motion.

As seen in the experiments presented, neural networks behaved correctly despite adding disturbances, so it is possible that the same control system works to move robots with another configuration. Although this was not tested, in future works the control system could be implemented with different configurations of robots and obtain information on the difference between their movements.

## Data availability statement

The original contributions presented in the study are included in the article/Supplementary material, specifically Yoqsan (2022). Humanoid in Simulink. Further inquiries can be directed to the corresponding author/s.

## Author contributions

HC and HS: original concept and supervision. YA-G: proposal, implementation, and original draft. HC: final writing and funding. ÁA-R: guidance, supervision, and general revision. All authors contributed to the article and approved the submitted version.

## Funding

This work was partially supported by CONACyT, Mexico, and by Secretaría de Investigación y Posgrado of the Instituto Politécnico Nacional, Mexico, under Grant 20220553.

## Conflict of interest

The authors declare that the research was conducted in the absence of any commercial or financial relationships that could be construed as a potential conflict of interest.

## Publisher's note

All claims expressed in this article are solely those of the authors and do not necessarily represent those of their affiliated organizations, or those of the publisher, the editors and the reviewers. Any product that may be evaluated in this article, or claim that may be made by its manufacturer, is not guaranteed or endorsed by the publisher.

## Footnotes

^1^The movement can be seen on https://youtu.be/DKR9xuieMFk. The path marked in red is the center of mass and how it changes position according to the movement made.

^2^The video of the complete movement can be seen at https://youtu.be/ViPiFOuDbDA

^3^The video showing these movements can be found at https://youtu.be/AHMae7Ki1Go

^4^Video at https://youtu.be/HU5L8lLqmrA shows a simulation in which the ankle joint was changed from position 0 to 0.1.

^5^The full video of this move is at https://youtu.be/2SNglSgEwzw

^6^For a better visualization the video of the trajectory of the center of mass in the X and Y axes while the movement is executed can be found at https://youtu.be/xxBiI43VpeM

^7^The full motion video can be found at https://youtu.be/7ktSoxzQo_0
