# Supplementary figures and images for: Dynamic balance of a bipedal robot using neural network training with simulated annealing
Source: Front Neurorobot. 2022 Jul 28;16:934109. doi: 10.3389/fnbot.2022.934109 (PMC9366121; doi:10.3389/fnbot.2022.934109)

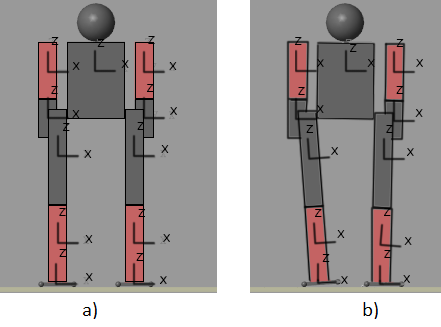

Supplement: Supplementary file 1 [file Data_Sheet_1.ZIP › figures/Pos1.png]

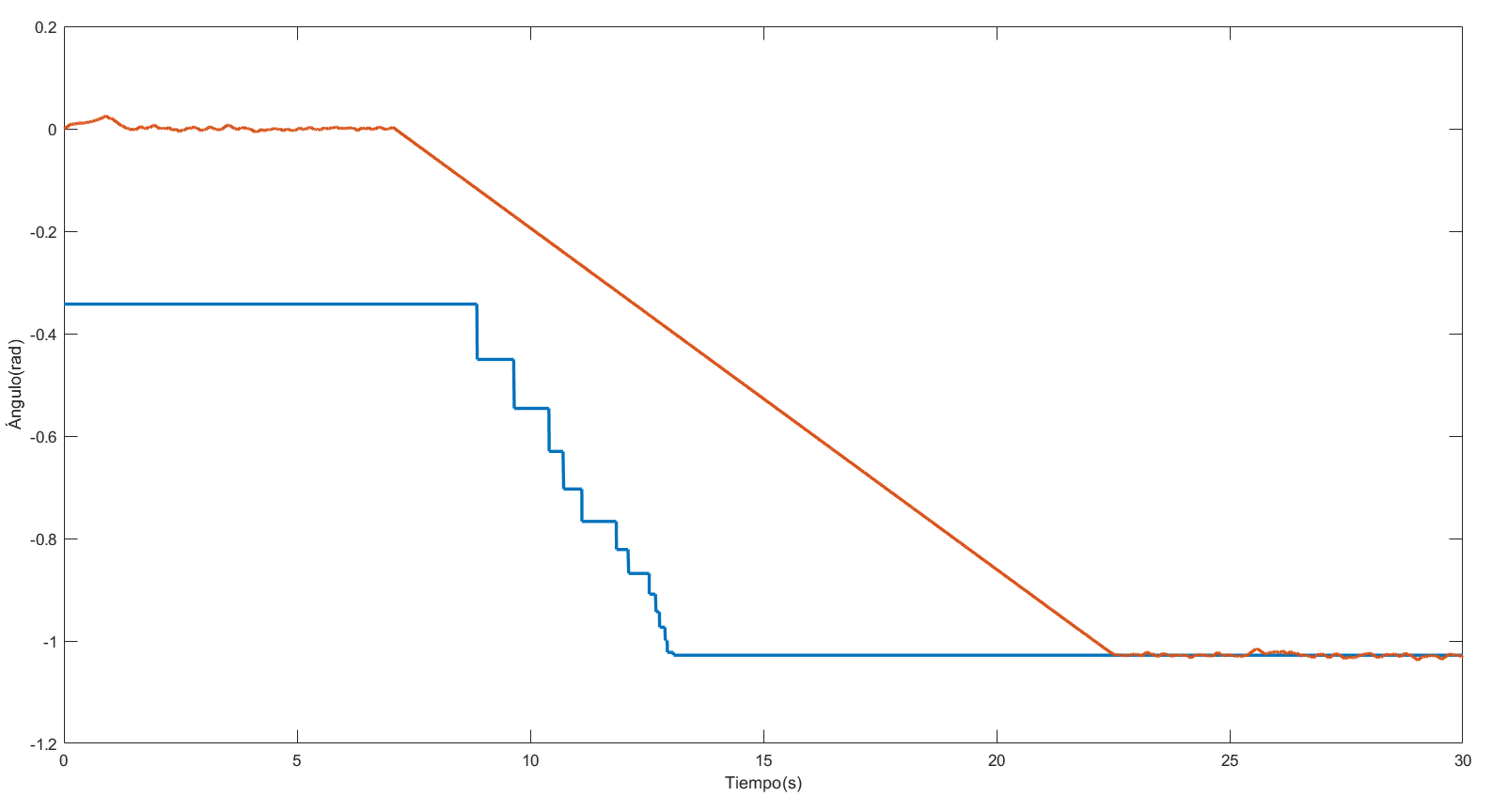

Supplement: Supplementary file 1 [file Data_Sheet_1.ZIP › figures/MovementPath5.png]

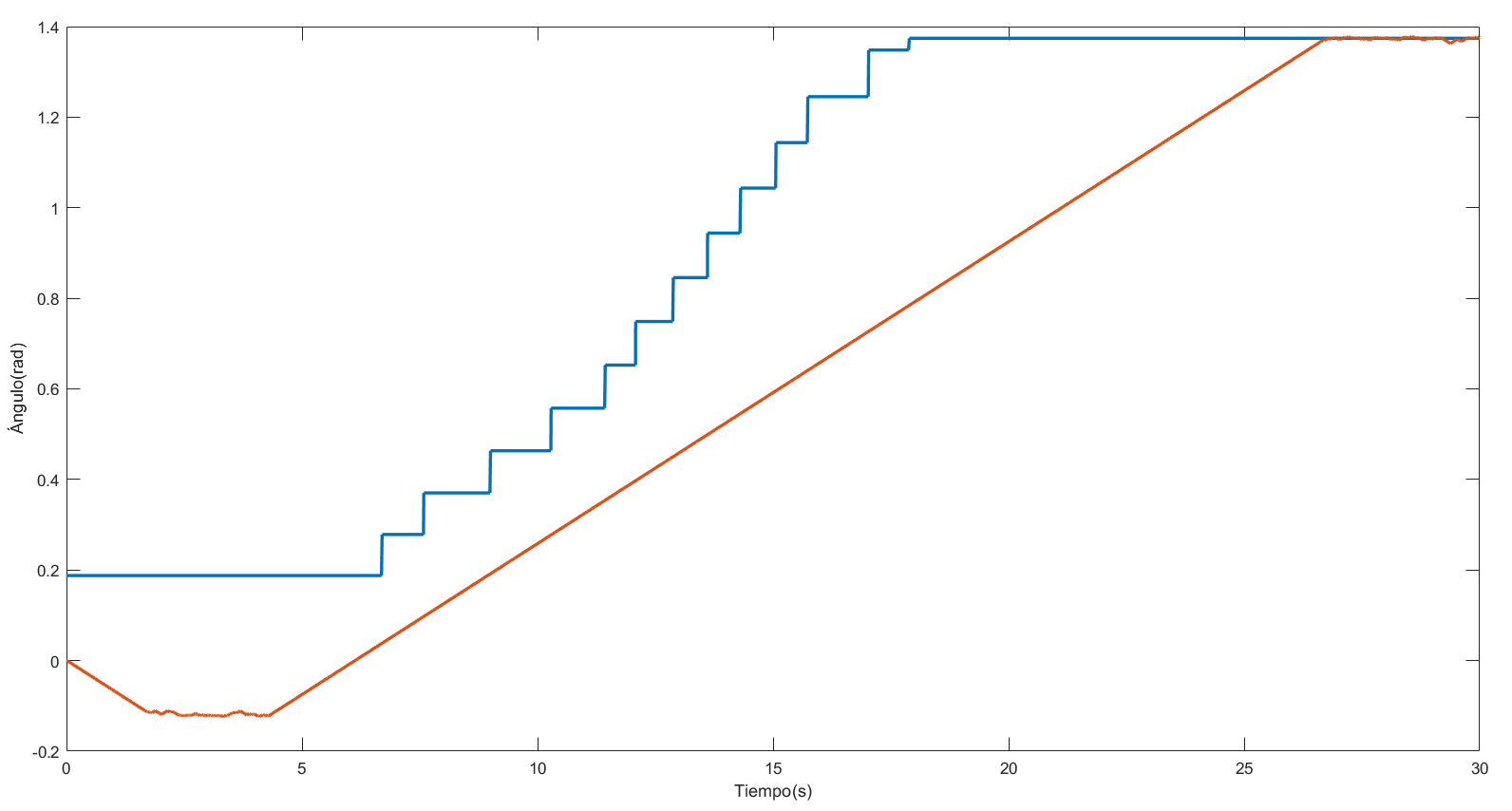

Supplement: Supplementary file 1 [file Data_Sheet_1.ZIP › figures/MovementPath4.png]

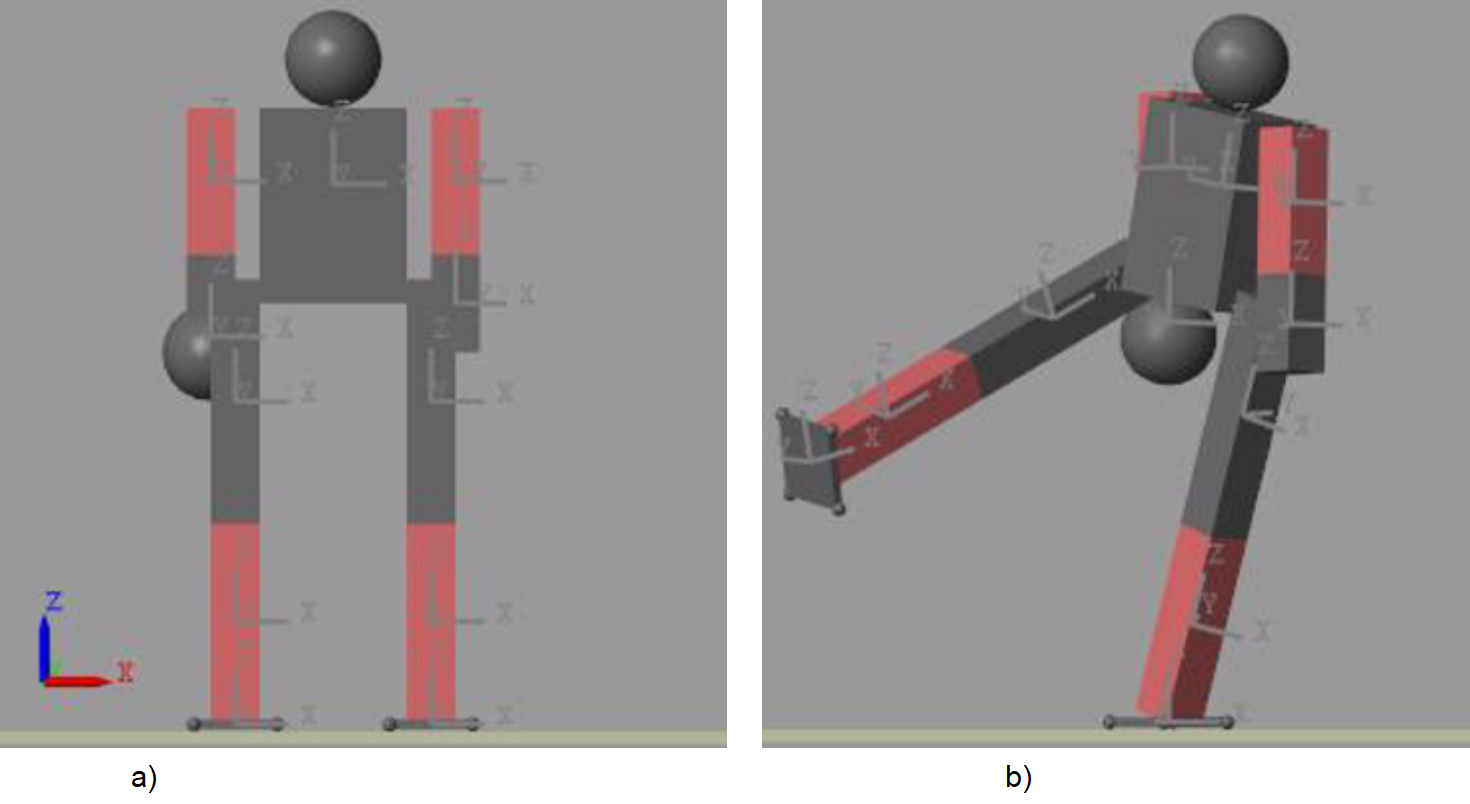

Supplement: Supplementary file 1 [file Data_Sheet_1.ZIP › figures/Move6.png]

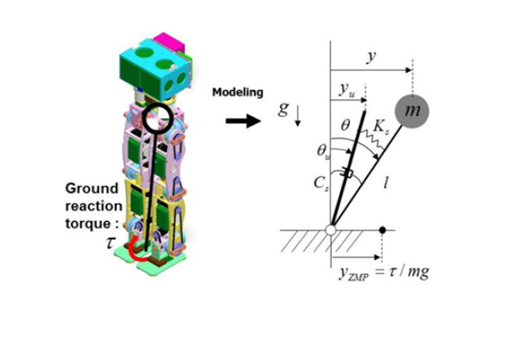

Supplement: Supplementary file 1 [file Data_Sheet_1.ZIP › figures/SimplifiedDynamicModel.png]

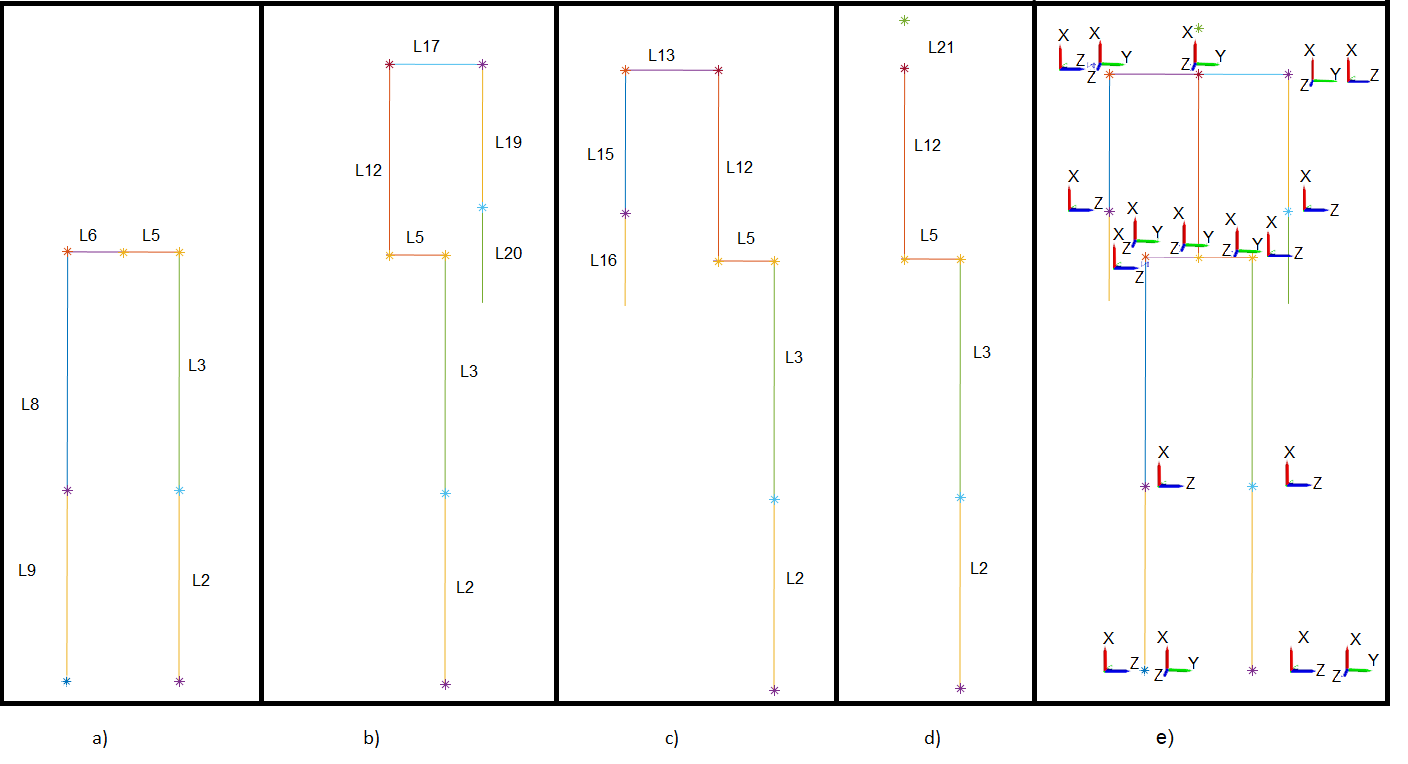

Supplement: Supplementary file 1 [file Data_Sheet_1.ZIP › figures/CD1.png]

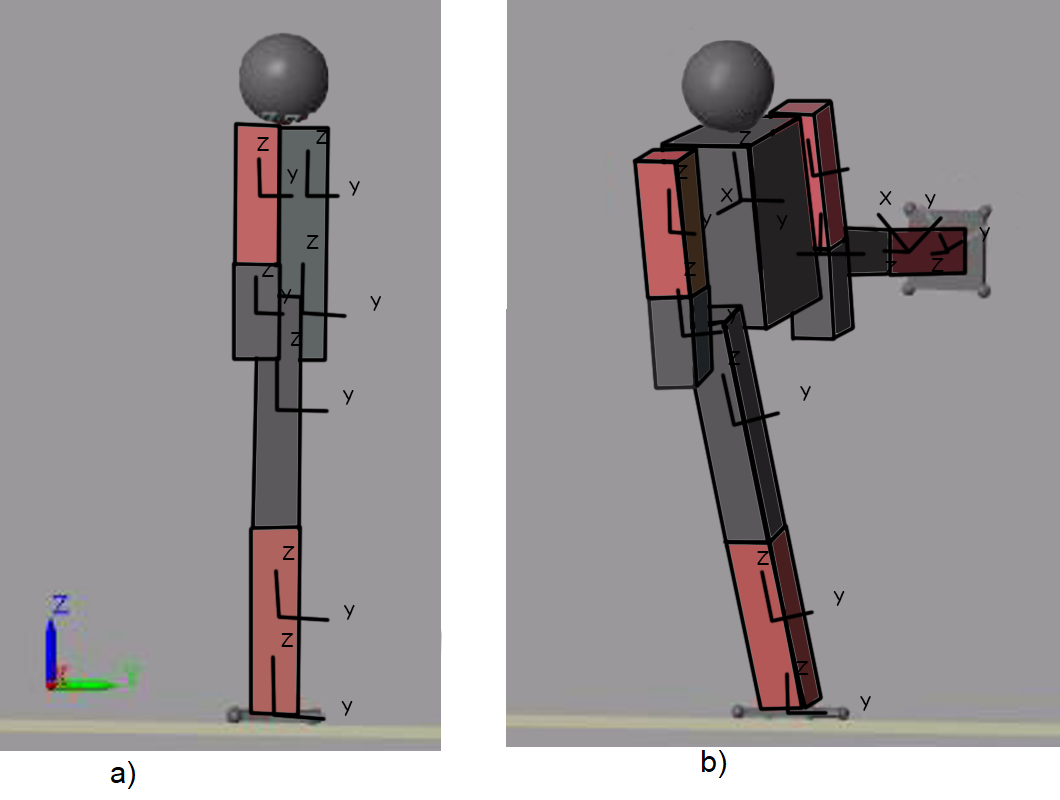

Supplement: Supplementary file 1 [file Data_Sheet_1.ZIP › figures/Move4.png]

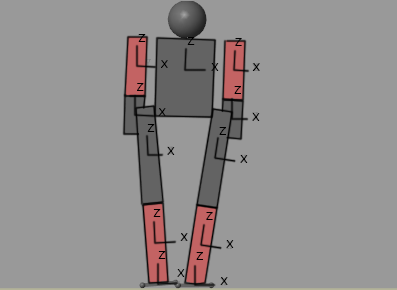

Supplement: Supplementary file 1 [file Data_Sheet_1.ZIP › figures/PosStable1.png]

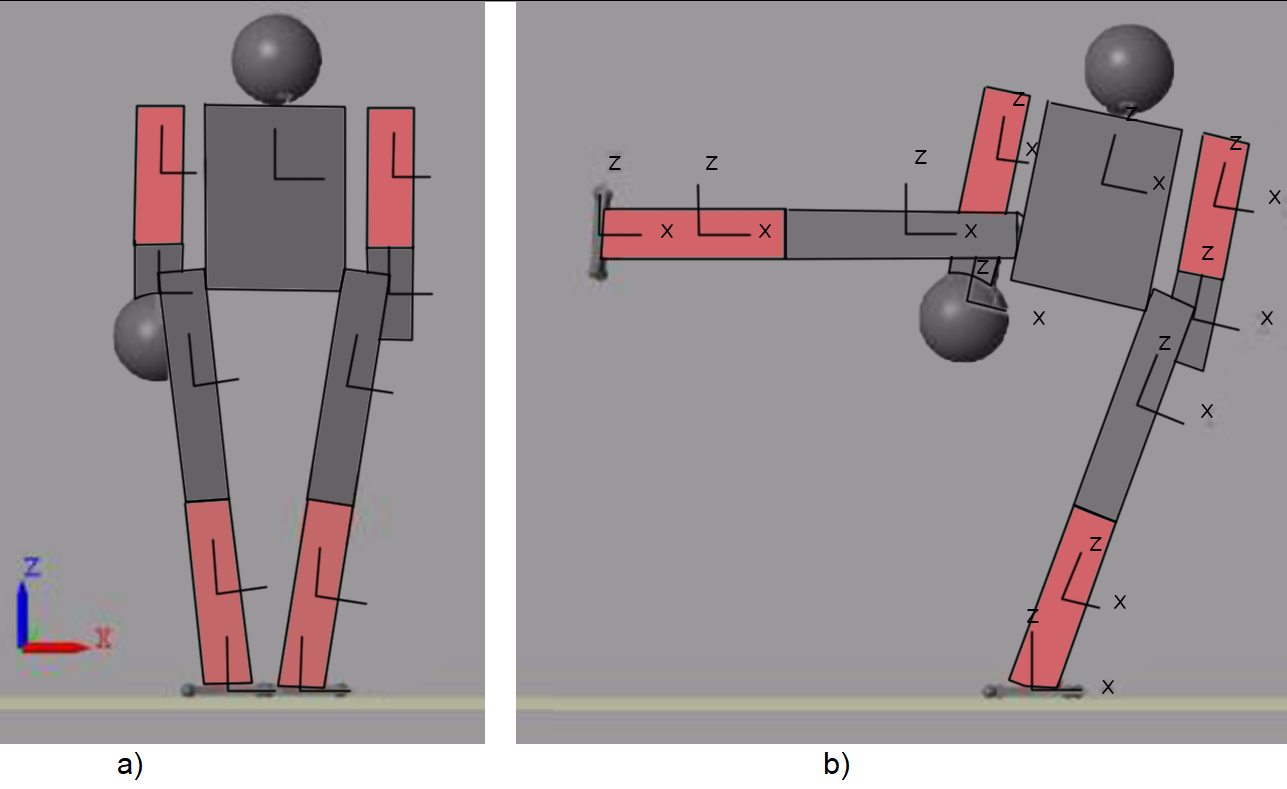

Supplement: Supplementary file 1 [file Data_Sheet_1.ZIP › figures/Move5.png]

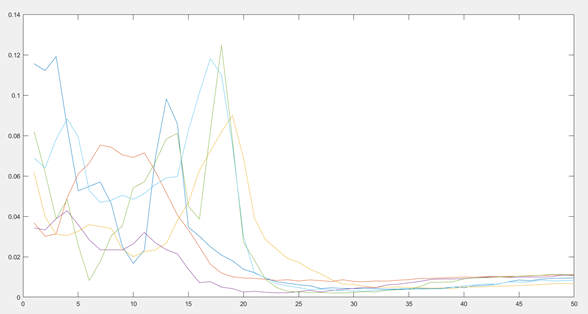

Supplement: Supplementary file 1 [file Data_Sheet_1.ZIP › figures/emedio1.png]

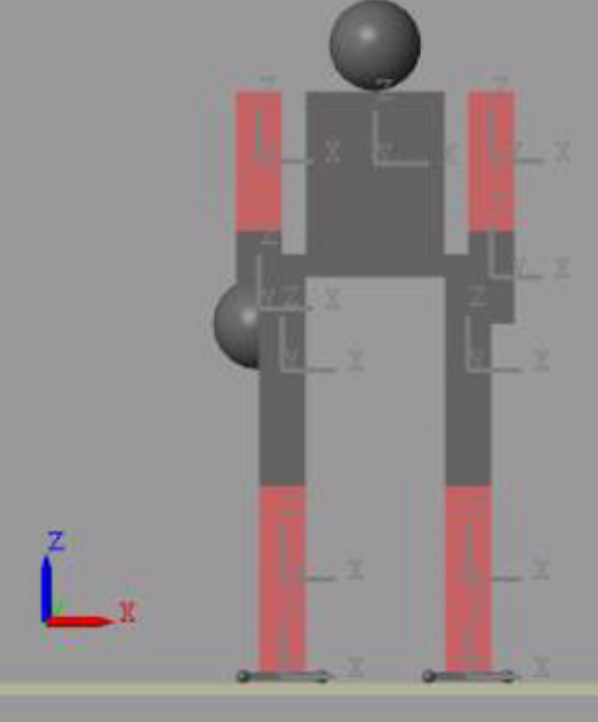

Supplement: Supplementary file 1 [file Data_Sheet_1.ZIP › figures/Position1Mass.png]

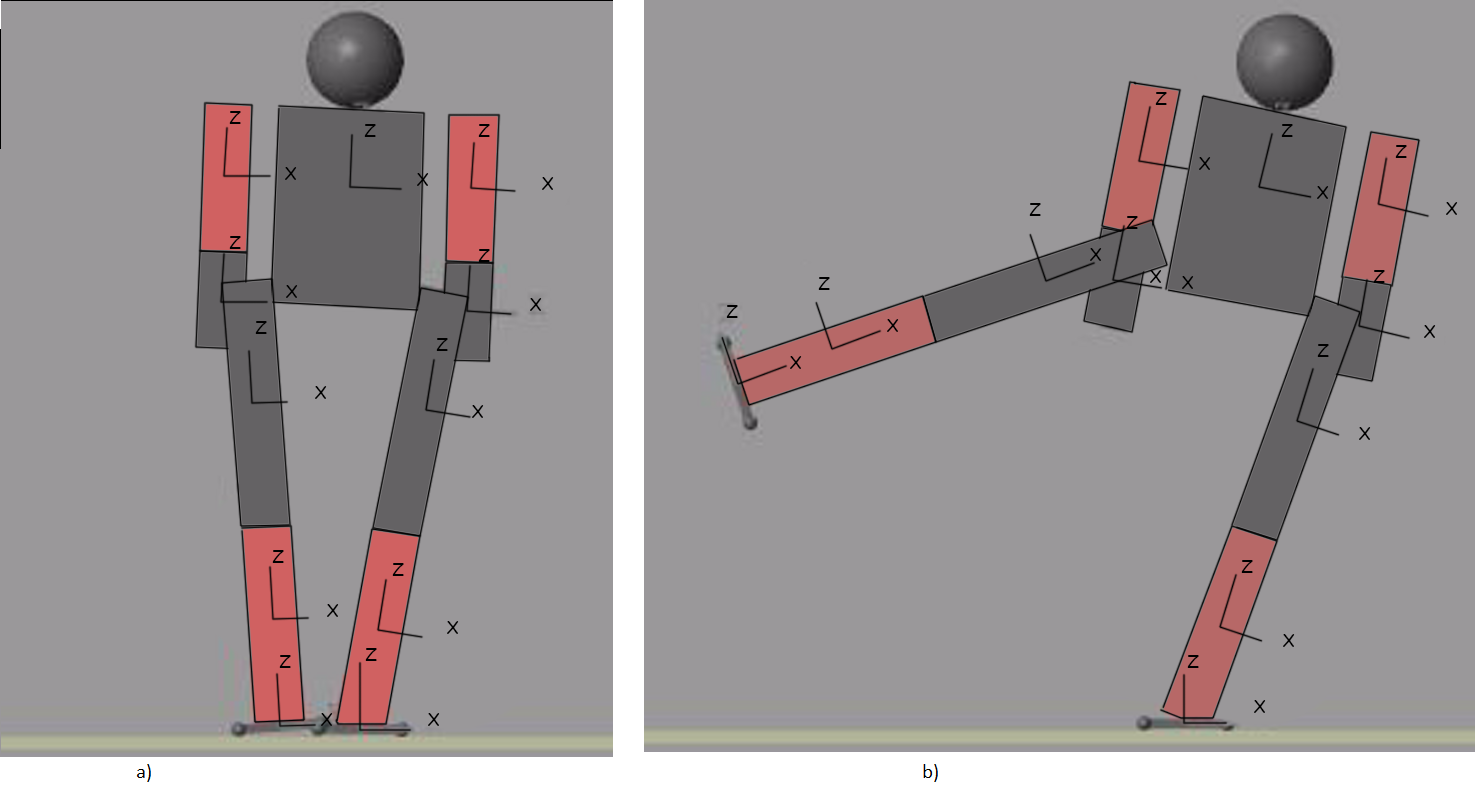

Supplement: Supplementary file 1 [file Data_Sheet_1.ZIP › figures/Move1.png]

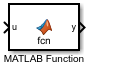

Supplement: Supplementary file 1 [file Data_Sheet_1.ZIP › figures/Matlabblock.png]

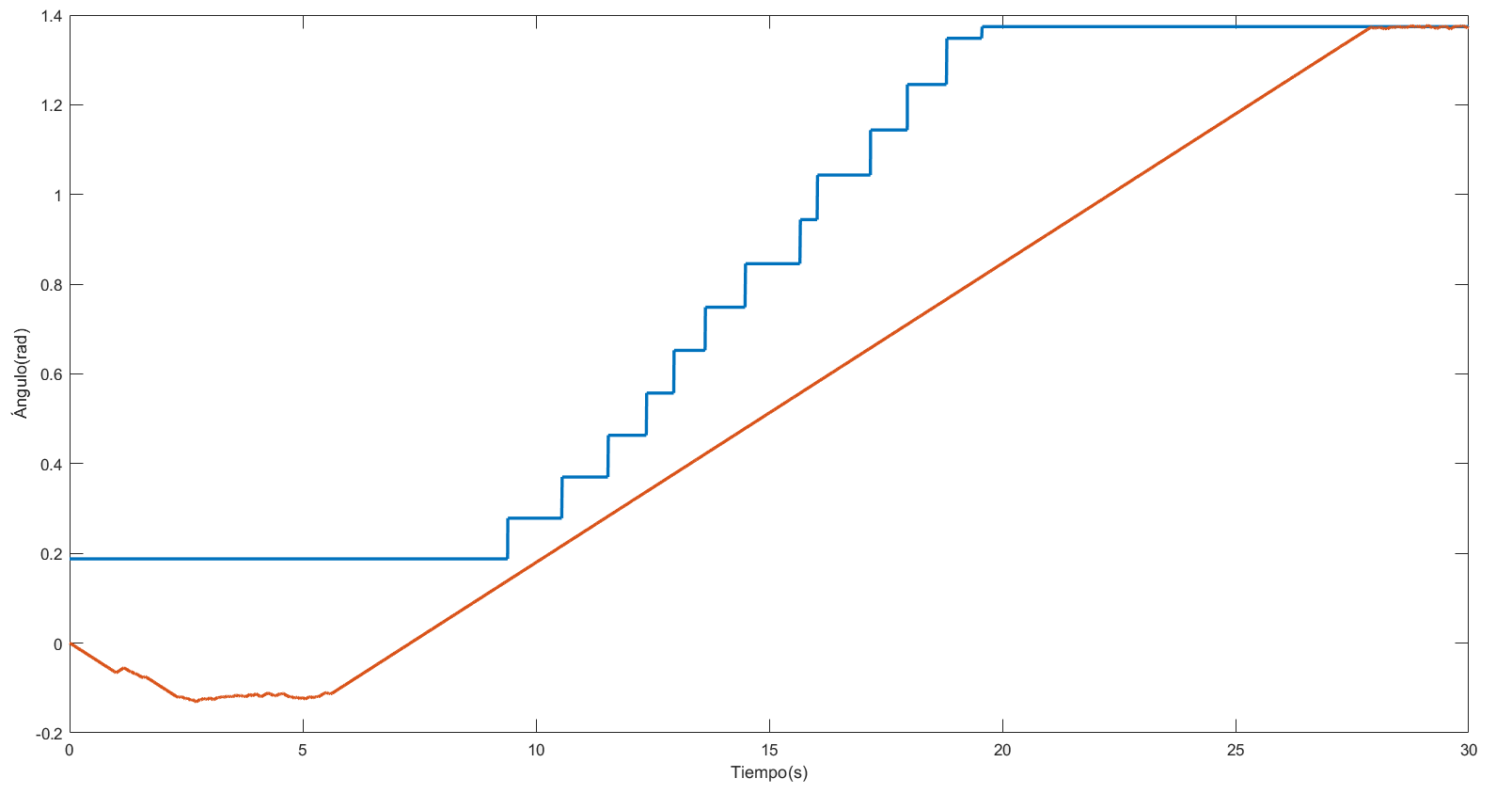

Supplement: Supplementary file 1 [file Data_Sheet_1.ZIP › figures/MovementPath3.png]

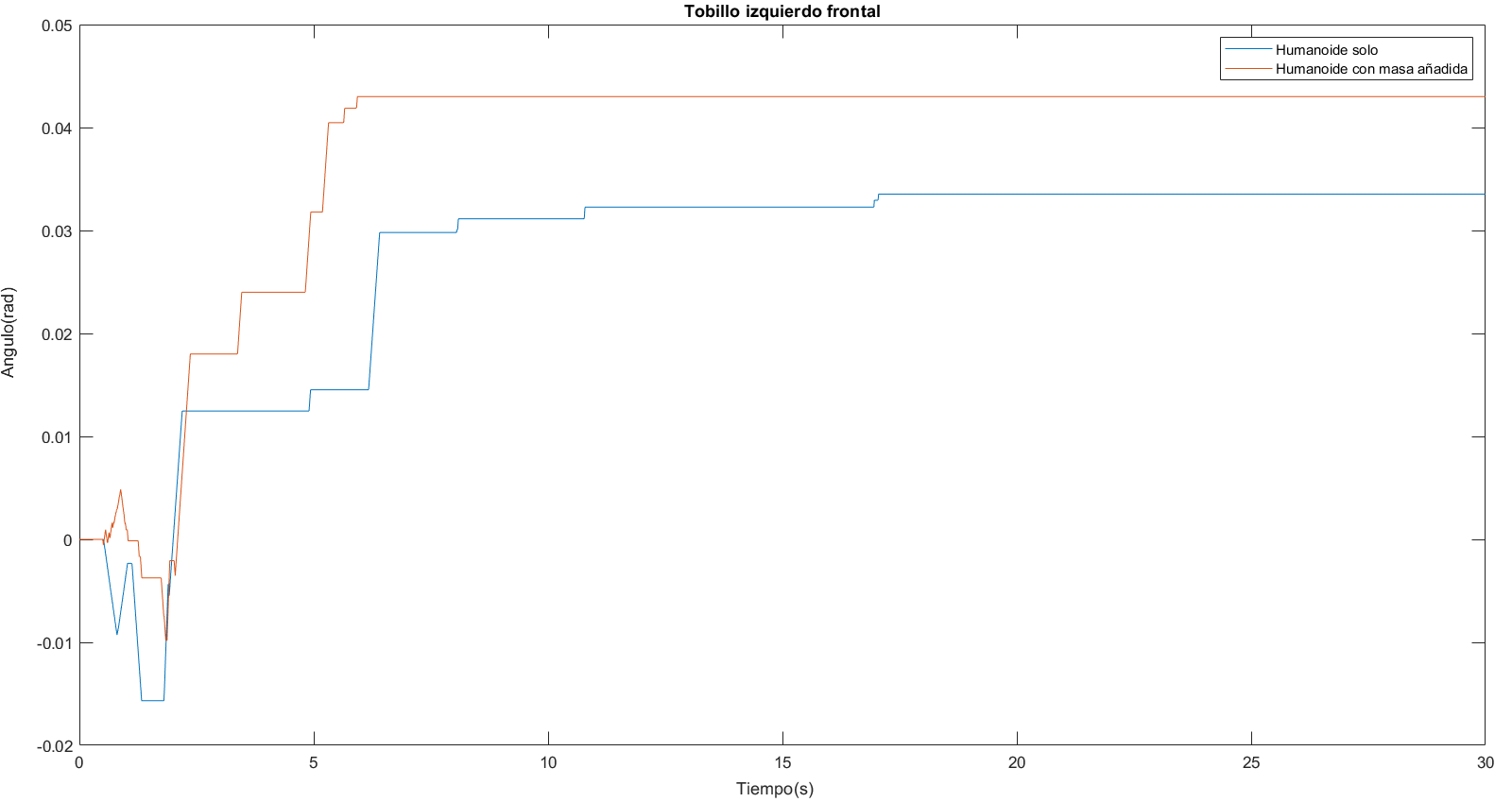

Supplement: Supplementary file 1 [file Data_Sheet_1.ZIP › figures/ComparisonArt8.png]

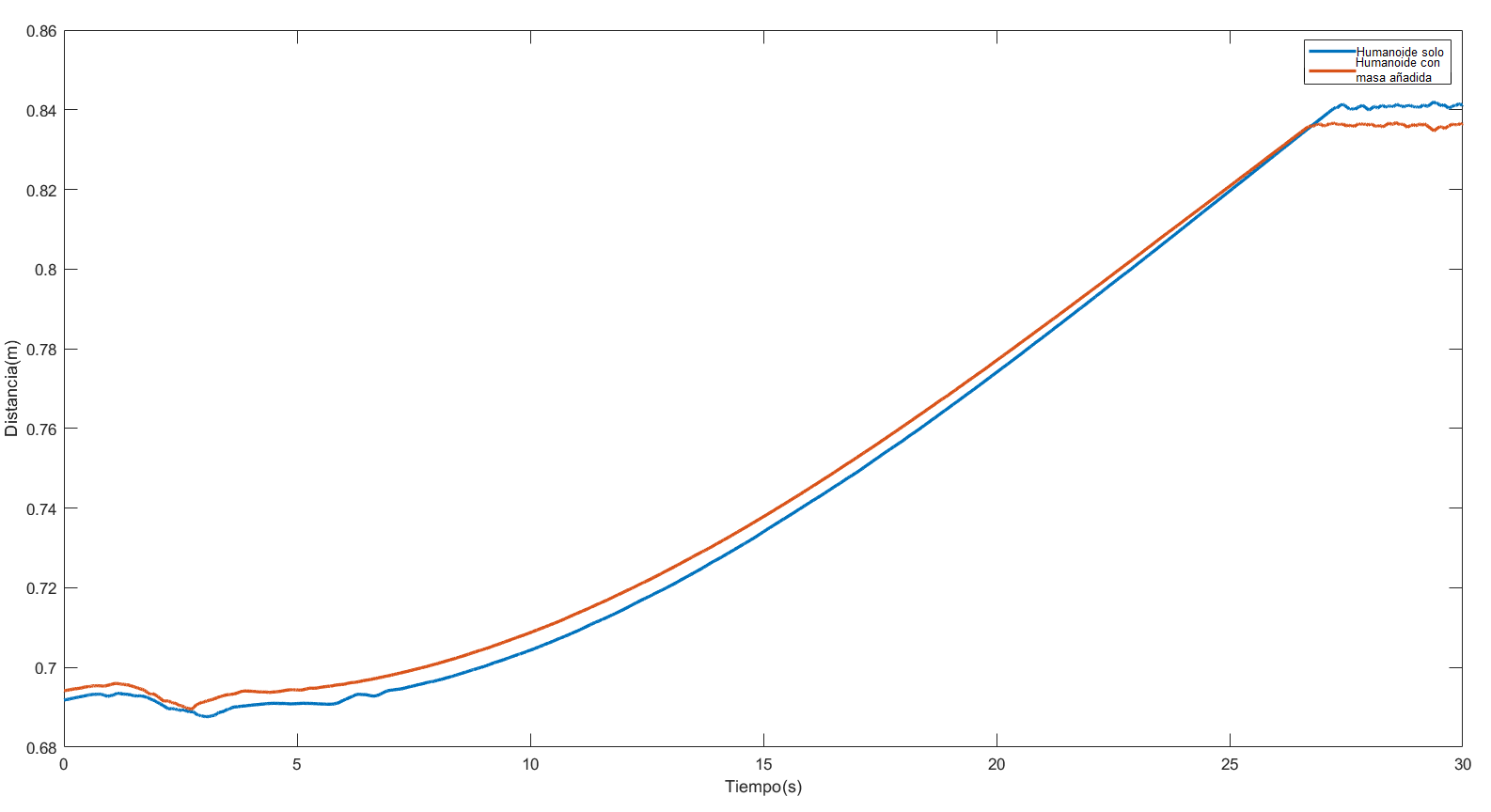

Supplement: Supplementary file 1 [file Data_Sheet_1.ZIP › figures/ComparisonCMZ2.png]

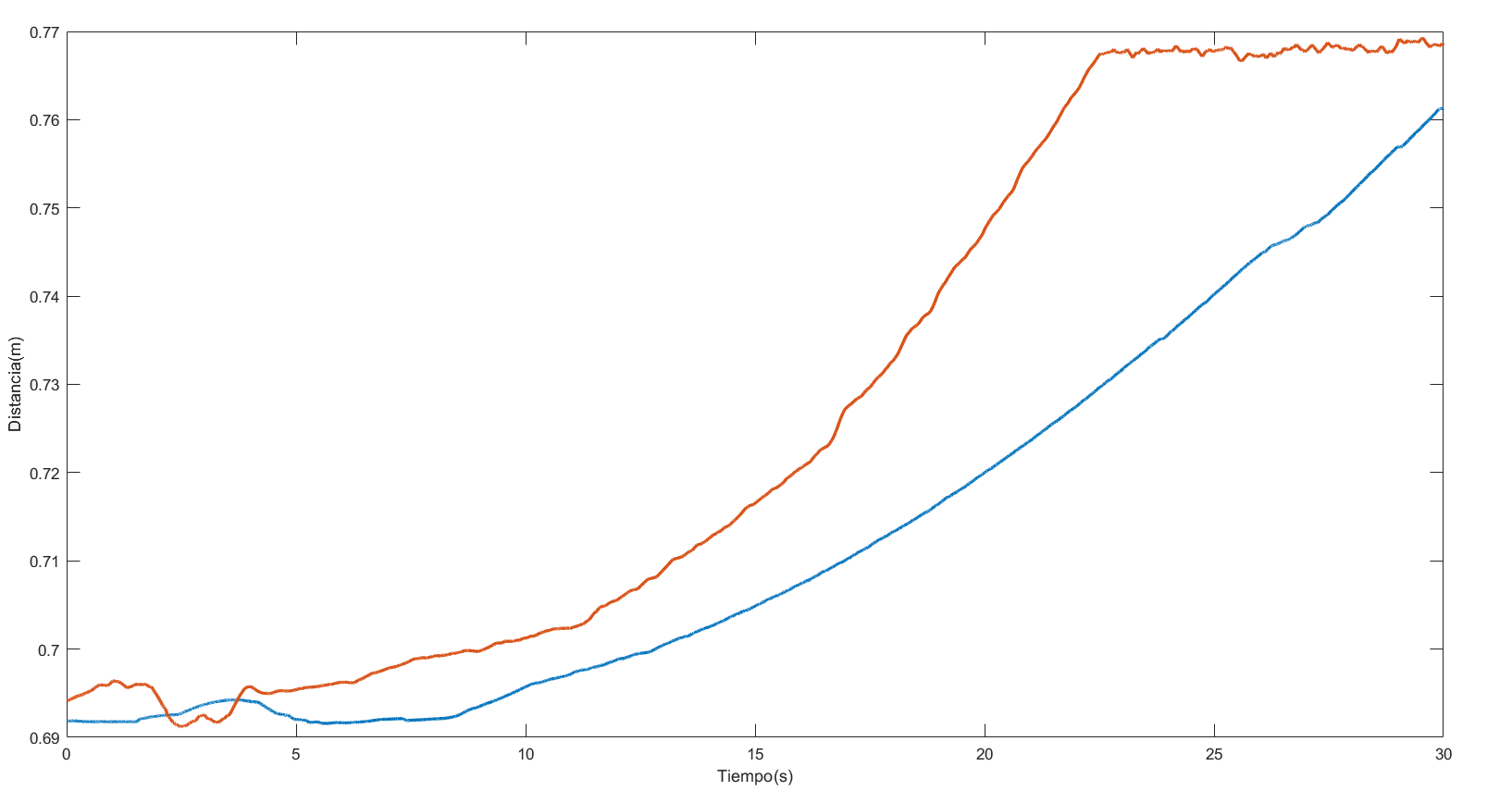

Supplement: Supplementary file 1 [file Data_Sheet_1.ZIP › figures/ComparisonCMZ3.png]

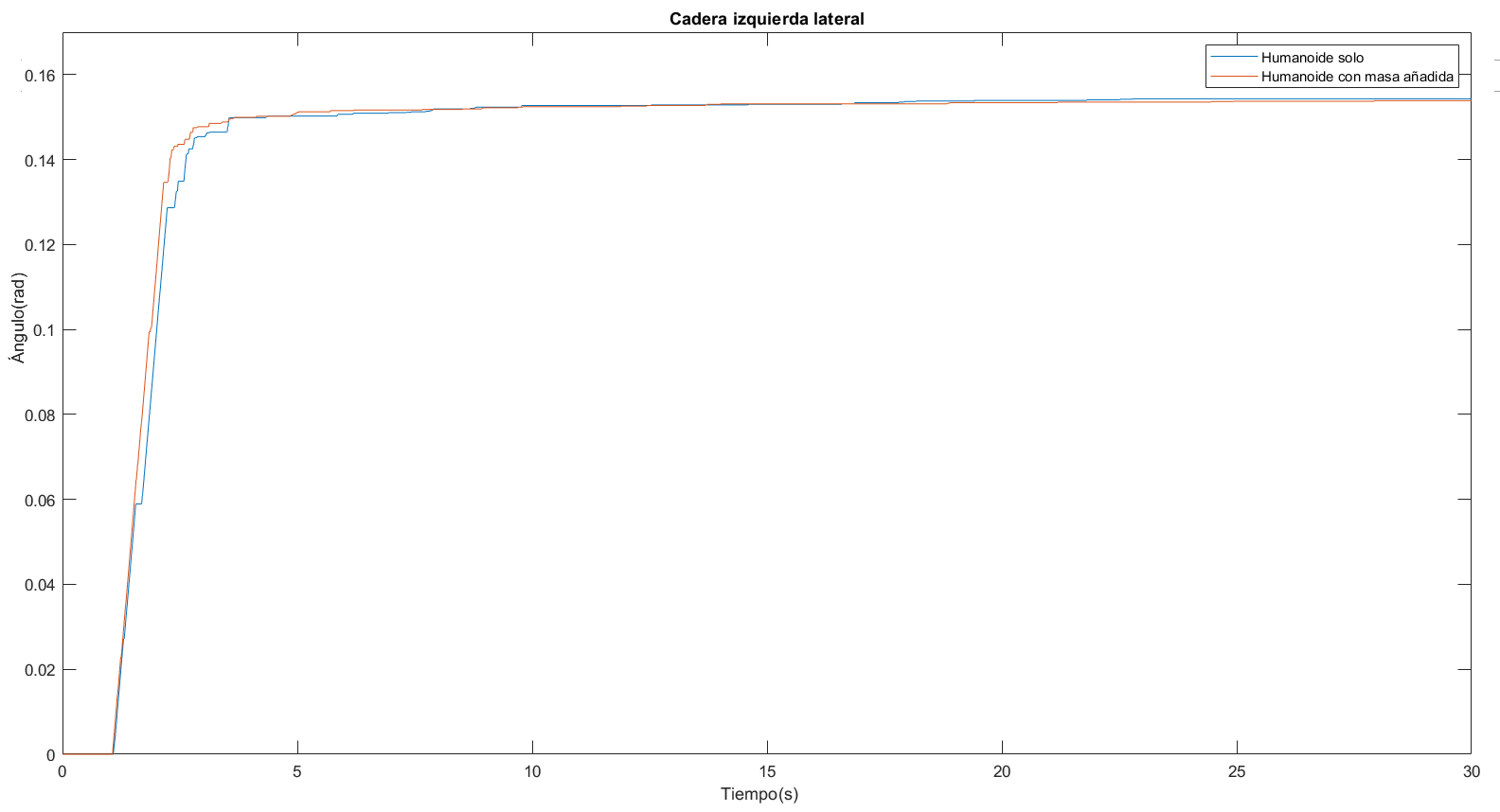

Supplement: Supplementary file 1 [file Data_Sheet_1.ZIP › figures/ComparisonArt9.png]

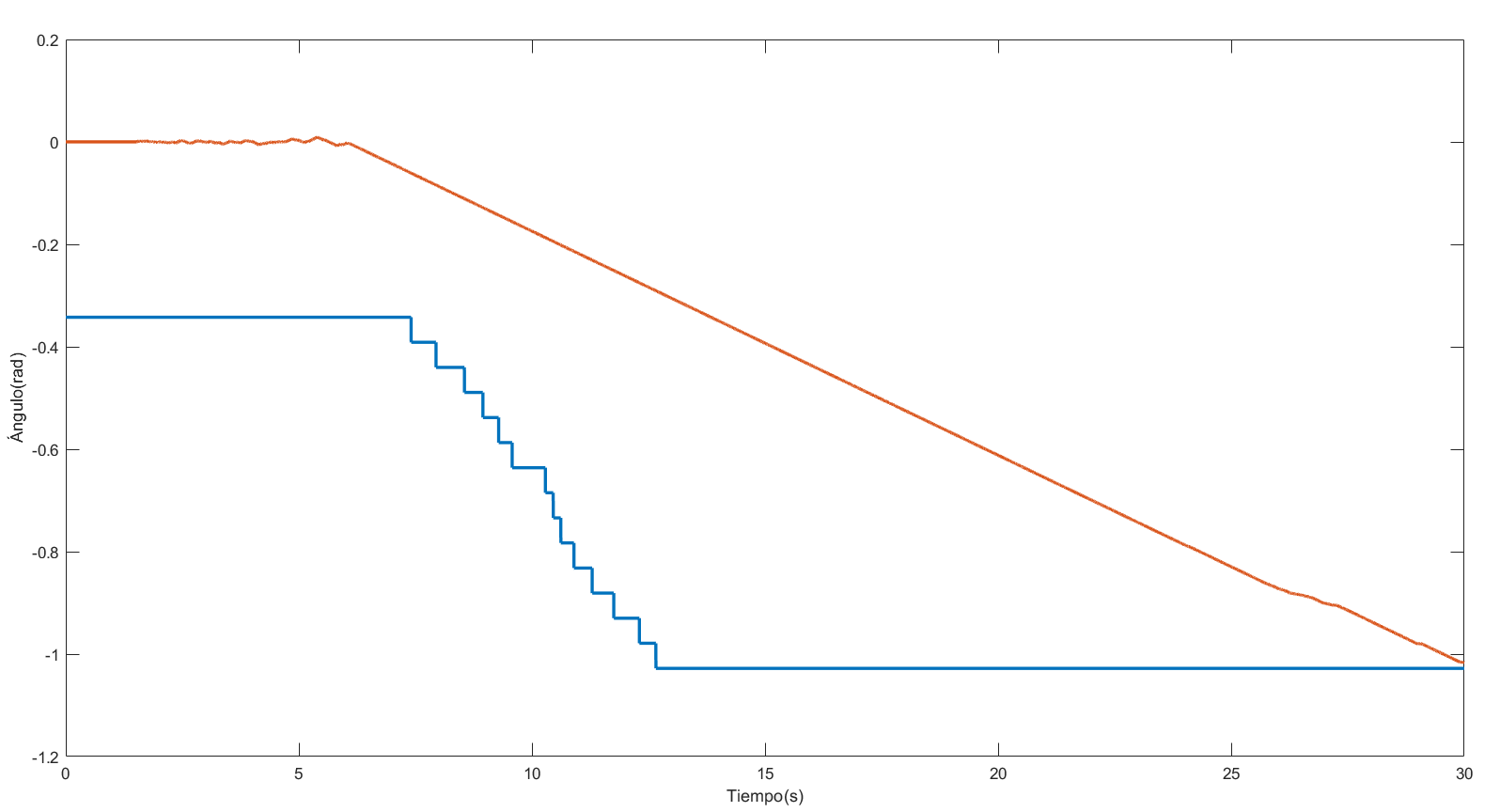

Supplement: Supplementary file 1 [file Data_Sheet_1.ZIP › figures/MovementPath2.png]

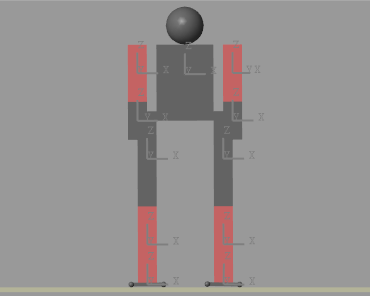

Supplement: Supplementary file 1 [file Data_Sheet_1.ZIP › figures/SimulinkModel.png]

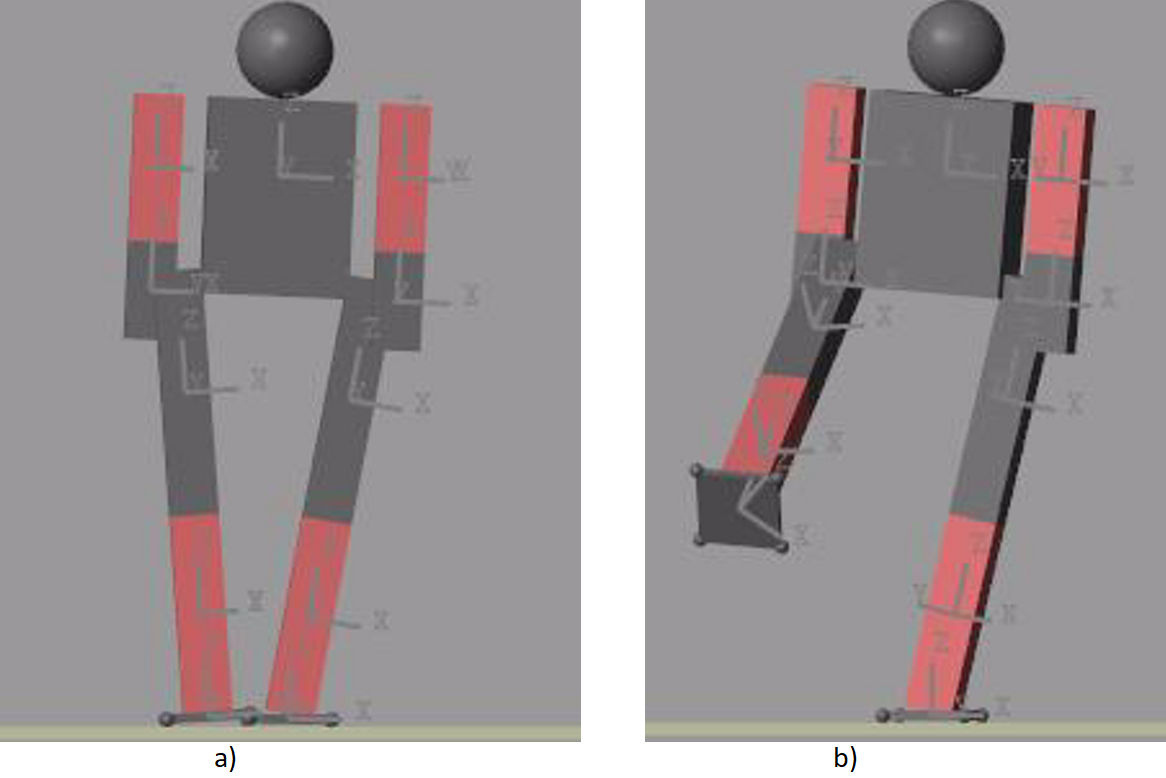

Supplement: Supplementary file 1 [file Data_Sheet_1.ZIP › figures/Move2.png]

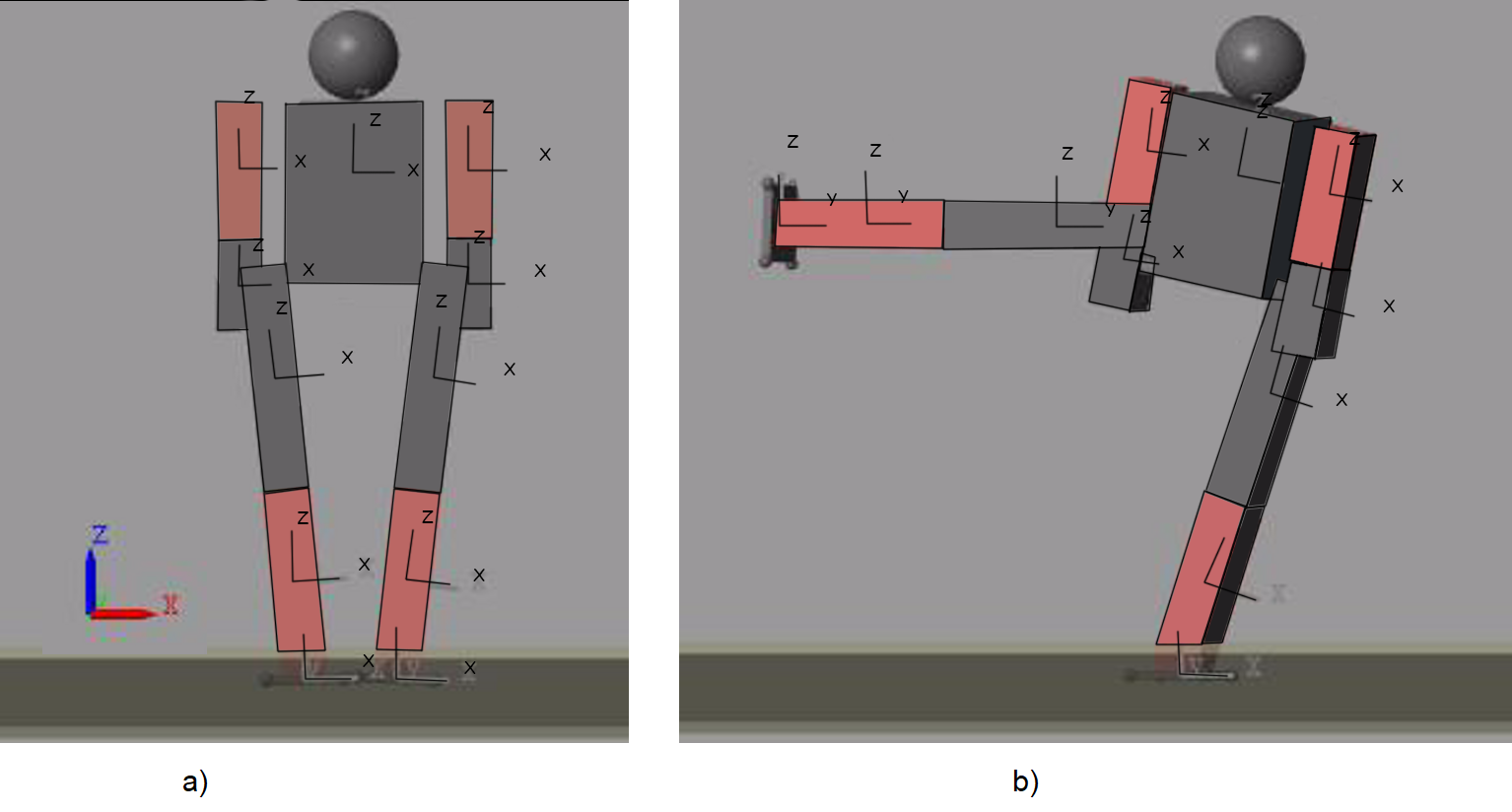

Supplement: Supplementary file 1 [file Data_Sheet_1.ZIP › figures/Move3.png]

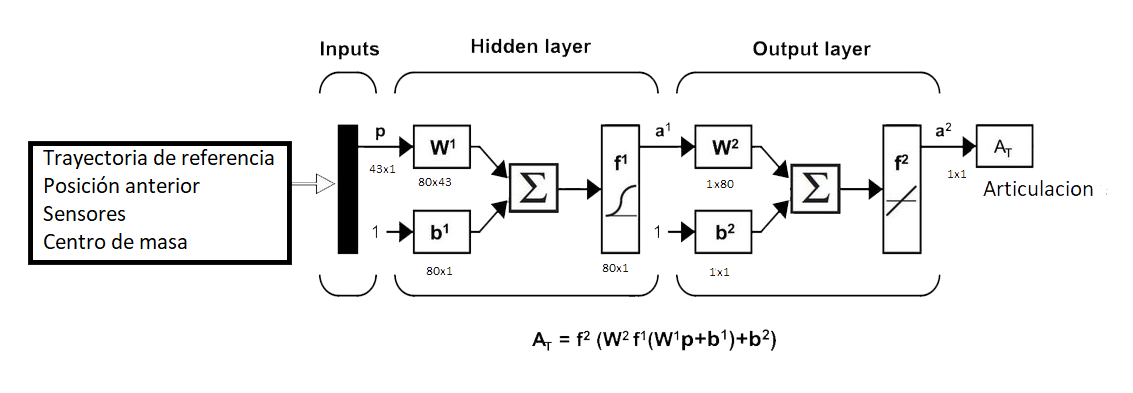

Supplement: Supplementary file 1 [file Data_Sheet_1.ZIP › figures/ArquitecturaNeurona.png]

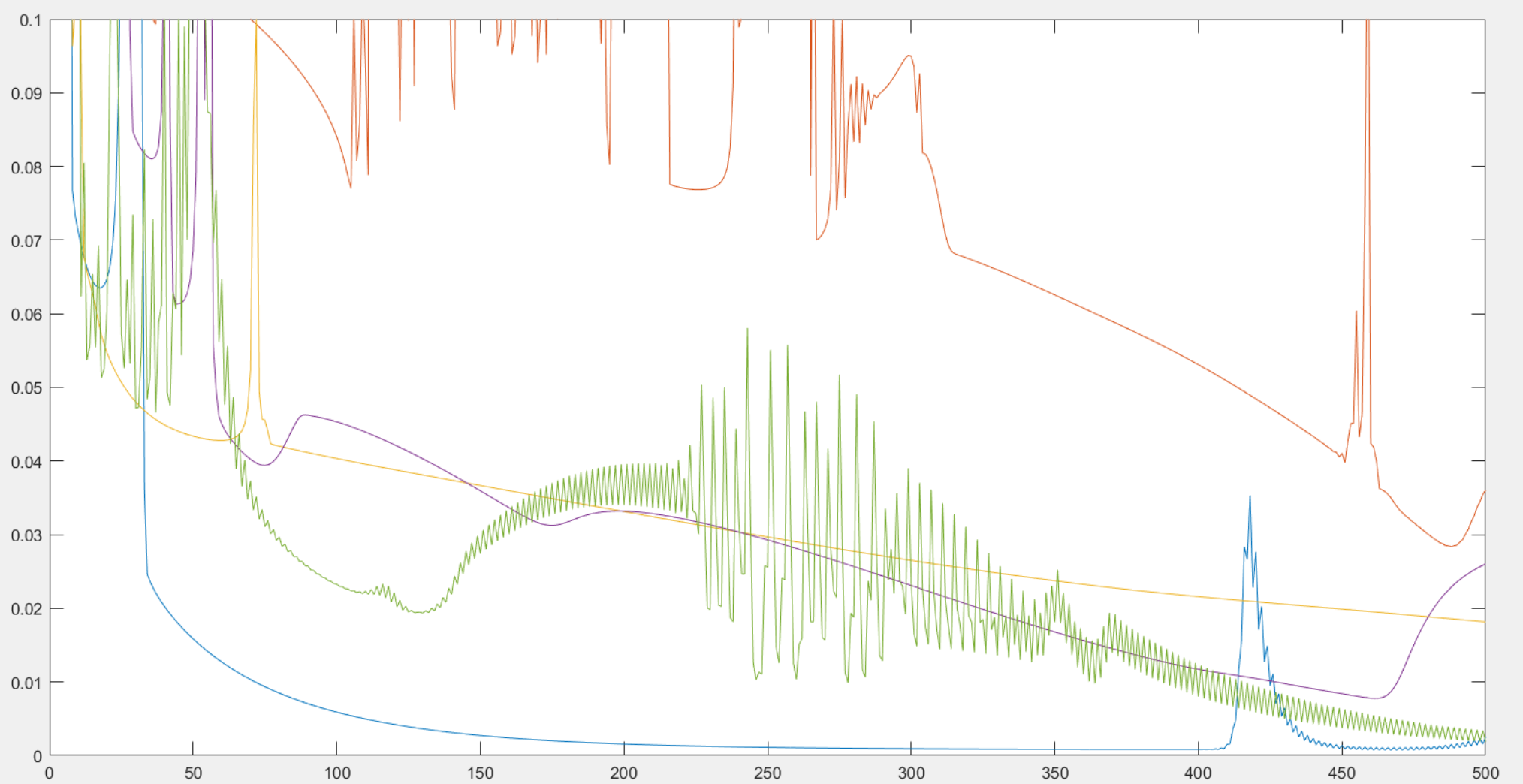

Supplement: Supplementary file 1 [file Data_Sheet_1.ZIP › figures/WithoutAnnealing.png]

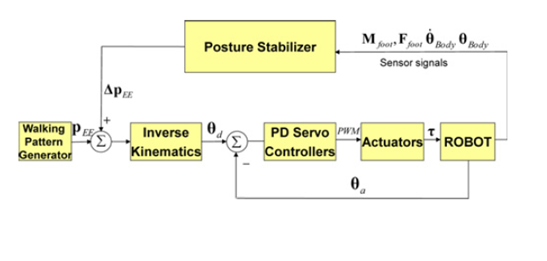

Supplement: Supplementary file 1 [file Data_Sheet_1.ZIP › figures/WalkingAlgorithmFramework.png]

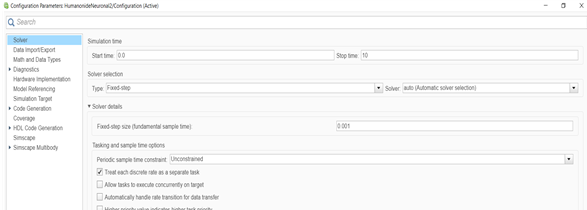

Supplement: Supplementary file 1 [file Data_Sheet_1.ZIP › figures/SimulinkConfig2.png]

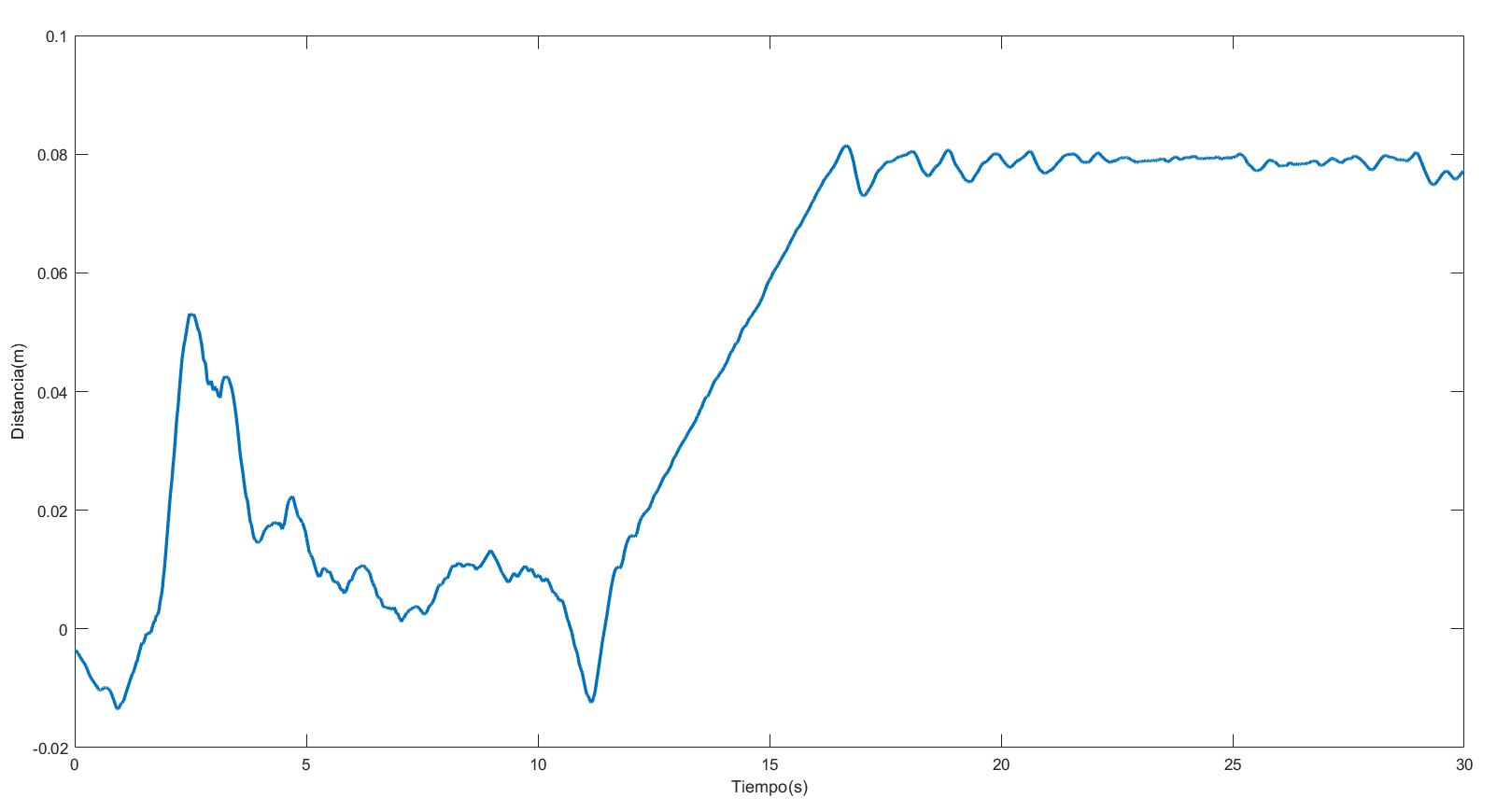

Supplement: Supplementary file 1 [file Data_Sheet_1.ZIP › figures/TrajectoryCMY5.png]

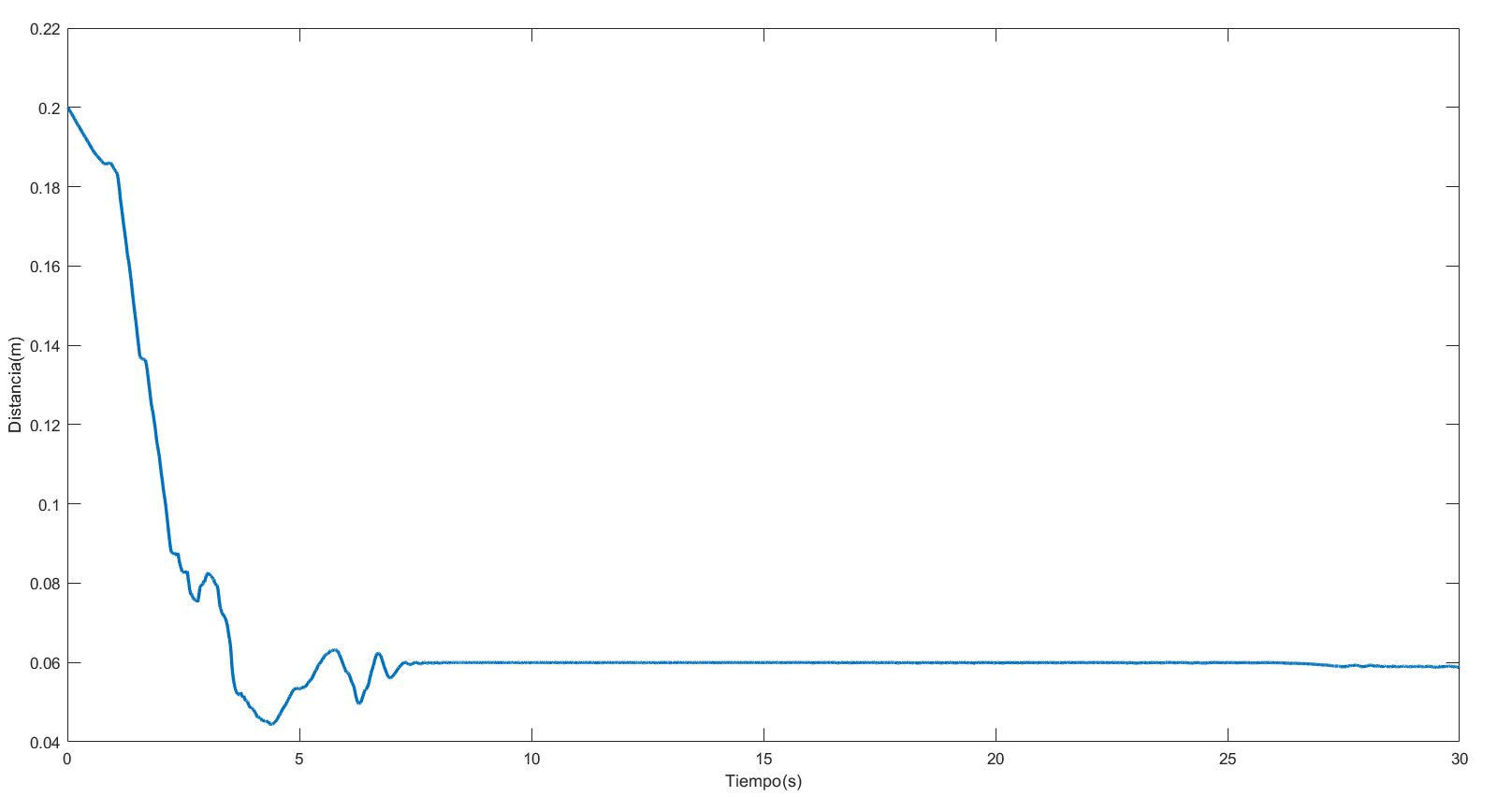

Supplement: Supplementary file 1 [file Data_Sheet_1.ZIP › figures/TrajectoryCMX1.png]

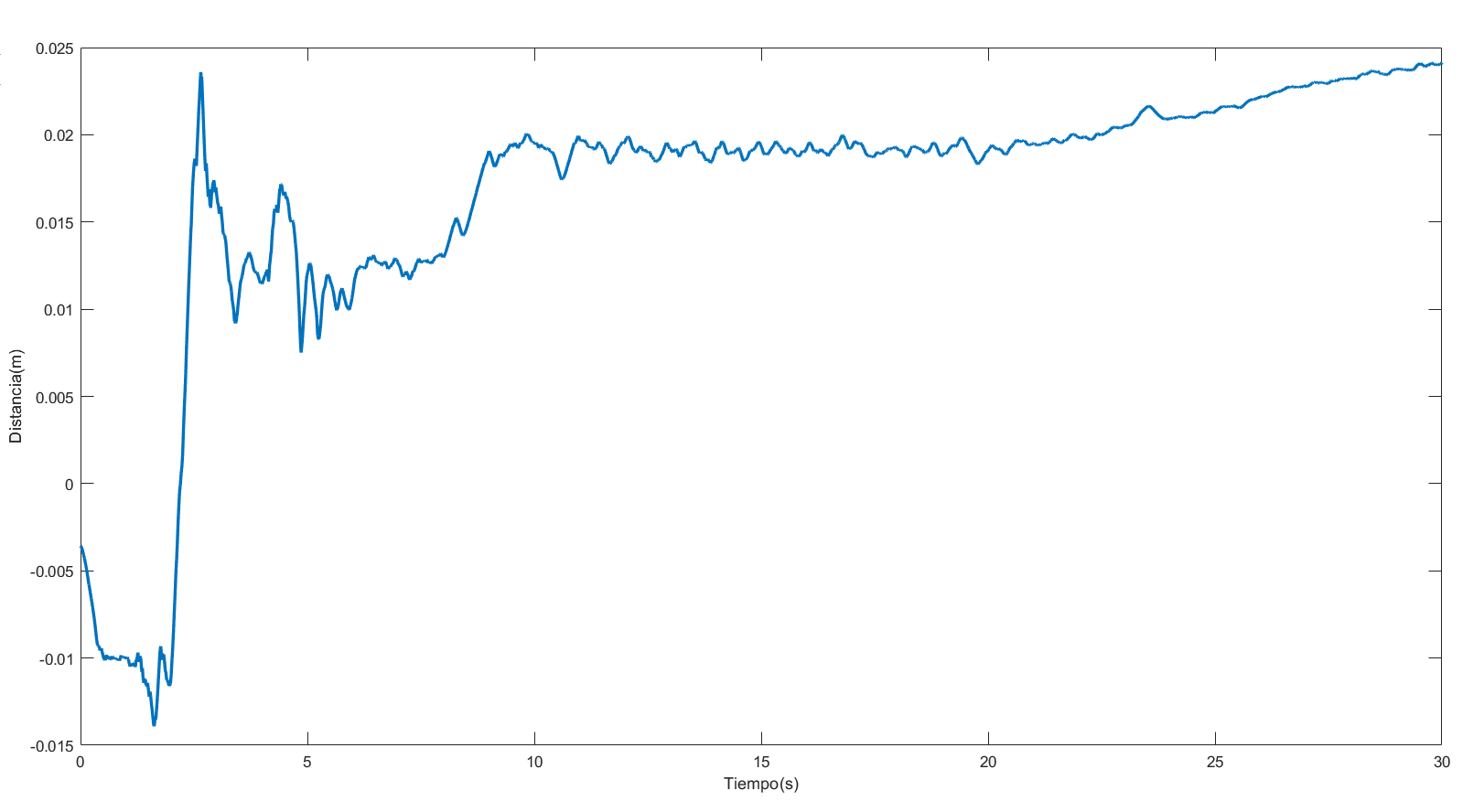

Supplement: Supplementary file 1 [file Data_Sheet_1.ZIP › figures/TrajectoryCMY4.png]

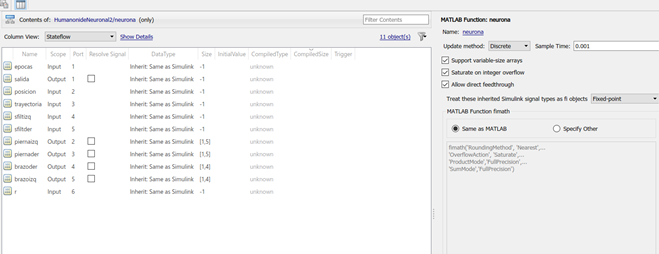

Supplement: Supplementary file 1 [file Data_Sheet_1.ZIP › figures/SimulinkConfig3.png]

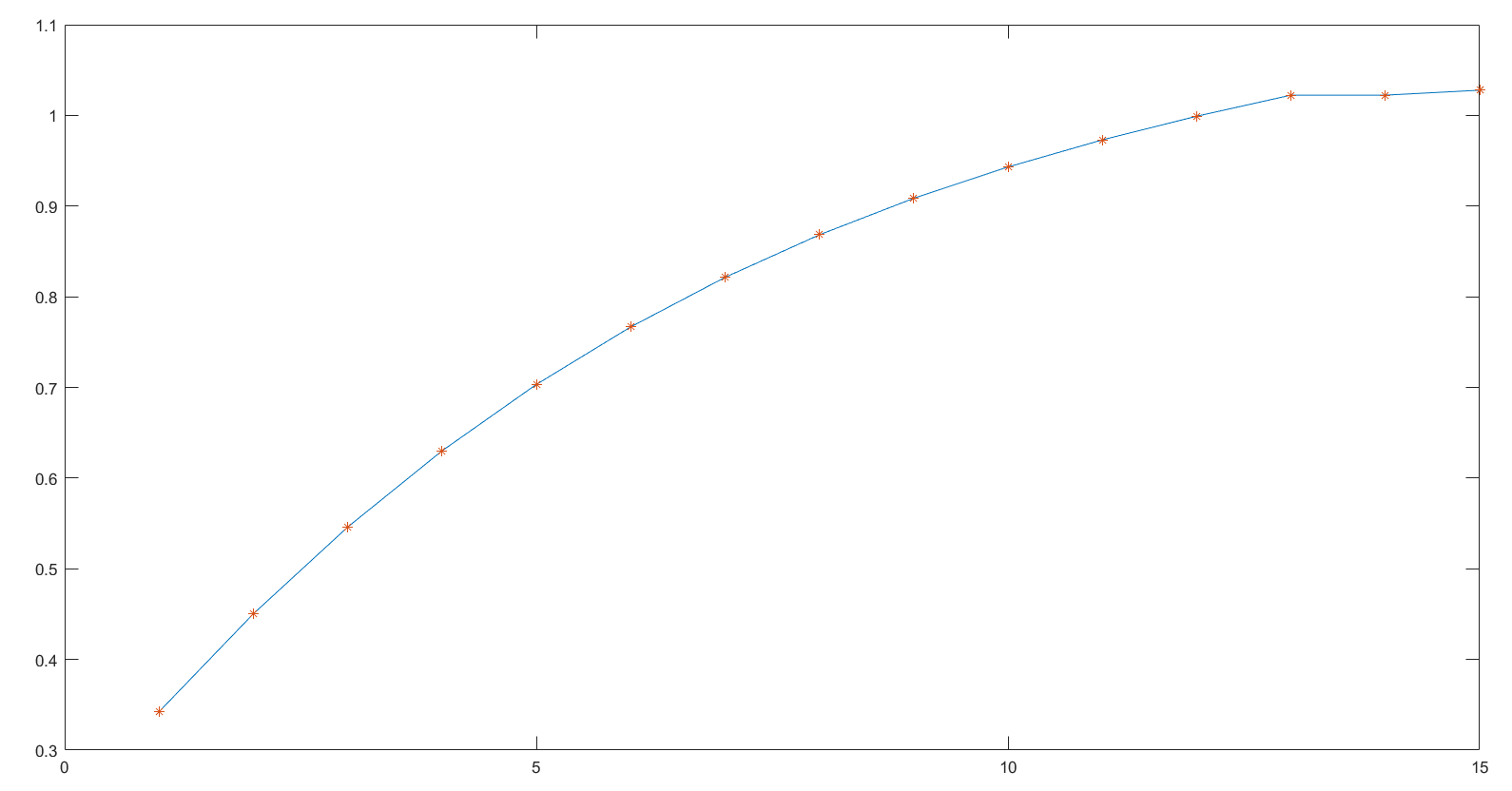

Supplement: Supplementary file 1 [file Data_Sheet_1.ZIP › figures/Vector.png]

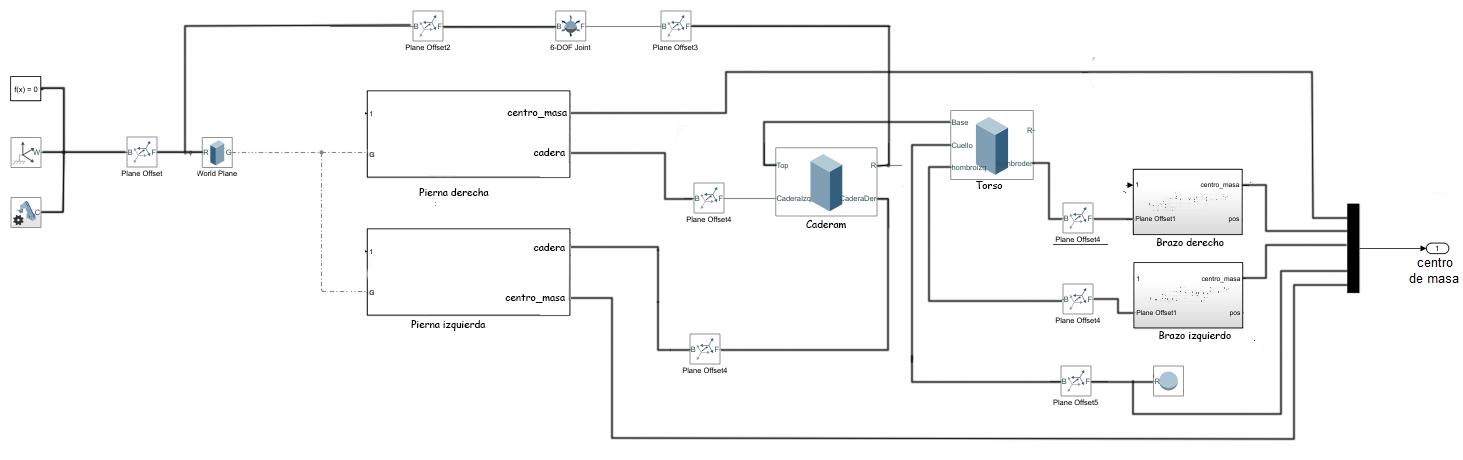

Supplement: Supplementary file 1 [file Data_Sheet_1.ZIP › figures/Blocks1.png]

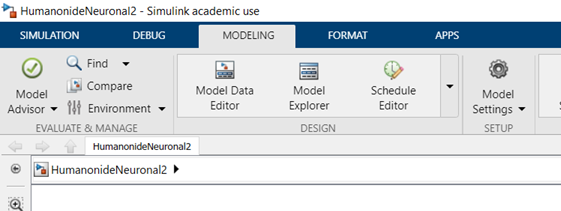

Supplement: Supplementary file 1 [file Data_Sheet_1.ZIP › figures/SimulinkConfig1.png]

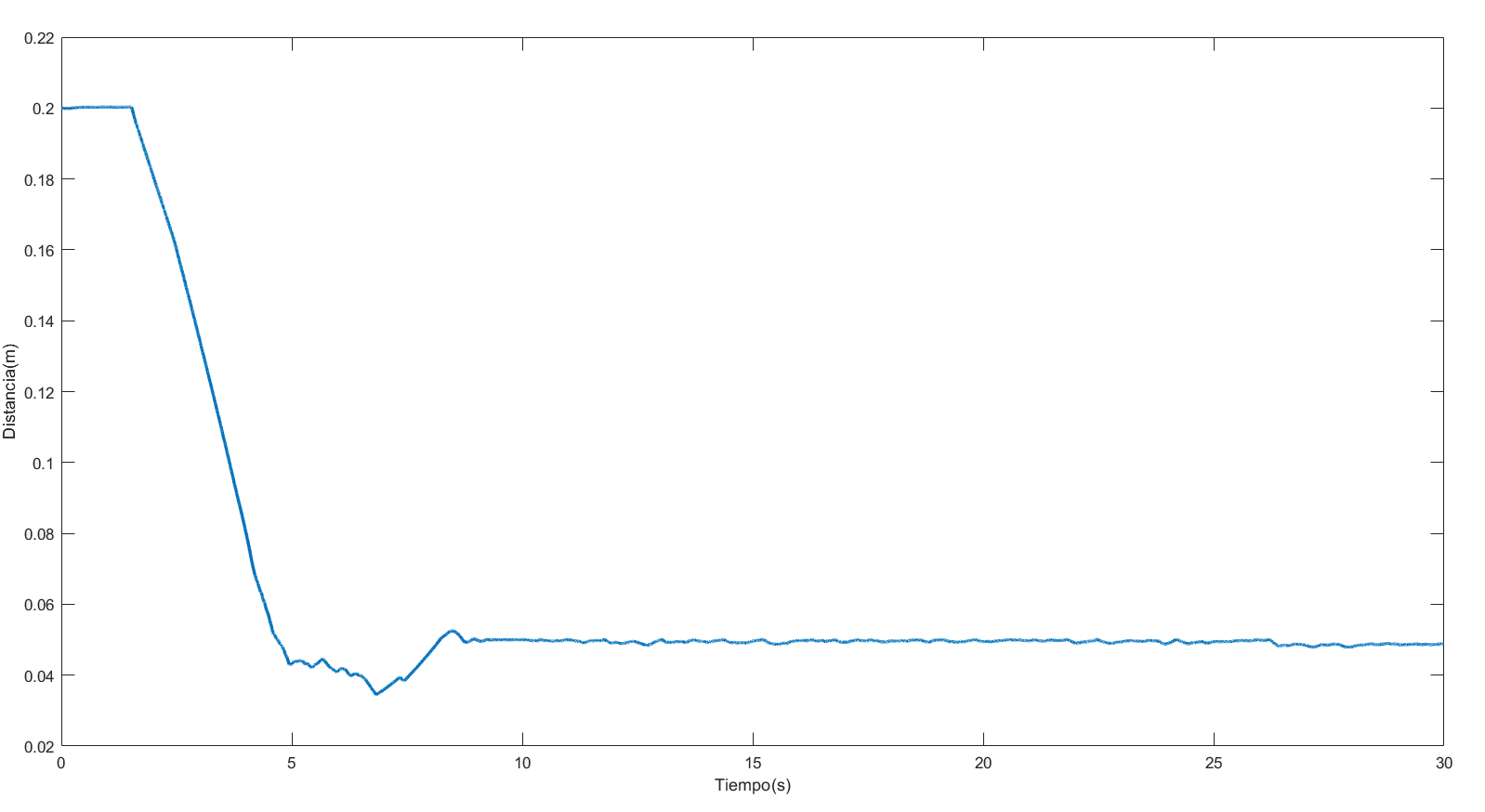

Supplement: Supplementary file 1 [file Data_Sheet_1.ZIP › figures/TrajectoryCMX2.png]

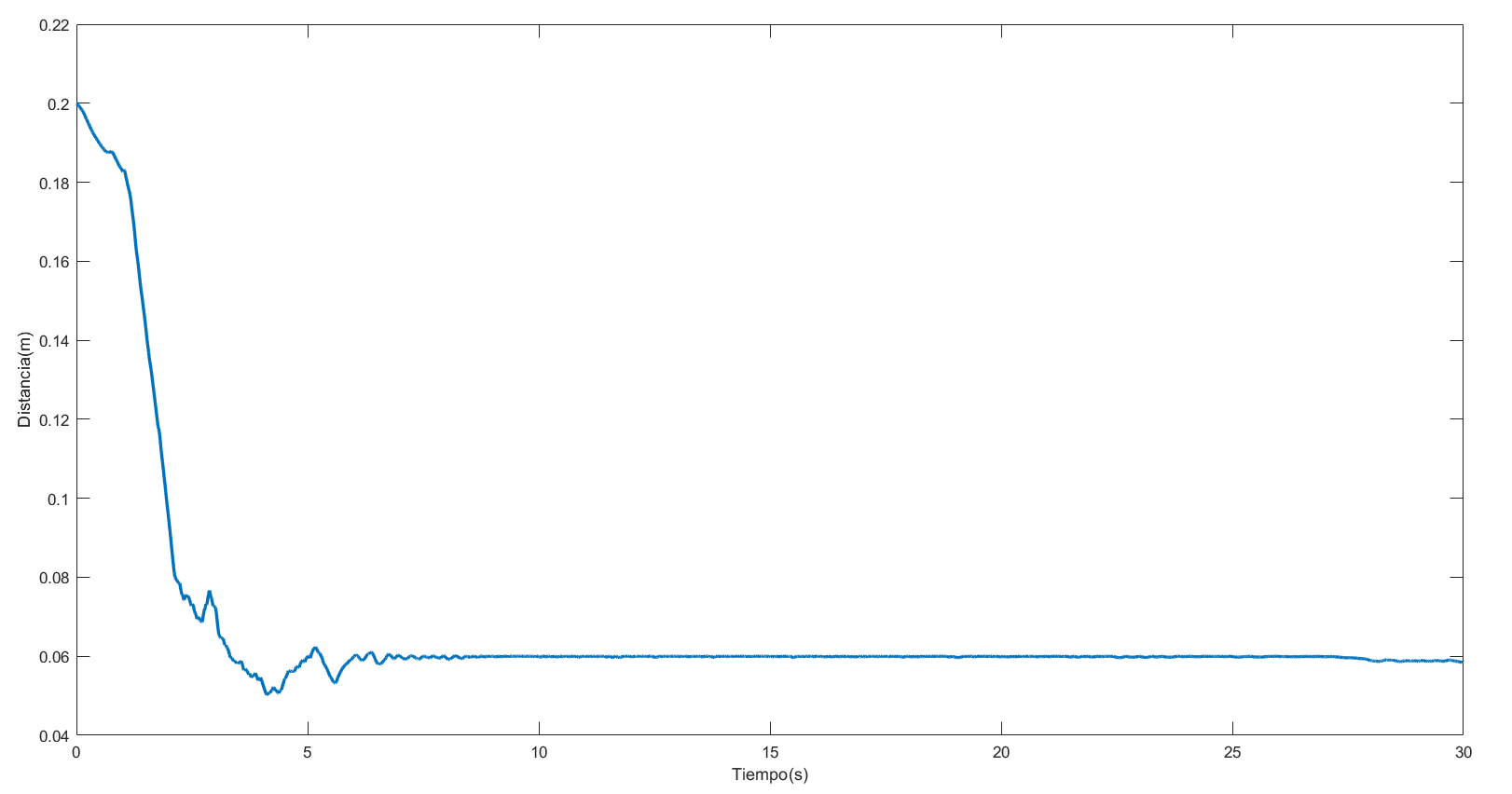

Supplement: Supplementary file 1 [file Data_Sheet_1.ZIP › figures/TrajectoryCMX3.png]

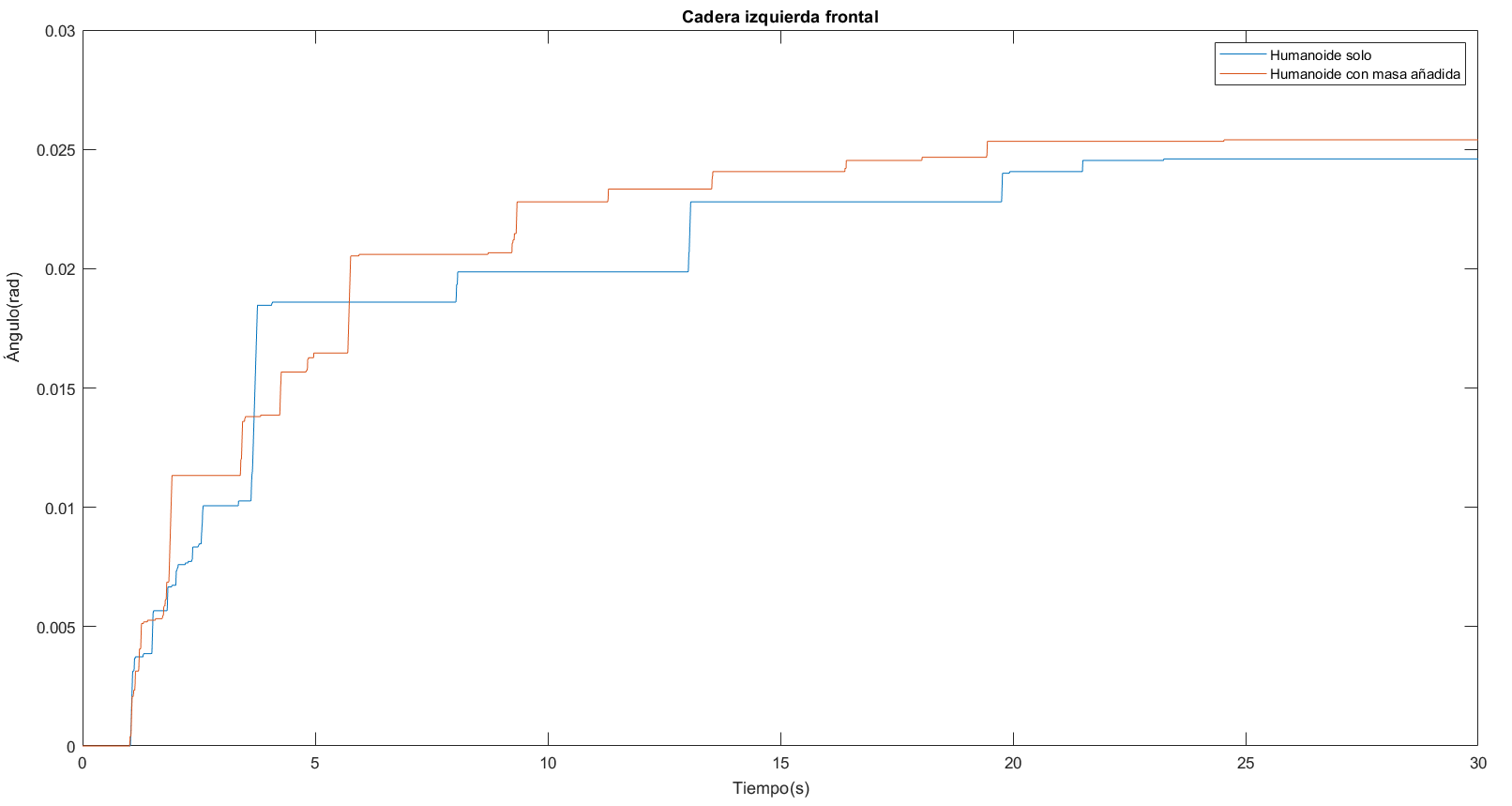

Supplement: Supplementary file 1 [file Data_Sheet_1.ZIP › figures/ComparisonArt10.png]

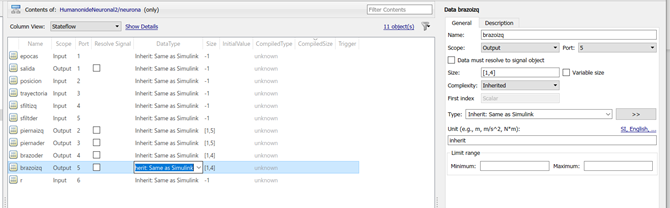

Supplement: Supplementary file 1 [file Data_Sheet_1.ZIP › figures/SimulinkConfig4.png]

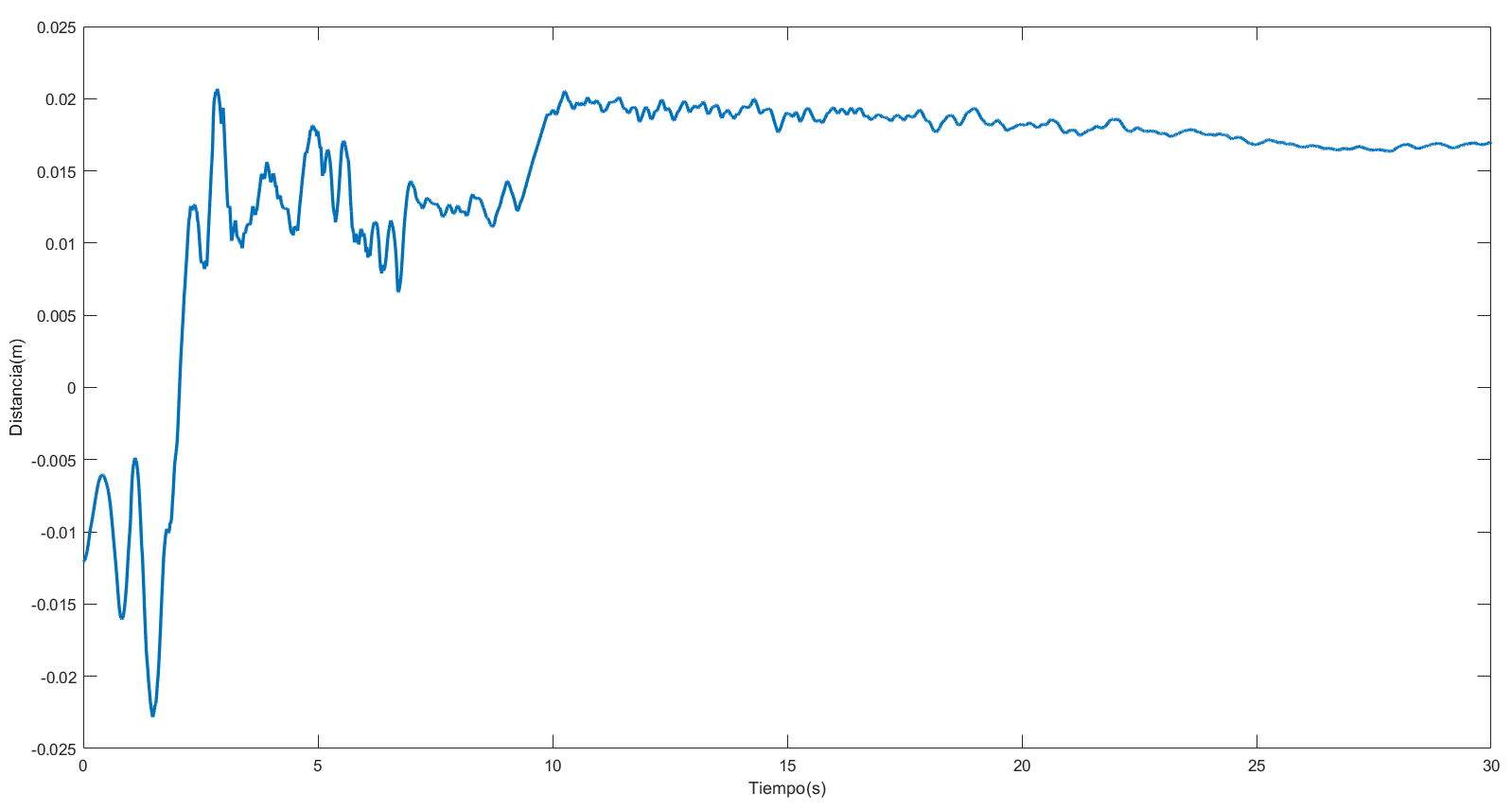

Supplement: Supplementary file 1 [file Data_Sheet_1.ZIP › figures/TrajectoryCMY3.png]

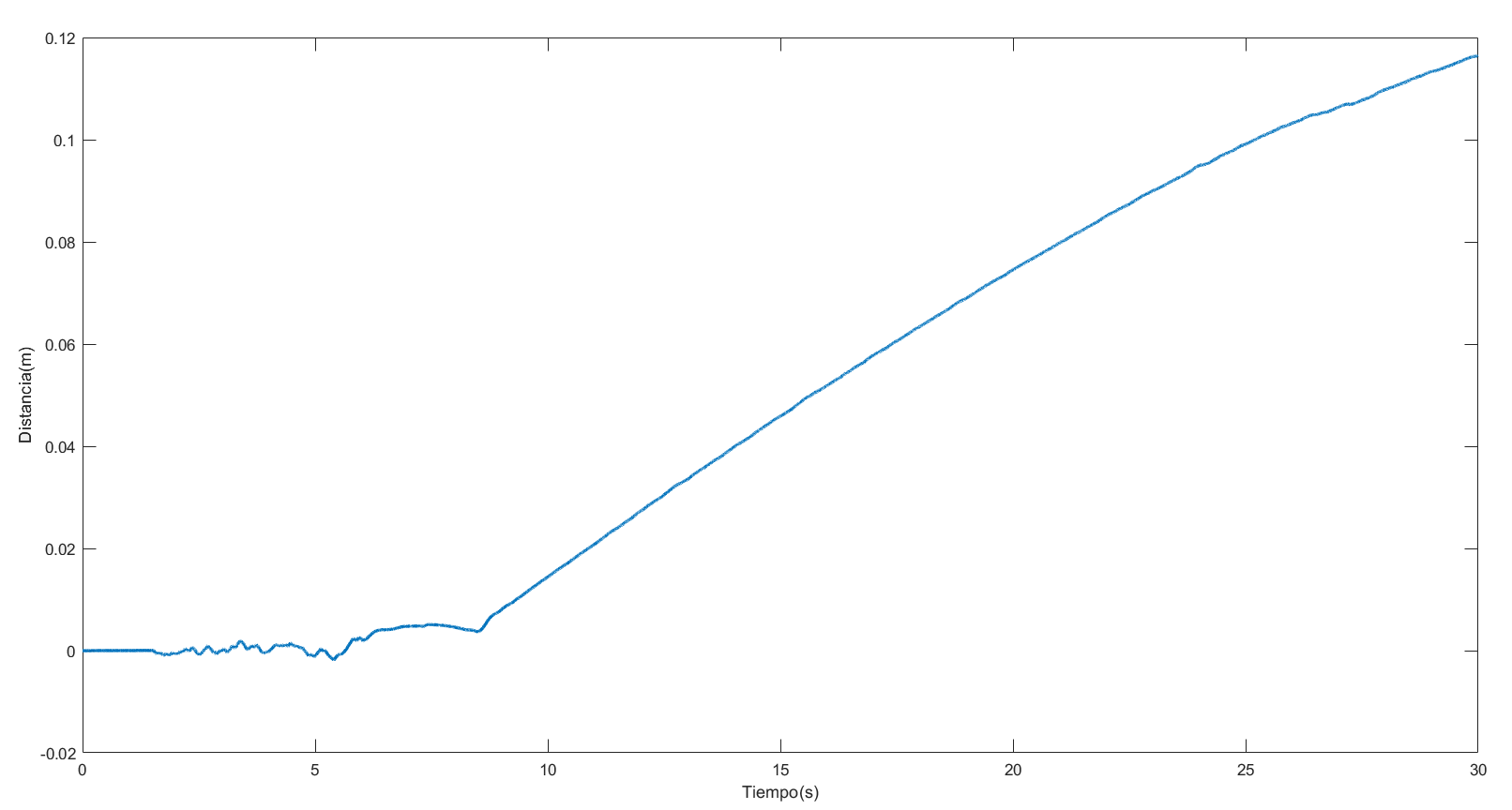

Supplement: Supplementary file 1 [file Data_Sheet_1.ZIP › figures/TrajectoryCMY2.png]

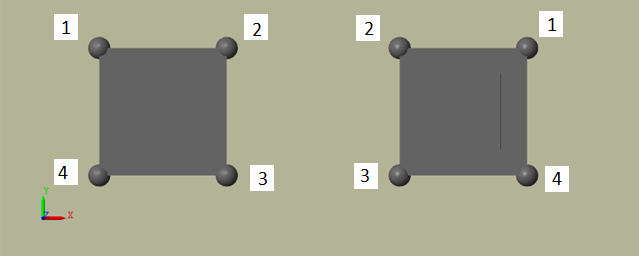

Supplement: Supplementary file 1 [file Data_Sheet_1.ZIP › figures/PositionSensors.png]

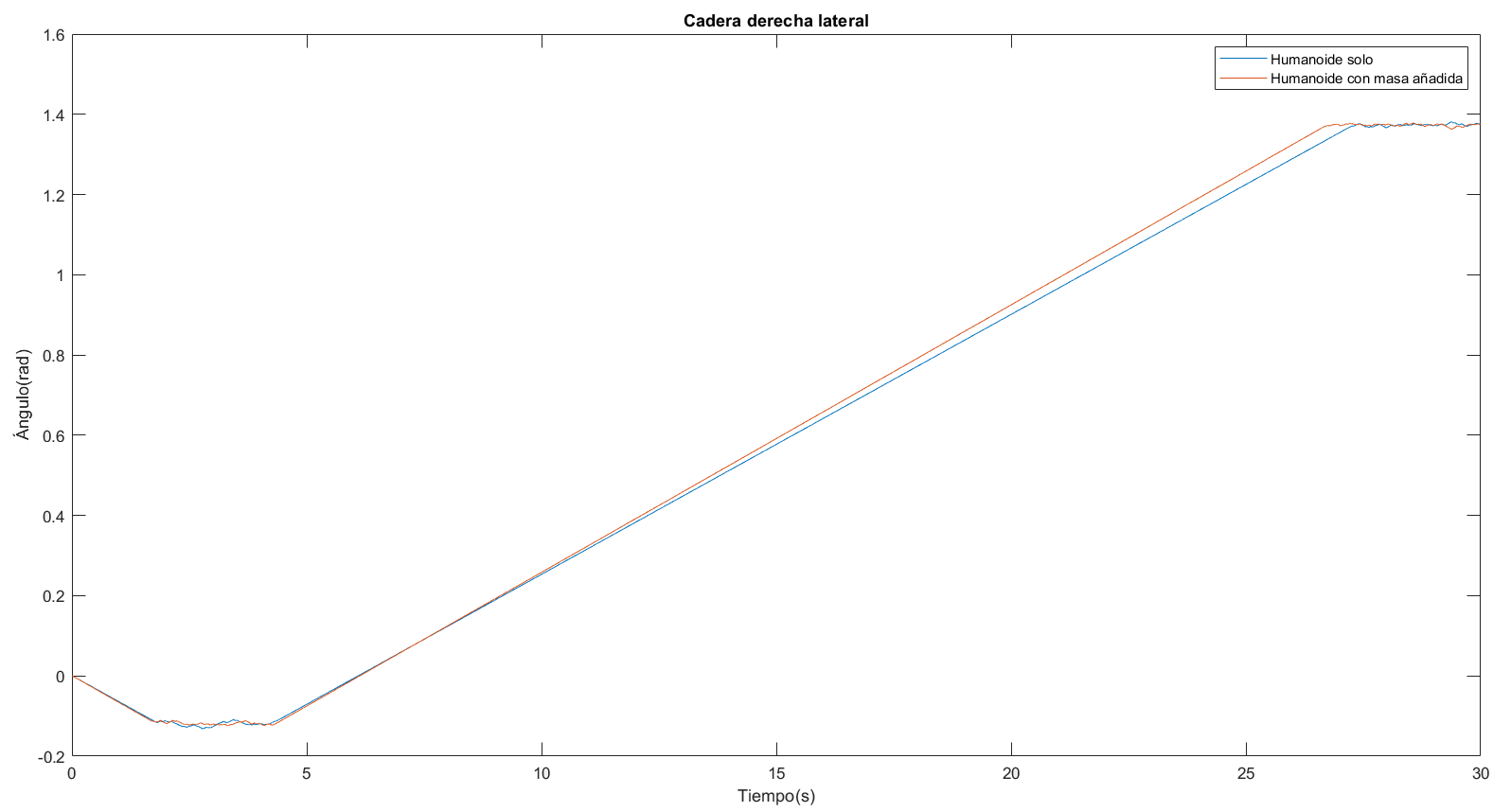

Supplement: Supplementary file 1 [file Data_Sheet_1.ZIP › figures/ComparisonArt11.png]

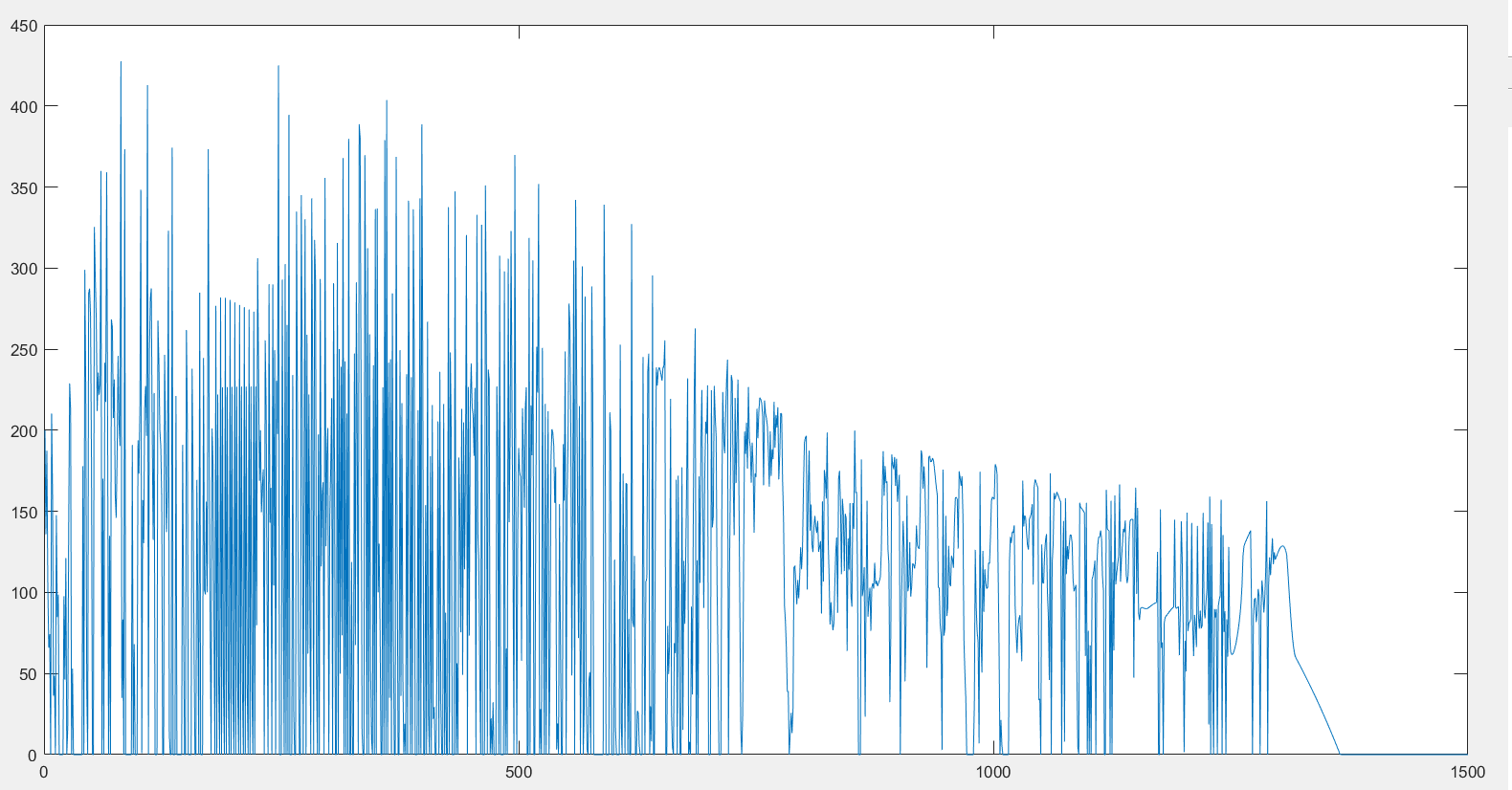

Supplement: Supplementary file 1 [file Data_Sheet_1.ZIP › figures/SignalWithoutFilter.png]

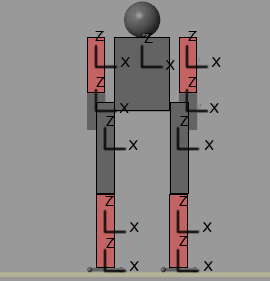

Supplement: Supplementary file 1 [file Data_Sheet_1.ZIP › figures/Position0.png]

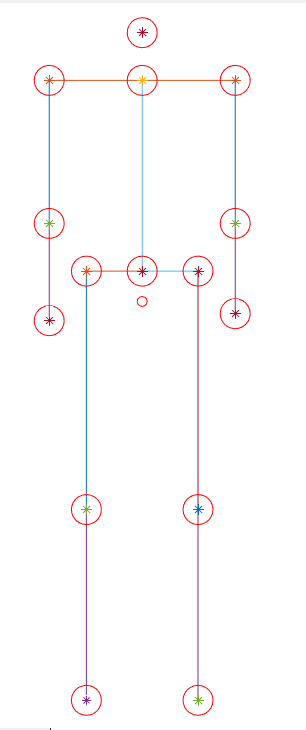

Supplement: Supplementary file 1 [file Data_Sheet_1.ZIP › figures/Dots.png]

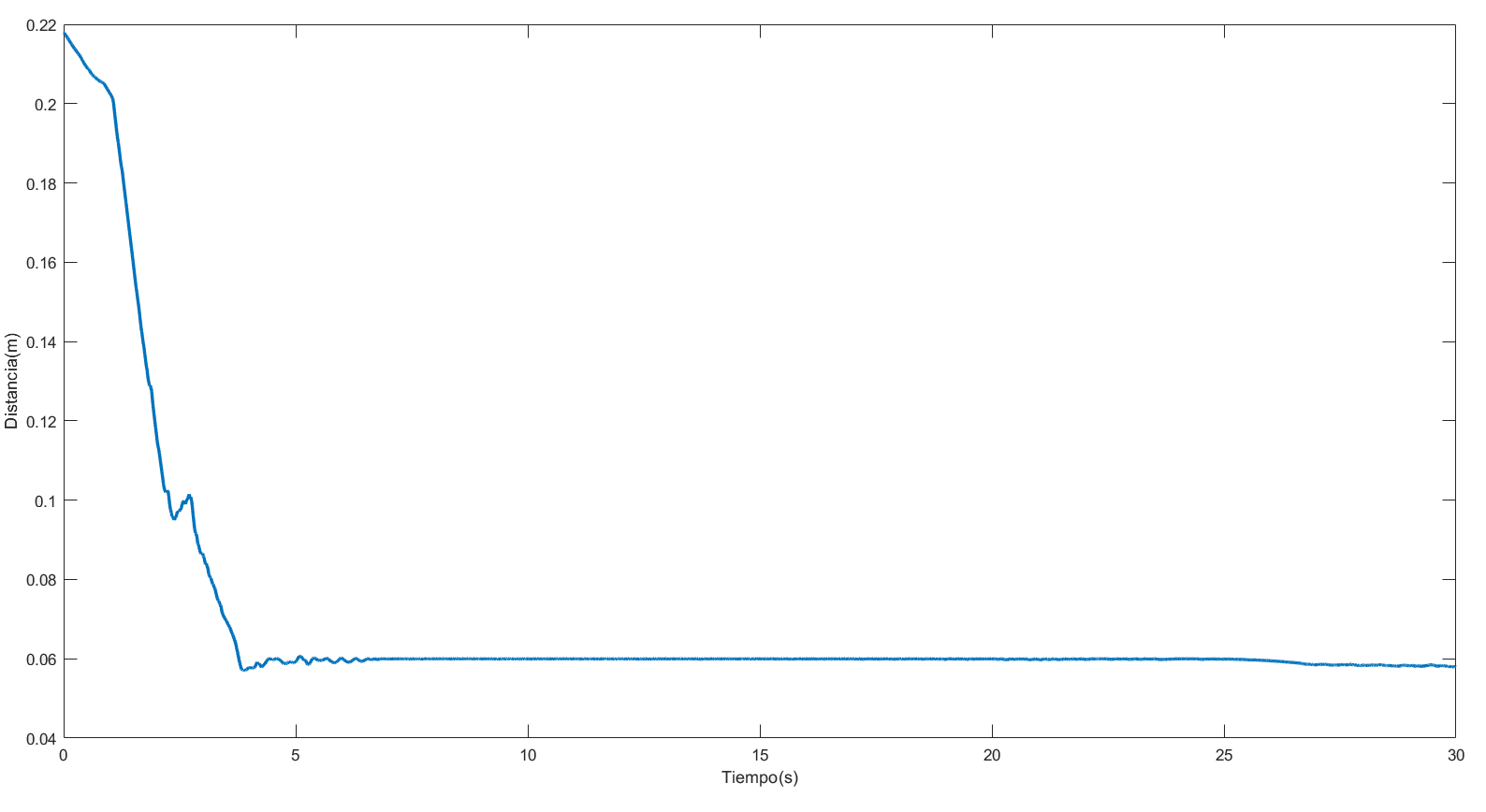

Supplement: Supplementary file 1 [file Data_Sheet_1.ZIP › figures/TrajectoryCMX4.png]

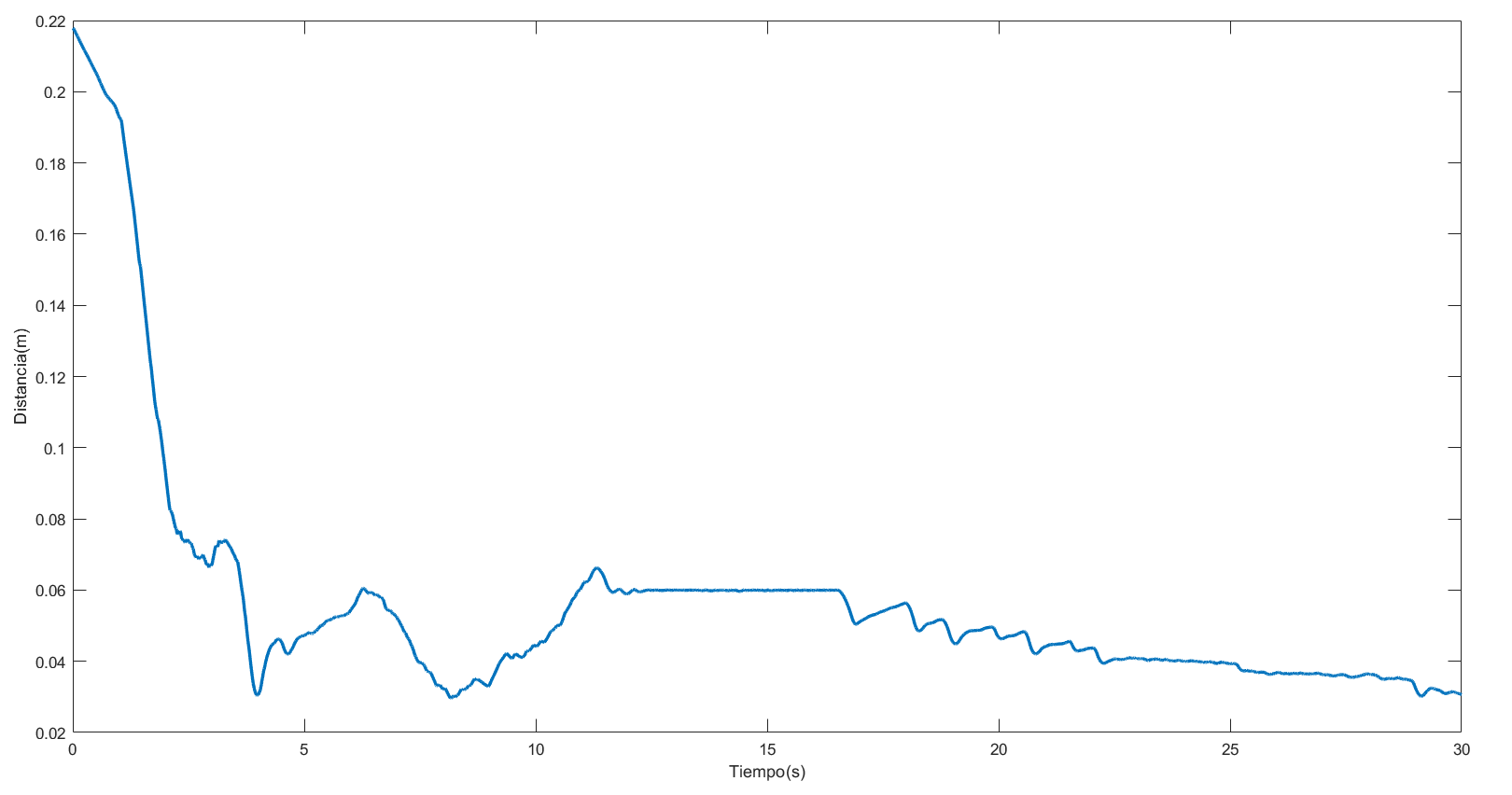

Supplement: Supplementary file 1 [file Data_Sheet_1.ZIP › figures/TrajectoryCMX5.png]

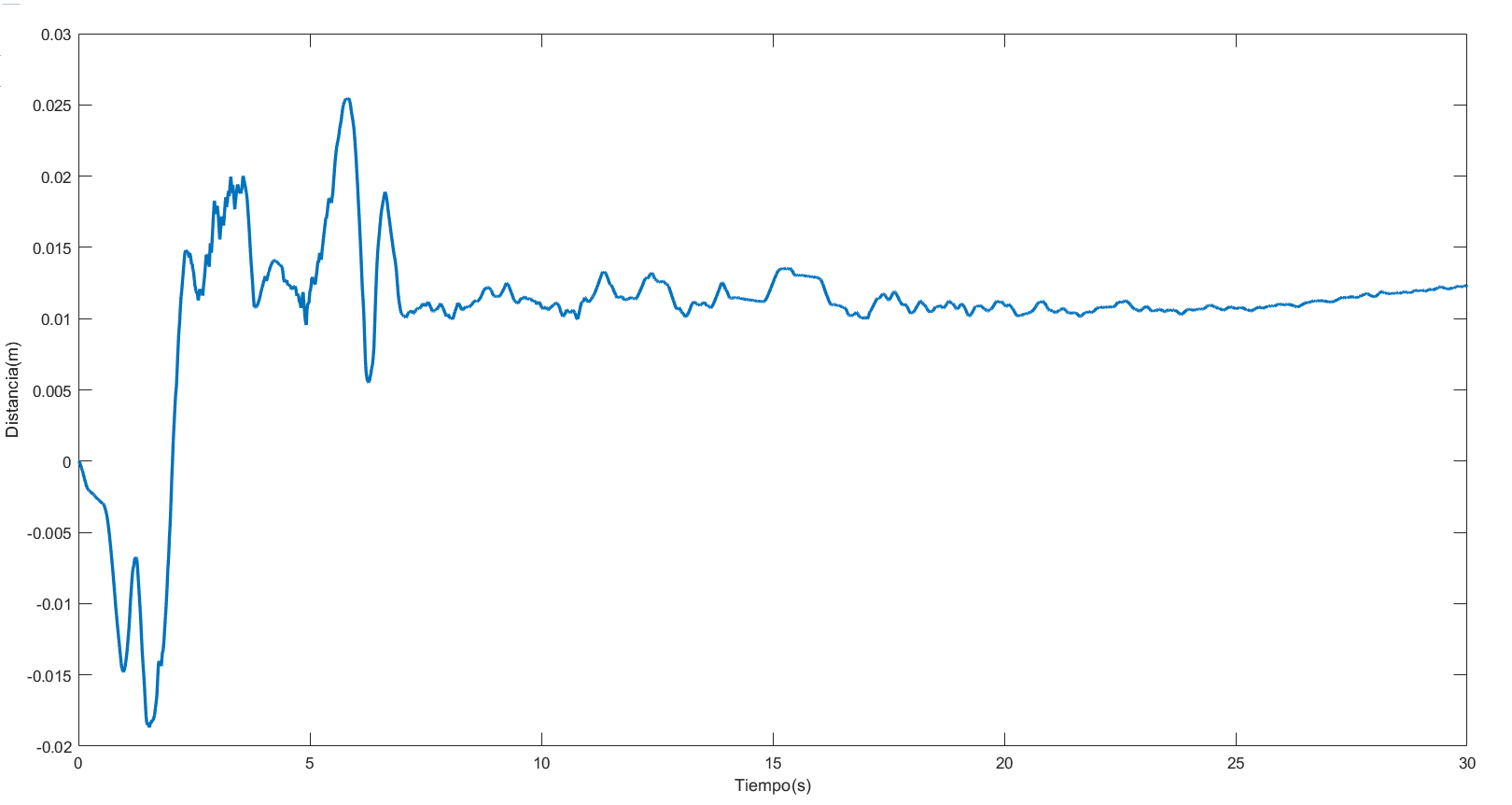

Supplement: Supplementary file 1 [file Data_Sheet_1.ZIP › figures/TrajectoryCMY1.png]

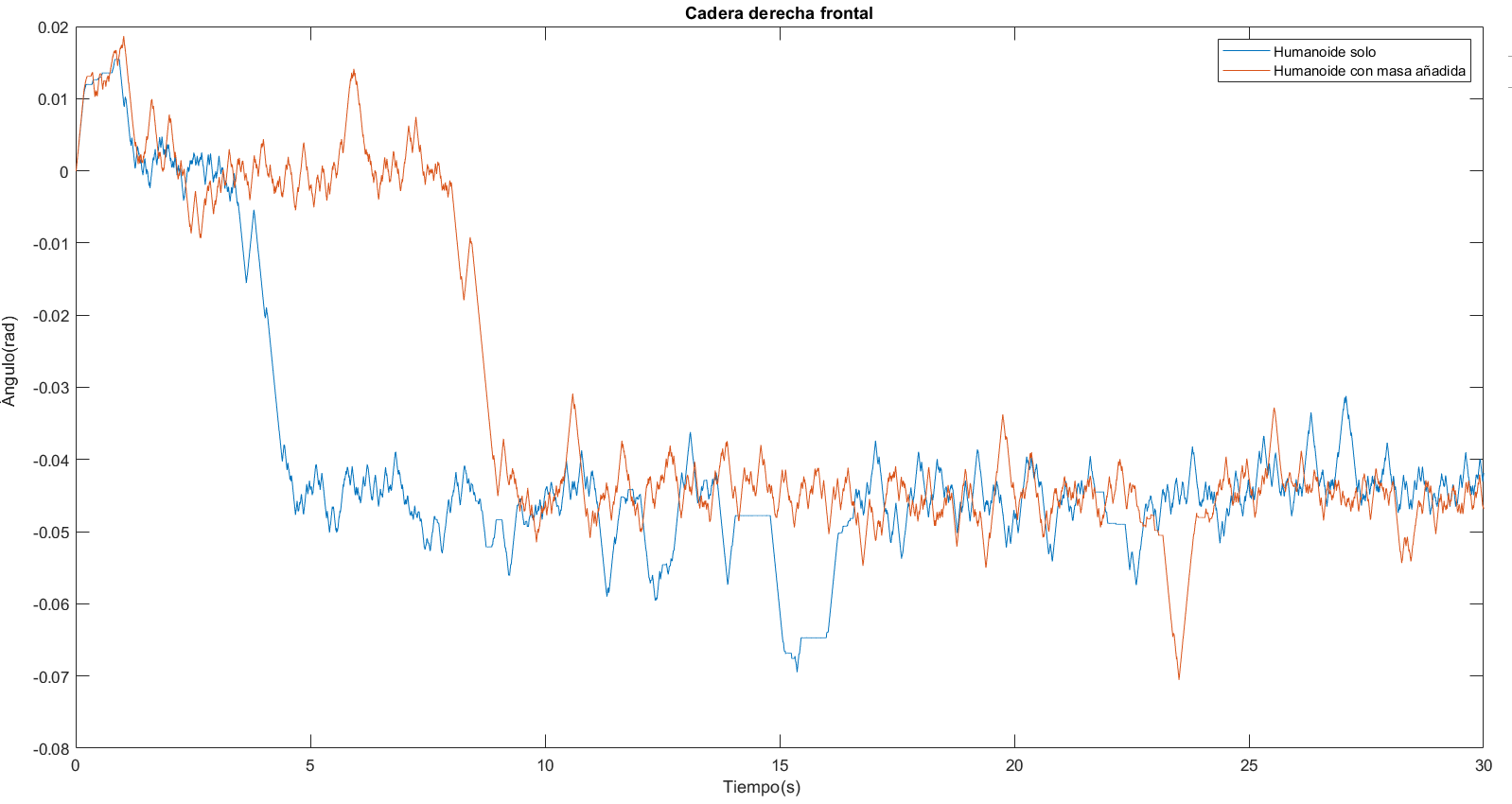

Supplement: Supplementary file 1 [file Data_Sheet_1.ZIP › figures/ComparisonArt12.png]

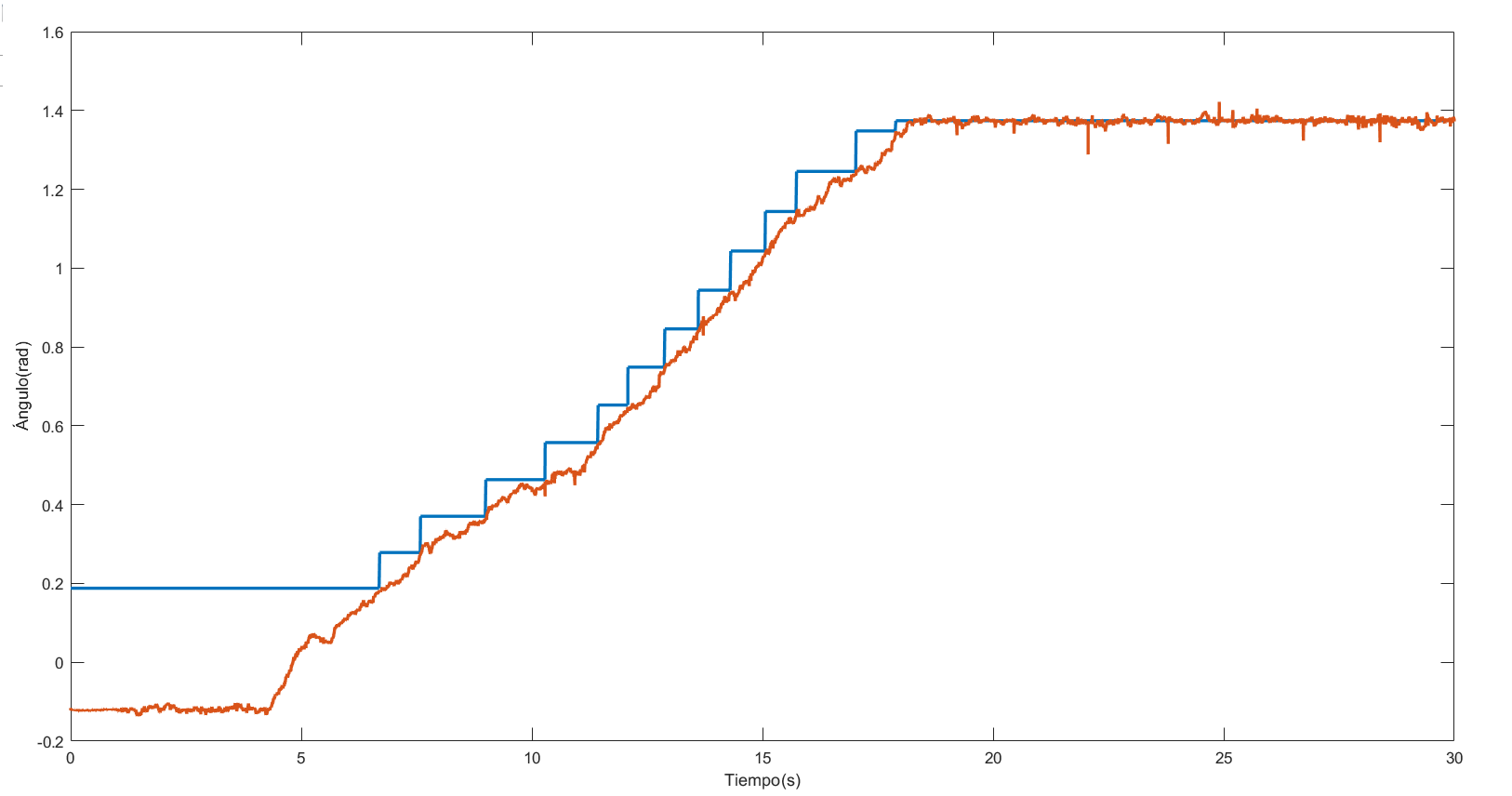

Supplement: Supplementary file 1 [file Data_Sheet_1.ZIP › figures/Learning4.png]

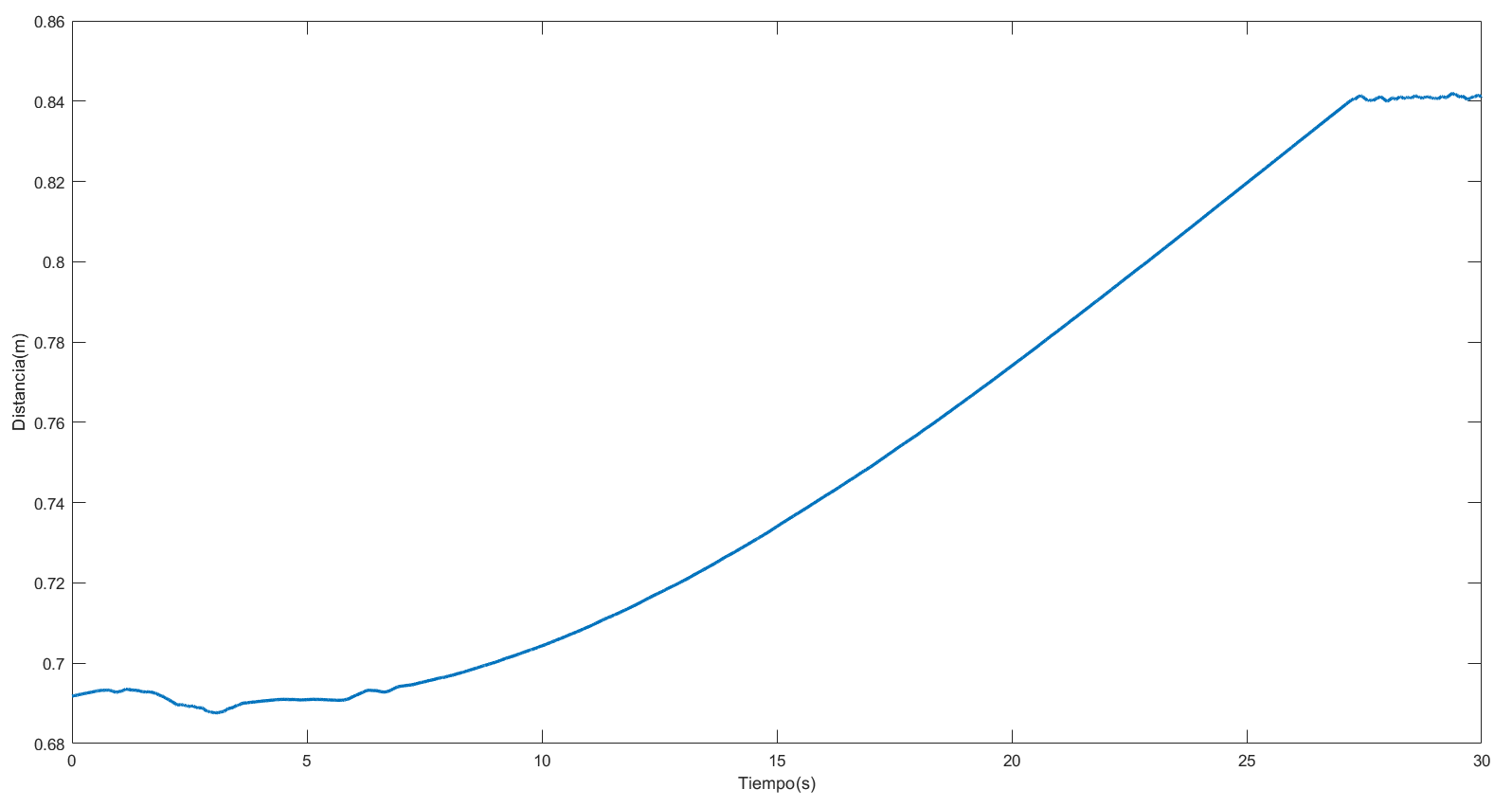

Supplement: Supplementary file 1 [file Data_Sheet_1.ZIP › figures/TrajectoryCMZ1.png]

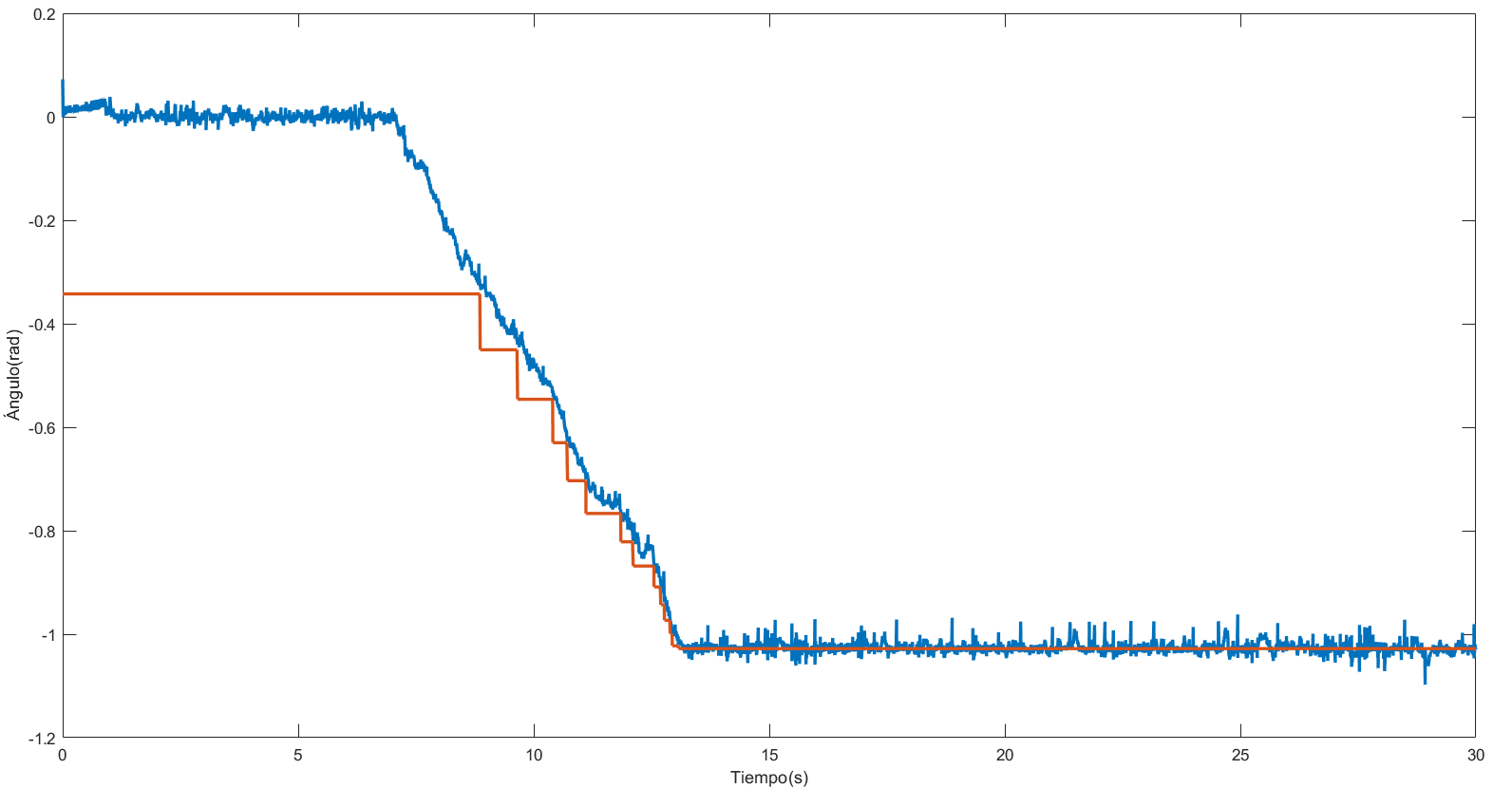

Supplement: Supplementary file 1 [file Data_Sheet_1.ZIP › figures/Learning5.png]

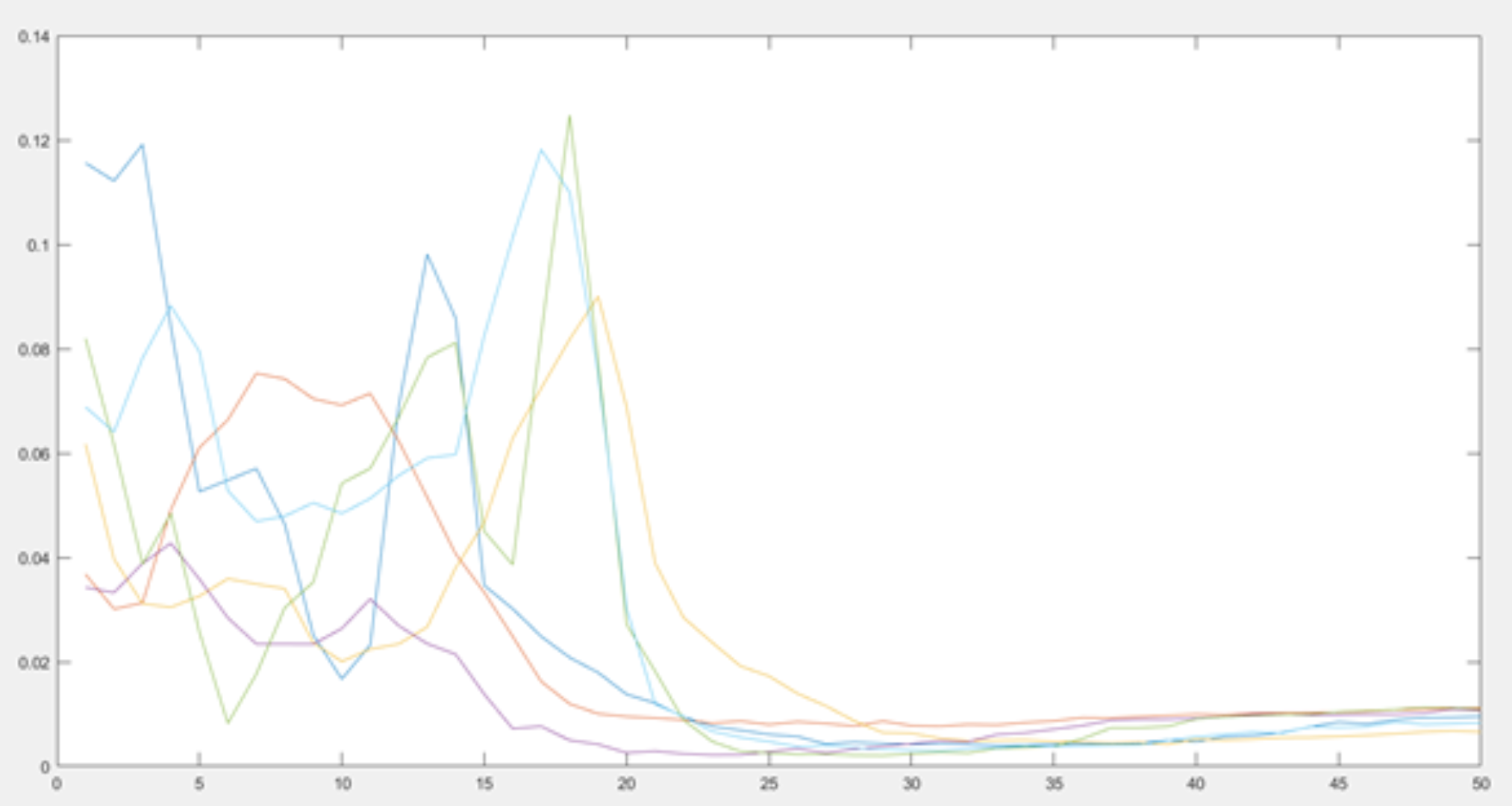

Supplement: Supplementary file 1 [file Data_Sheet_1.ZIP › figures/WithAnnealing.png]

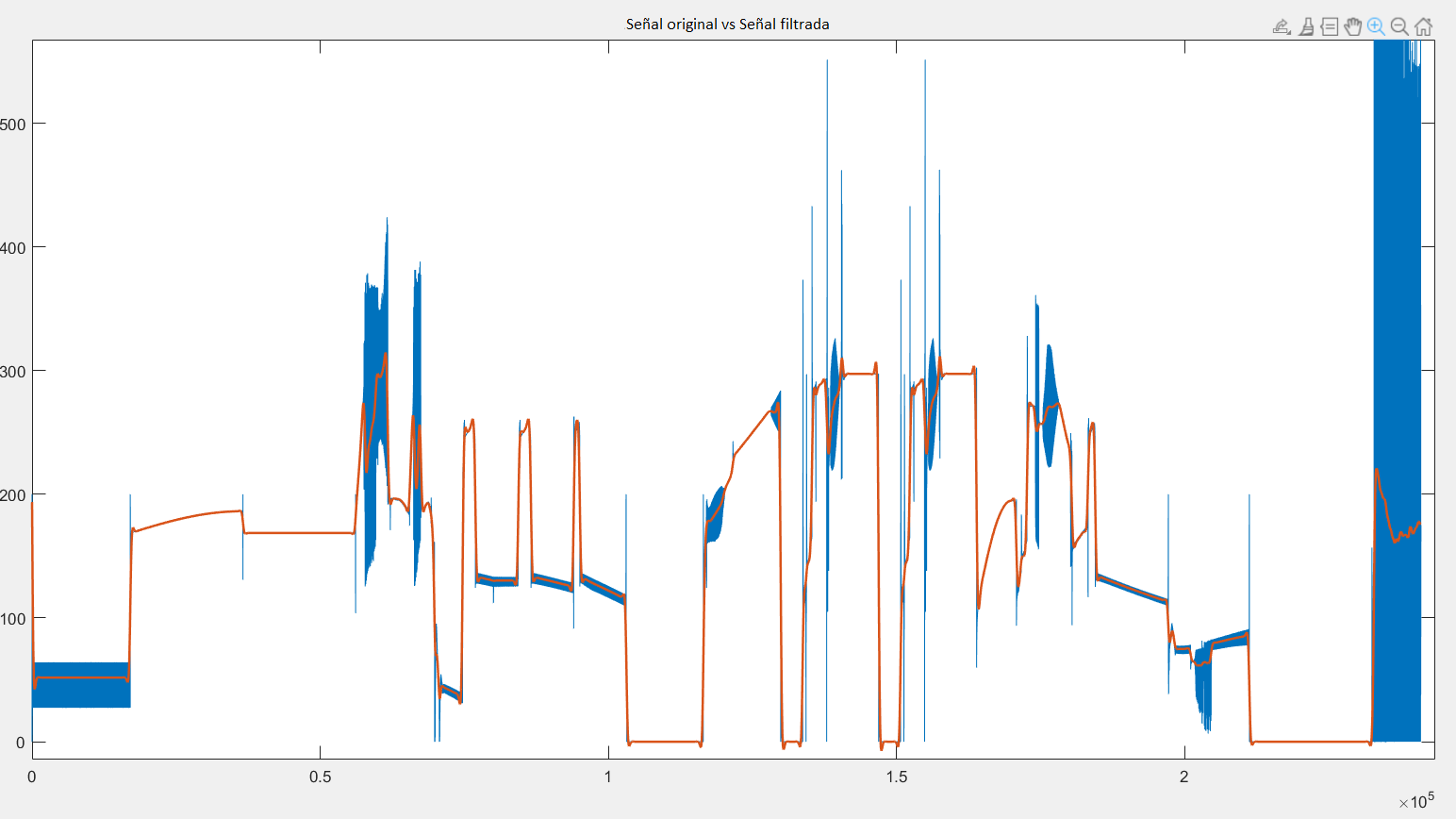

Supplement: Supplementary file 1 [file Data_Sheet_1.ZIP › figures/SenΓòáa╠éaloriginalyfiltrada.png]

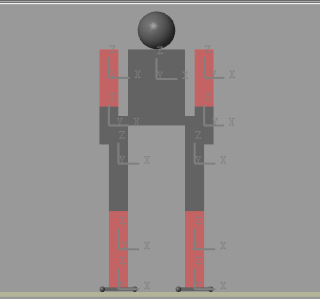

Supplement: Supplementary file 1 [file Data_Sheet_1.ZIP › figures/bipedo1.png]

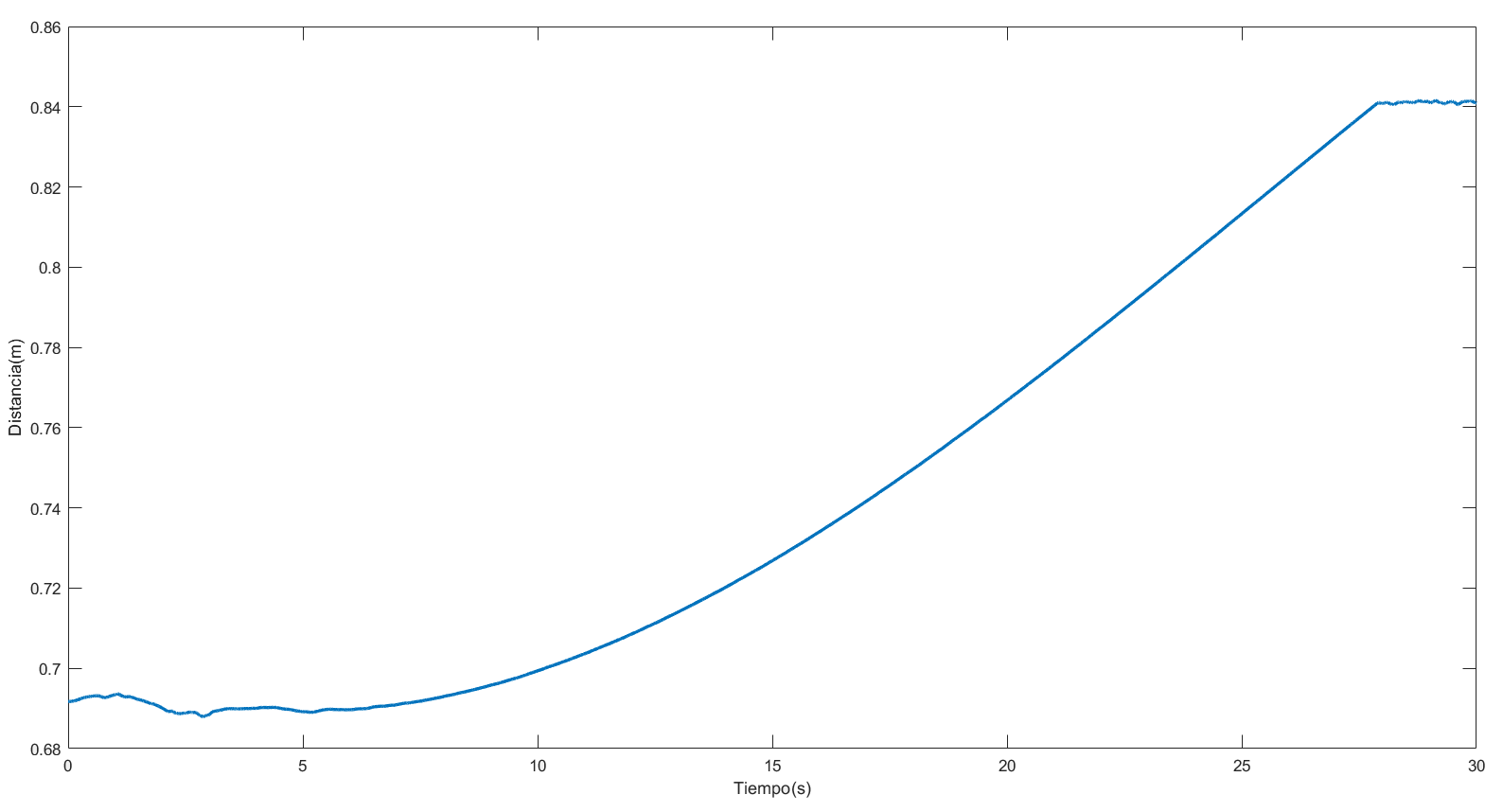

Supplement: Supplementary file 1 [file Data_Sheet_1.ZIP › figures/TrajectoryCMZ3.png]

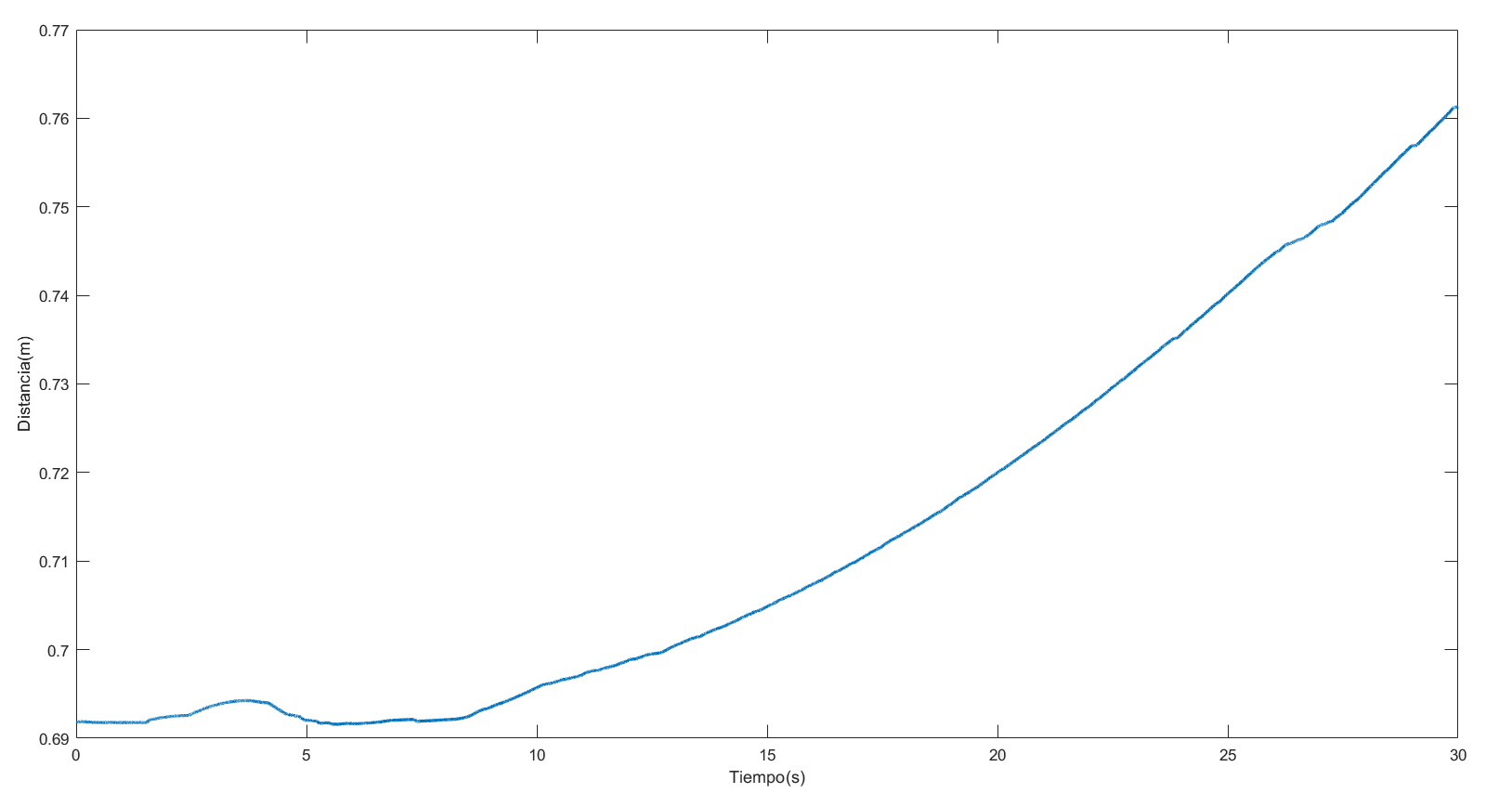

Supplement: Supplementary file 1 [file Data_Sheet_1.ZIP › figures/TrajectoryCMZ2.png]

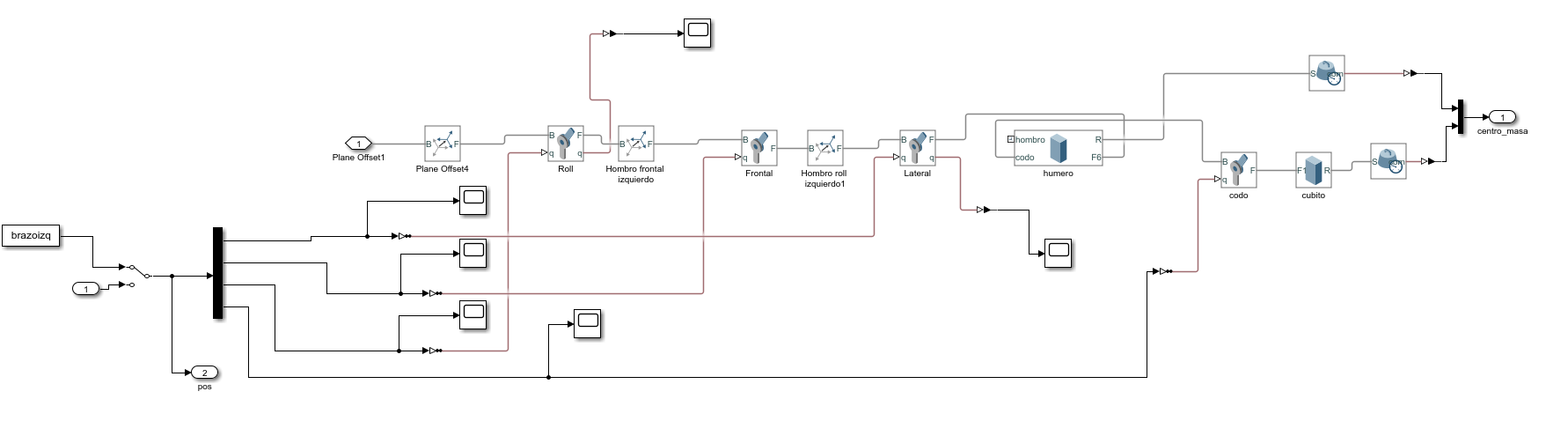

Supplement: Supplementary file 1 [file Data_Sheet_1.ZIP › figures/ArmBlocks.png]

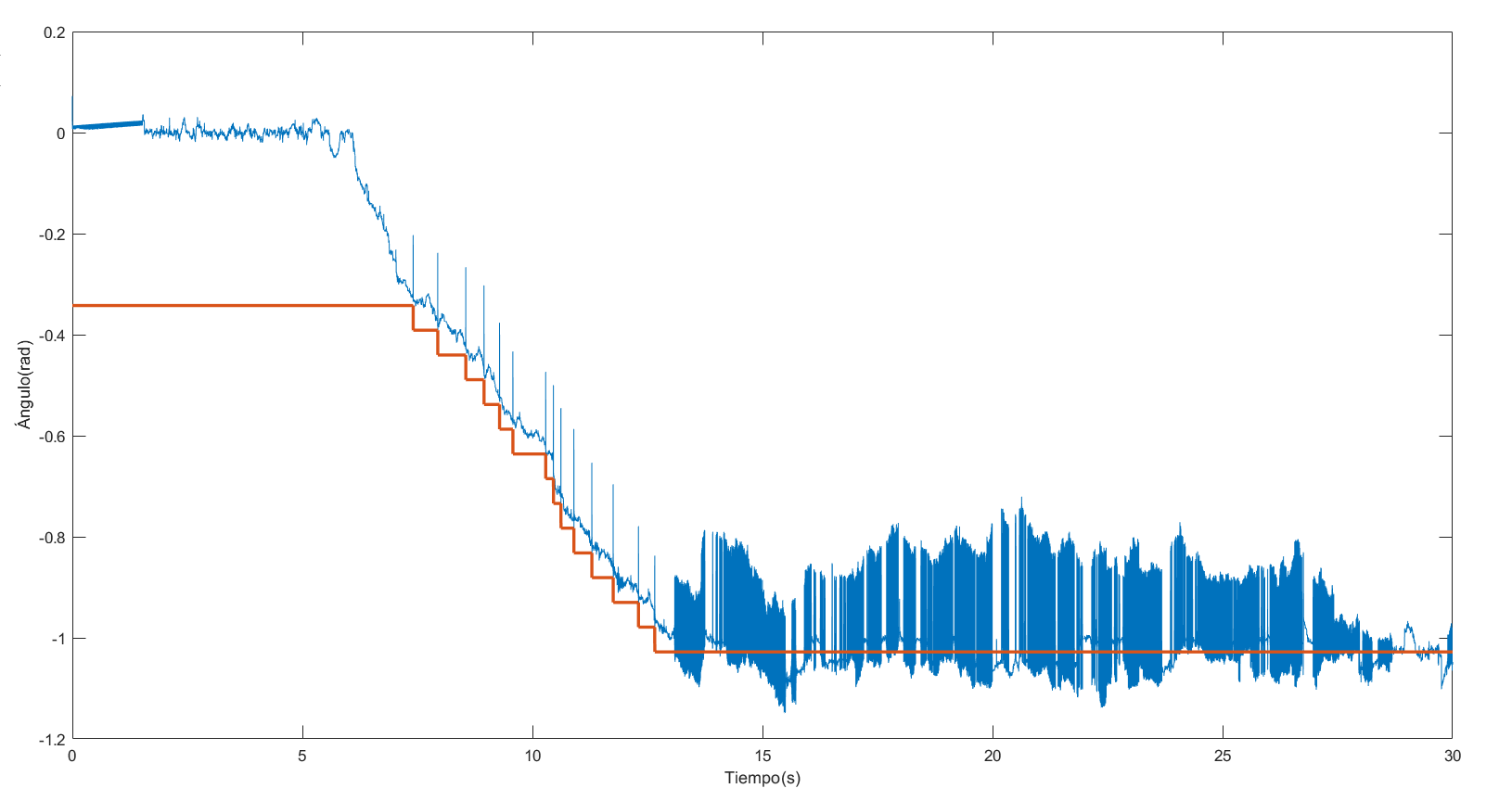

Supplement: Supplementary file 1 [file Data_Sheet_1.ZIP › figures/Learning2.png]

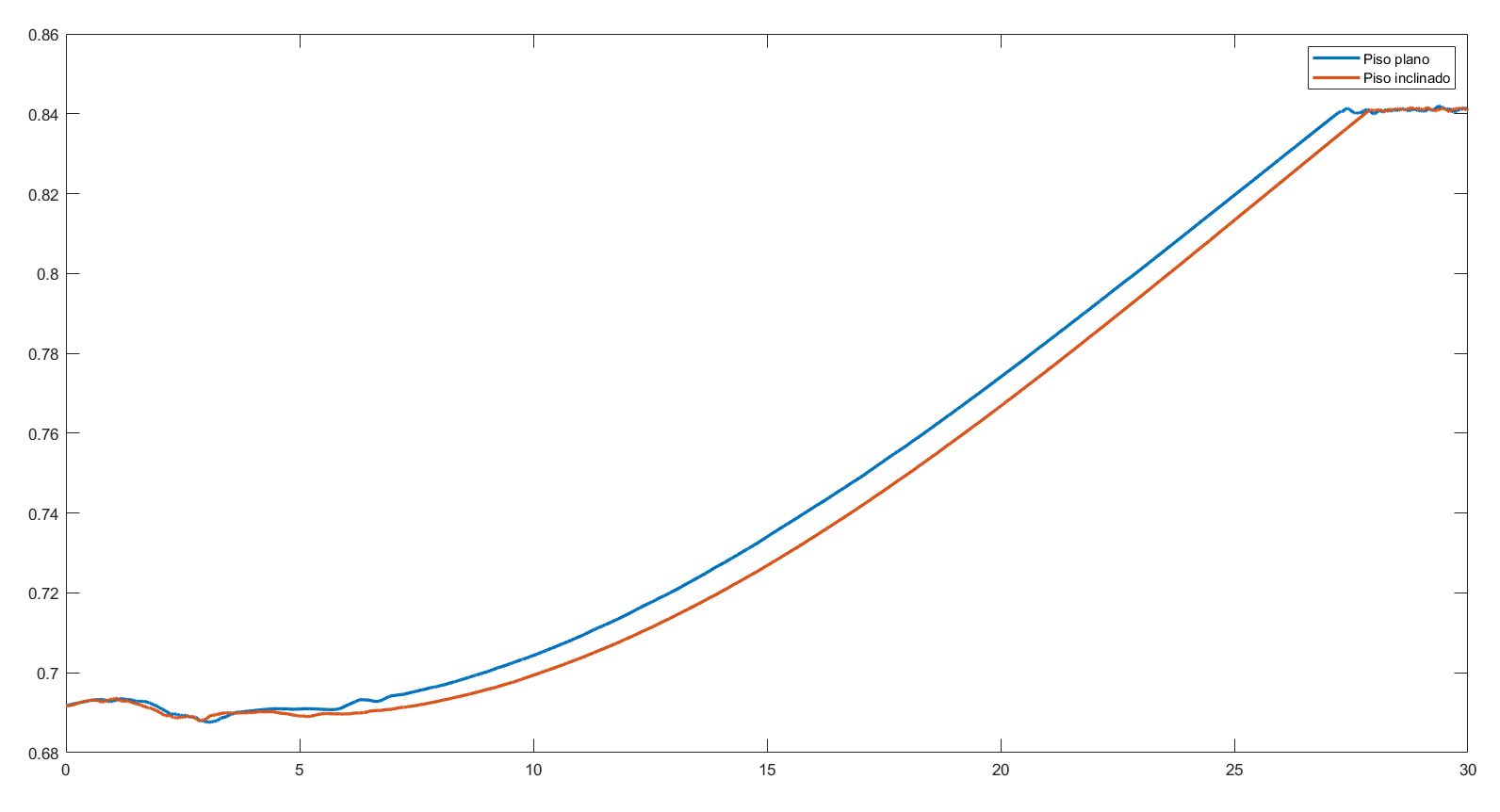

Supplement: Supplementary file 1 [file Data_Sheet_1.ZIP › figures/CompracionCMZ1.png]

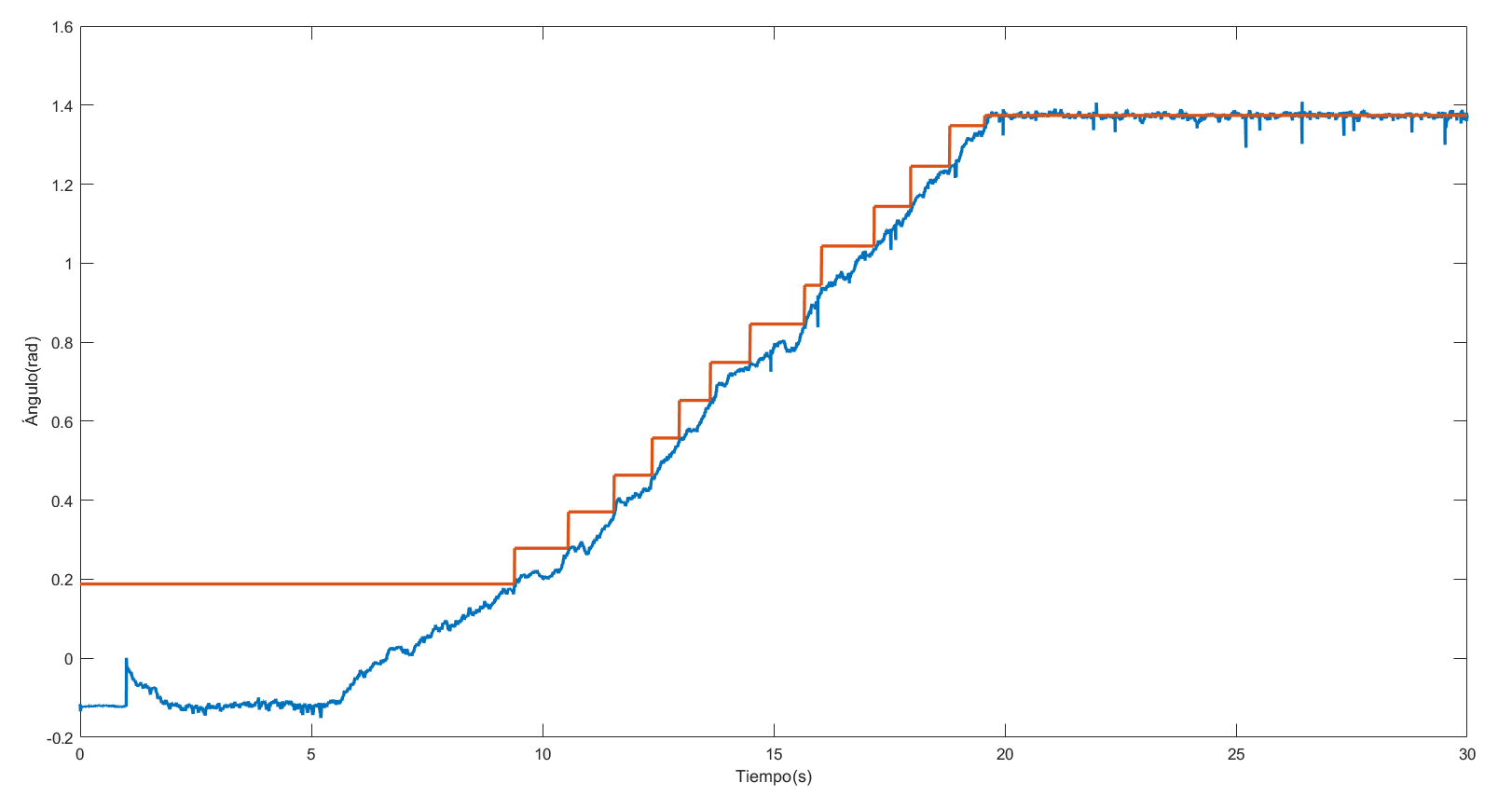

Supplement: Supplementary file 1 [file Data_Sheet_1.ZIP › figures/Learning3.png]

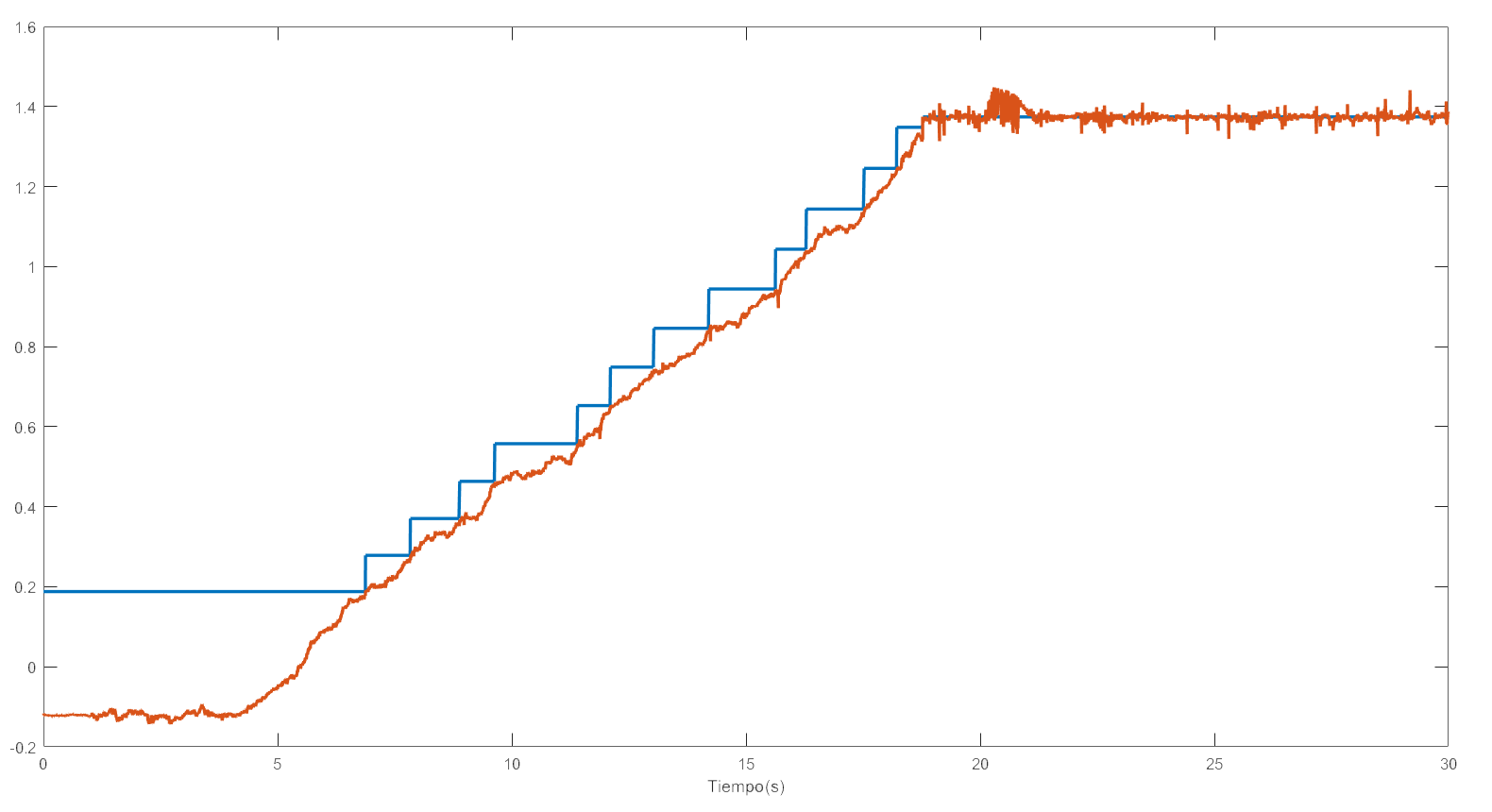

Supplement: Supplementary file 1 [file Data_Sheet_1.ZIP › figures/Learning1.png]

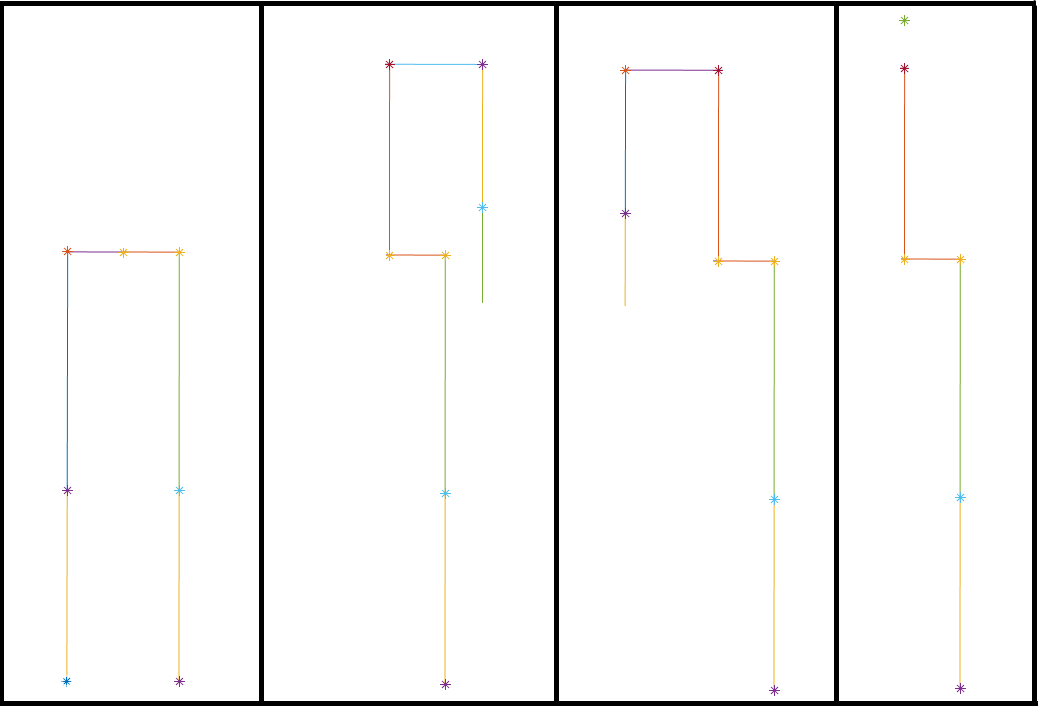

Supplement: Supplementary file 1 [file Data_Sheet_1.ZIP › figures/CD1 copy.png]

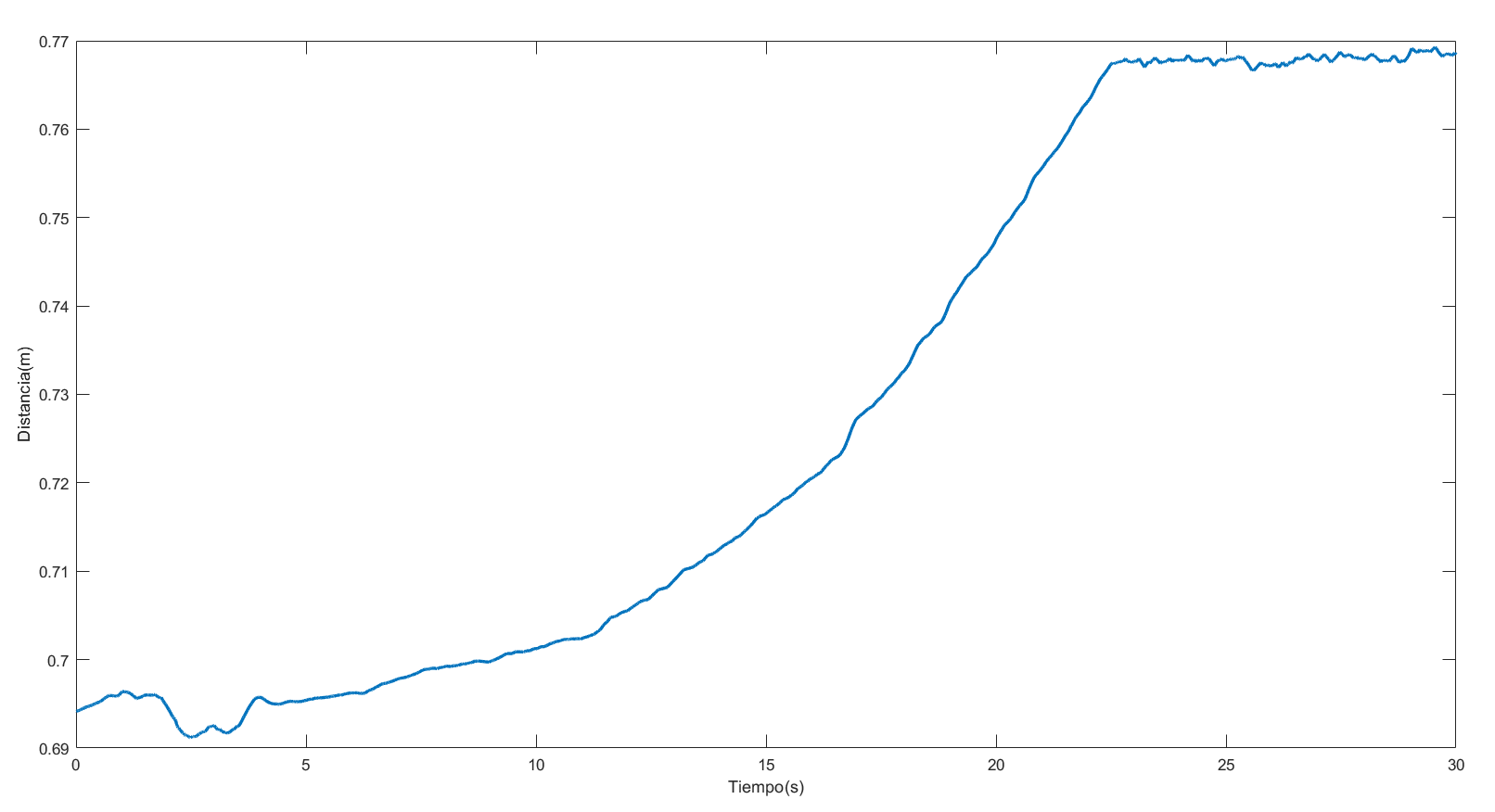

Supplement: Supplementary file 1 [file Data_Sheet_1.ZIP › figures/TrajectoryCMZ5.png]

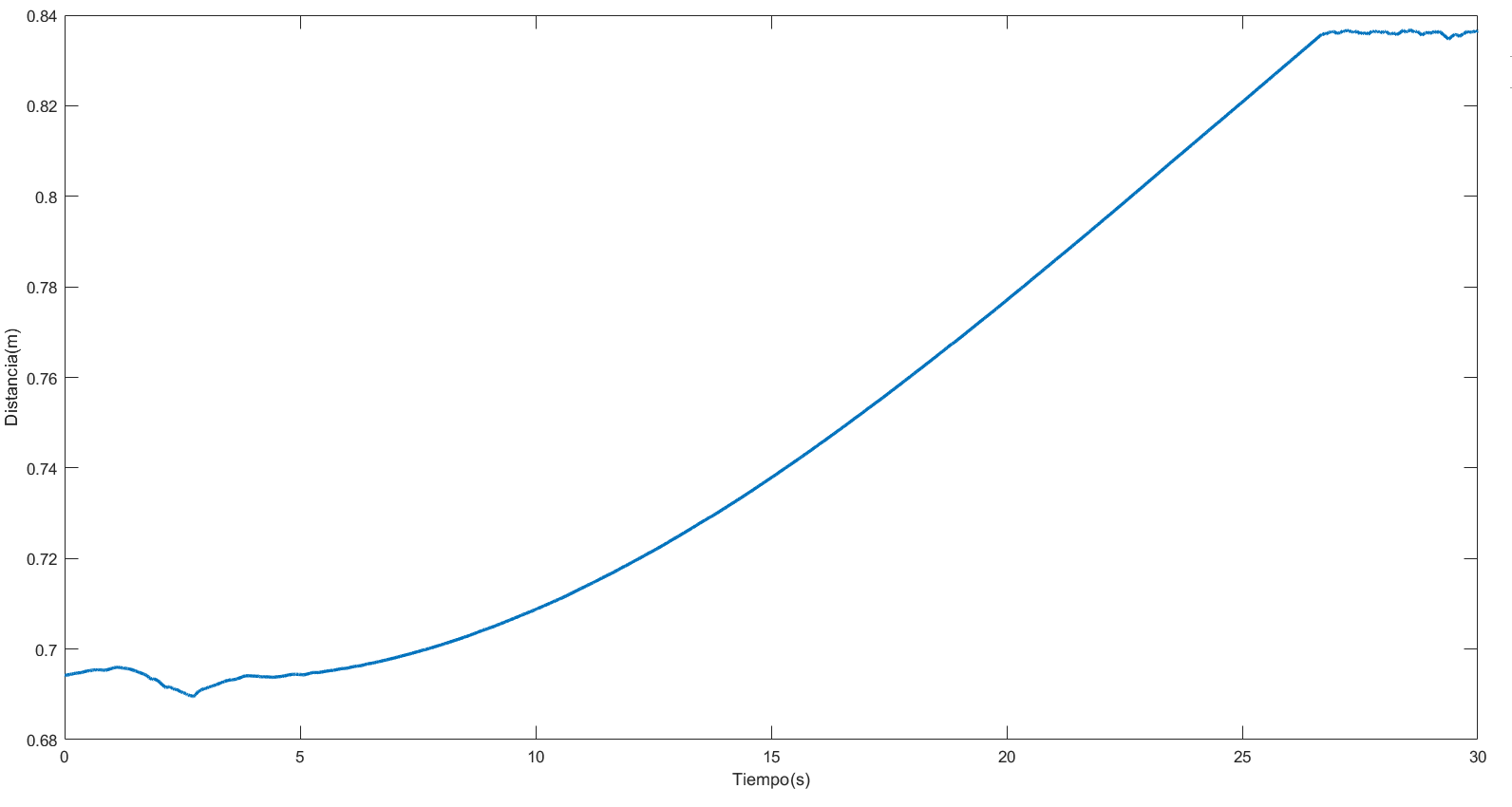

Supplement: Supplementary file 1 [file Data_Sheet_1.ZIP › figures/TrajectoryCMZ4.png]

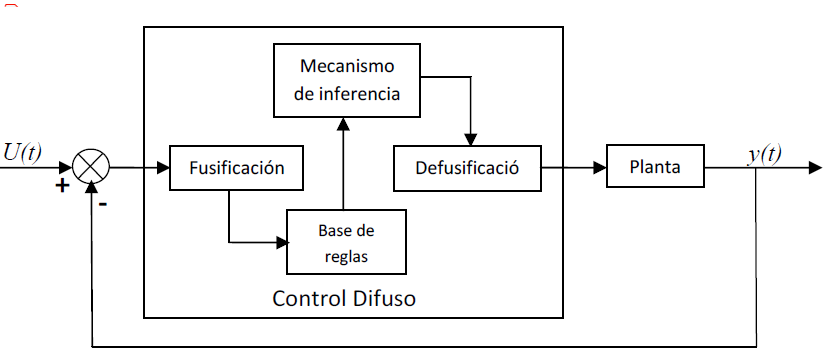

Supplement: Supplementary file 1 [file Data_Sheet_1.ZIP › figures/DiagramaControlDifuso.png]

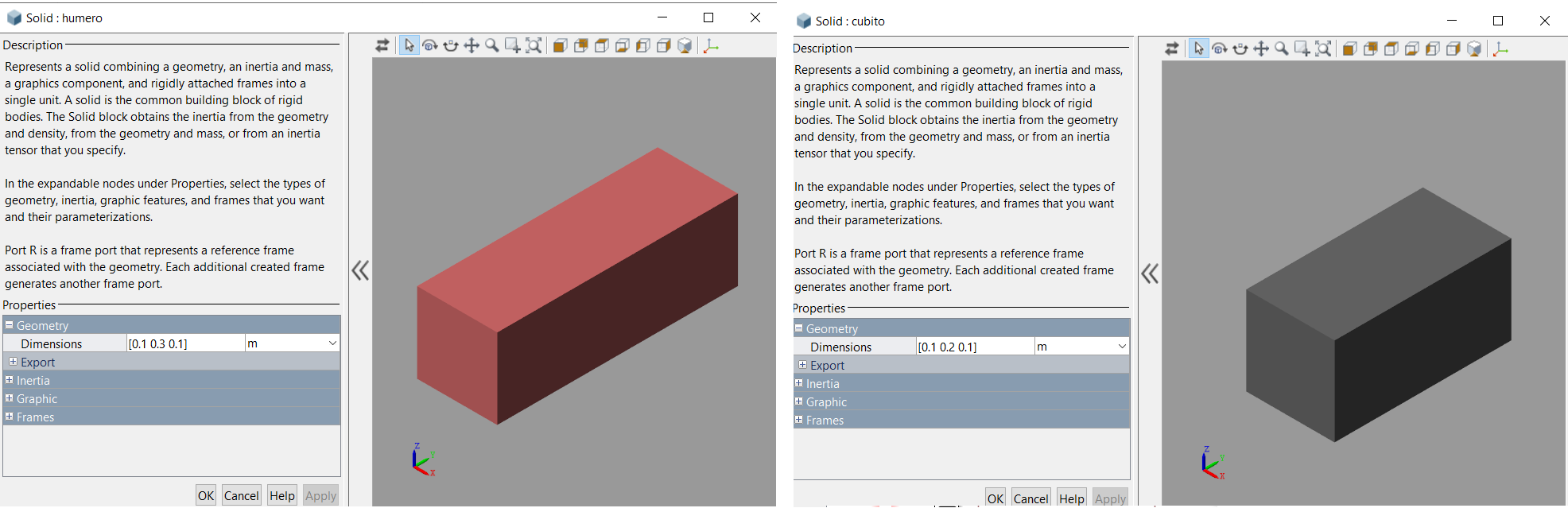

Supplement: Supplementary file 1 [file Data_Sheet_1.ZIP › figures/SimulinkHumer.png]

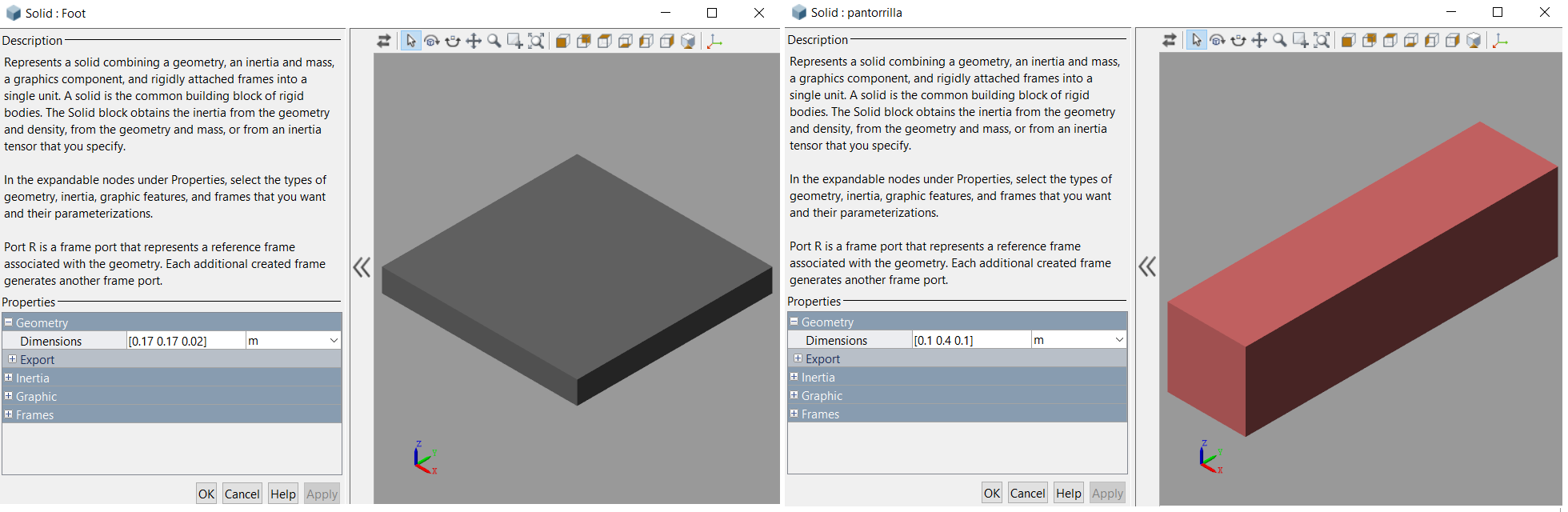

Supplement: Supplementary file 1 [file Data_Sheet_1.ZIP › figures/SimulinkPie.png]

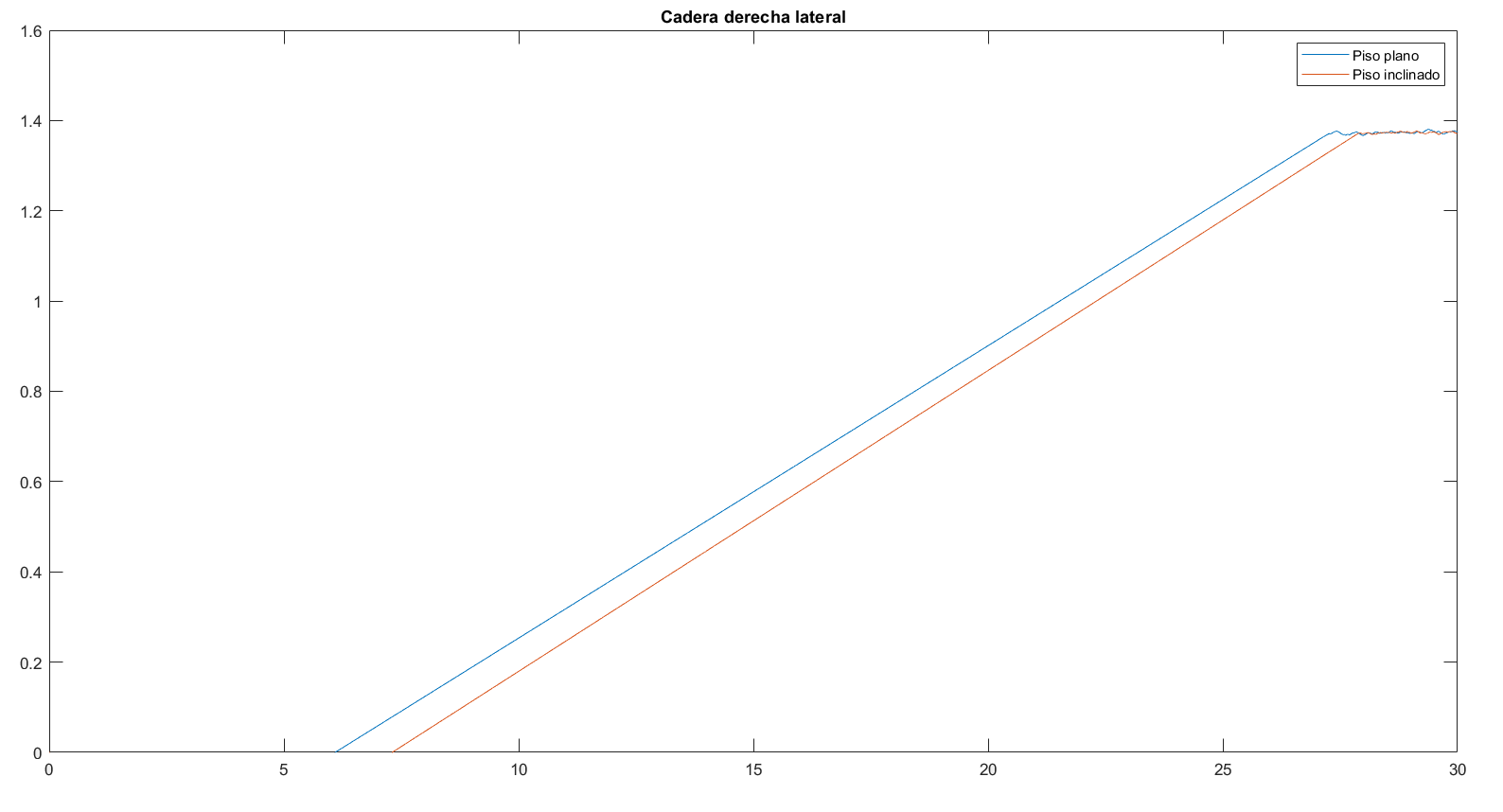

Supplement: Supplementary file 1 [file Data_Sheet_1.ZIP › figures/Comparison1Art5.png]

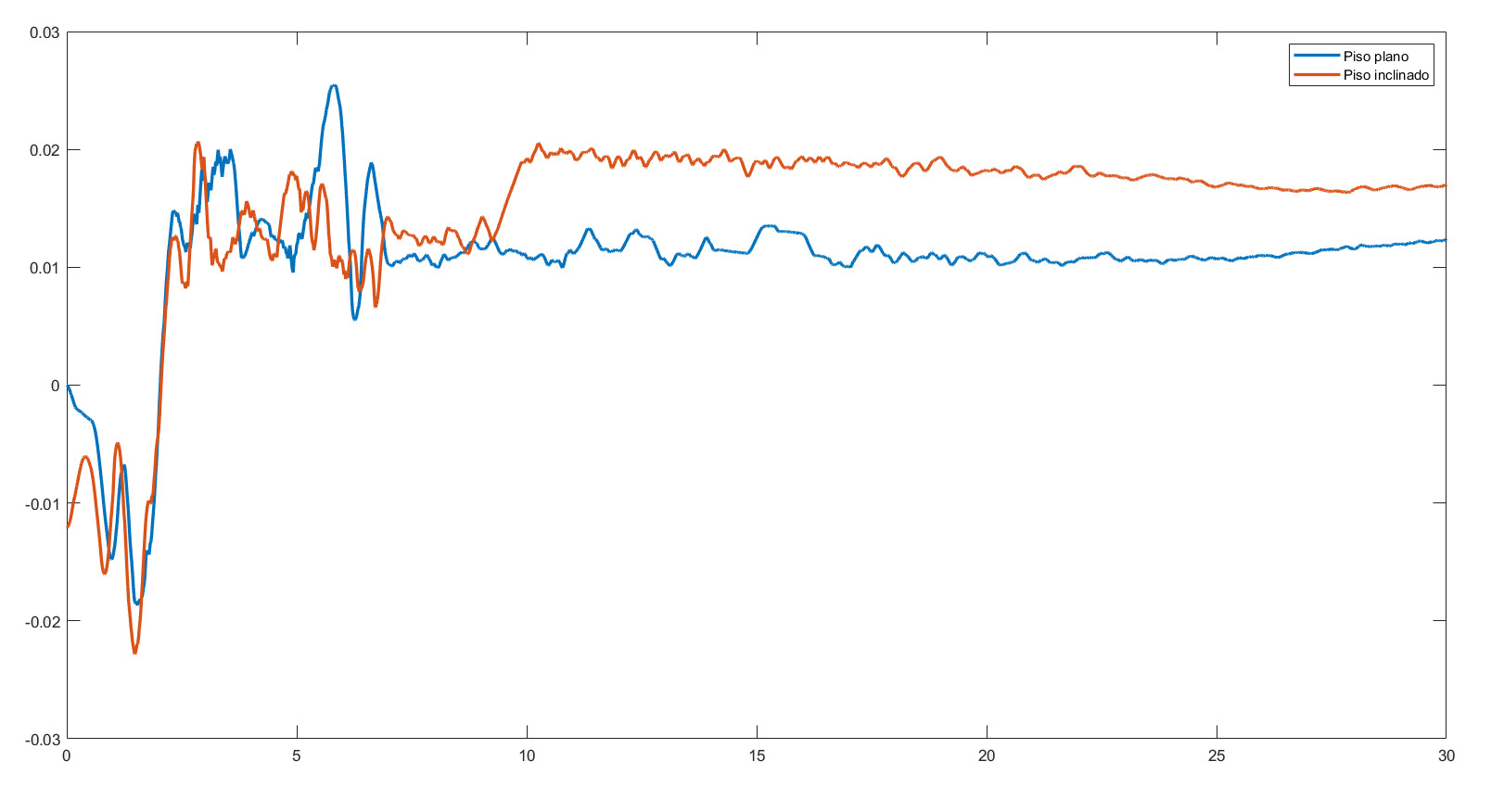

Supplement: Supplementary file 1 [file Data_Sheet_1.ZIP › figures/ComparisonCMY1.png]

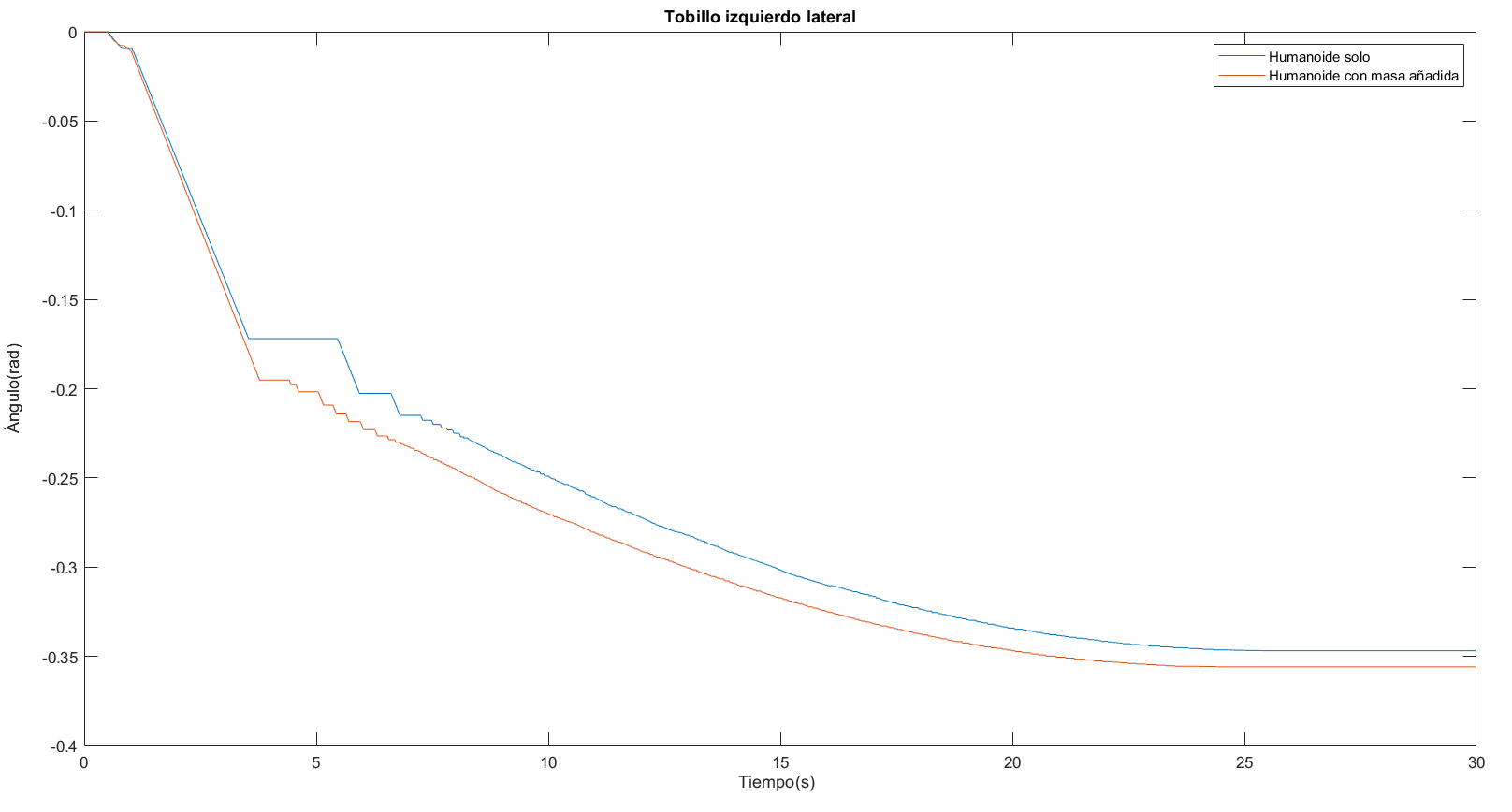

Supplement: Supplementary file 1 [file Data_Sheet_1.ZIP › figures/ComparisonArt7.png]

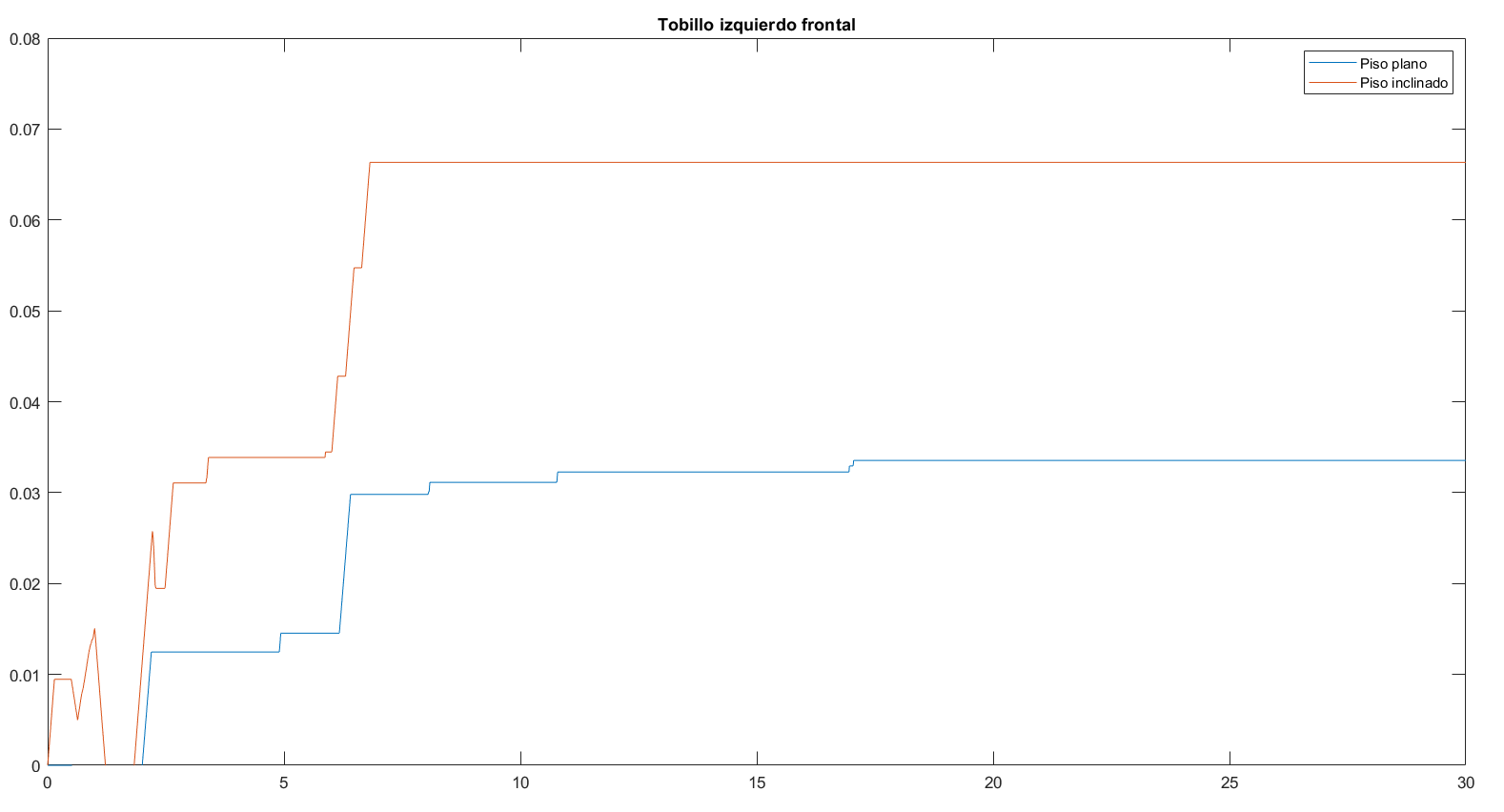

Supplement: Supplementary file 1 [file Data_Sheet_1.ZIP › figures/Comparison1Art4.png]

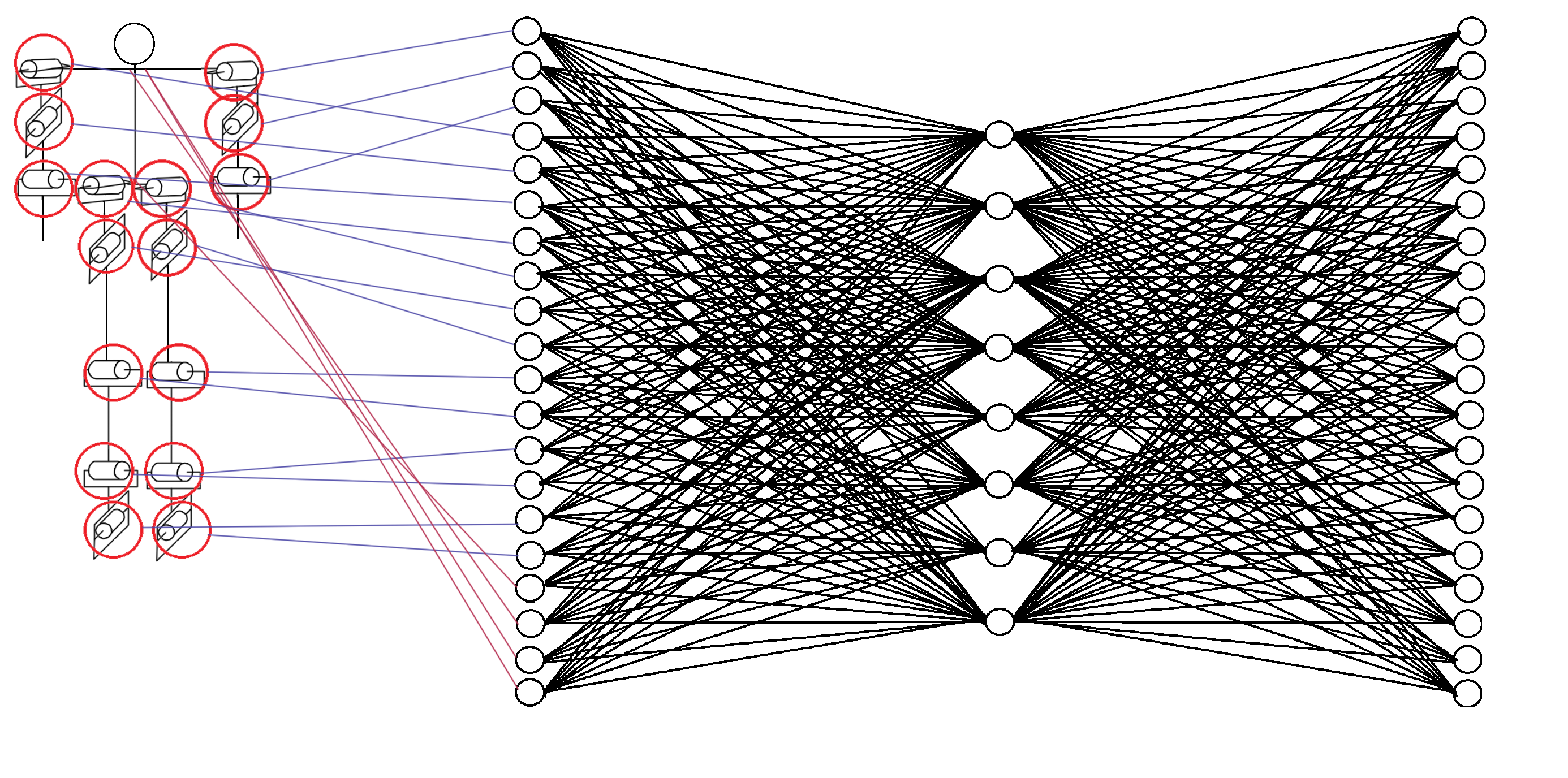

Supplement: Supplementary file 1 [file Data_Sheet_1.ZIP › figures/FullModel4.png]

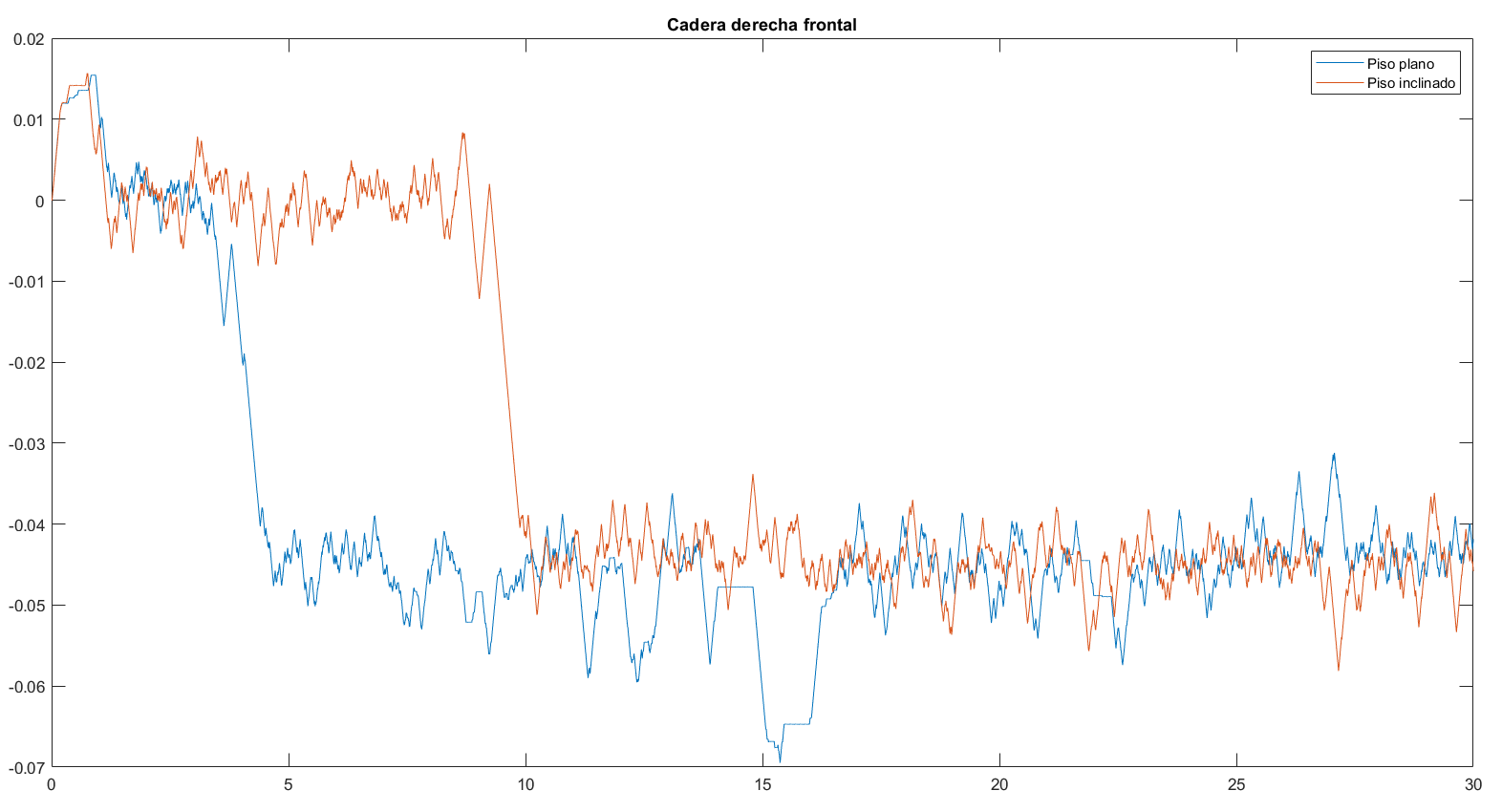

Supplement: Supplementary file 1 [file Data_Sheet_1.ZIP › figures/Comparison1Art6.png]

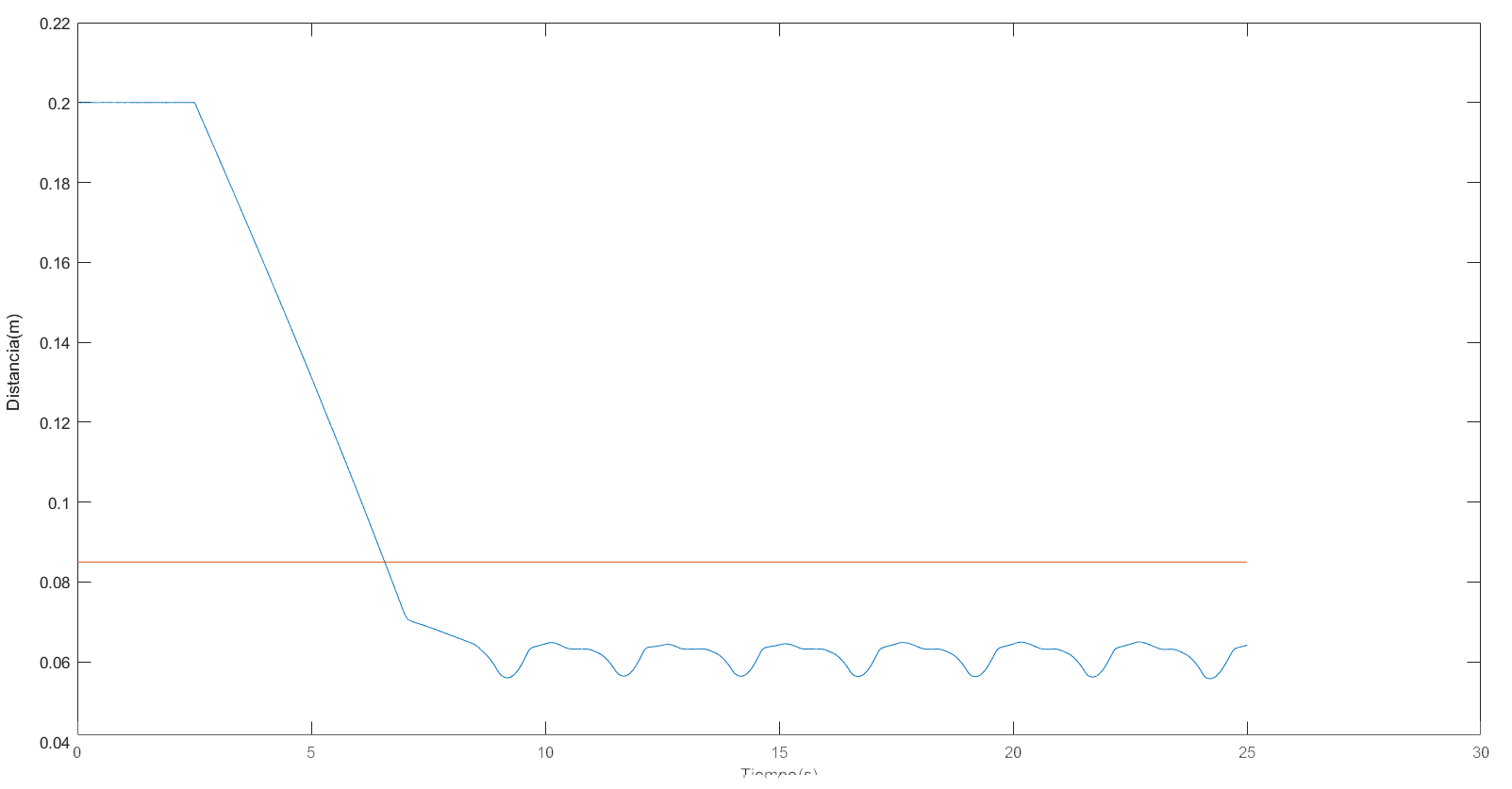

Supplement: Supplementary file 1 [file Data_Sheet_1.ZIP › figures/Trajectory1_CM.png]

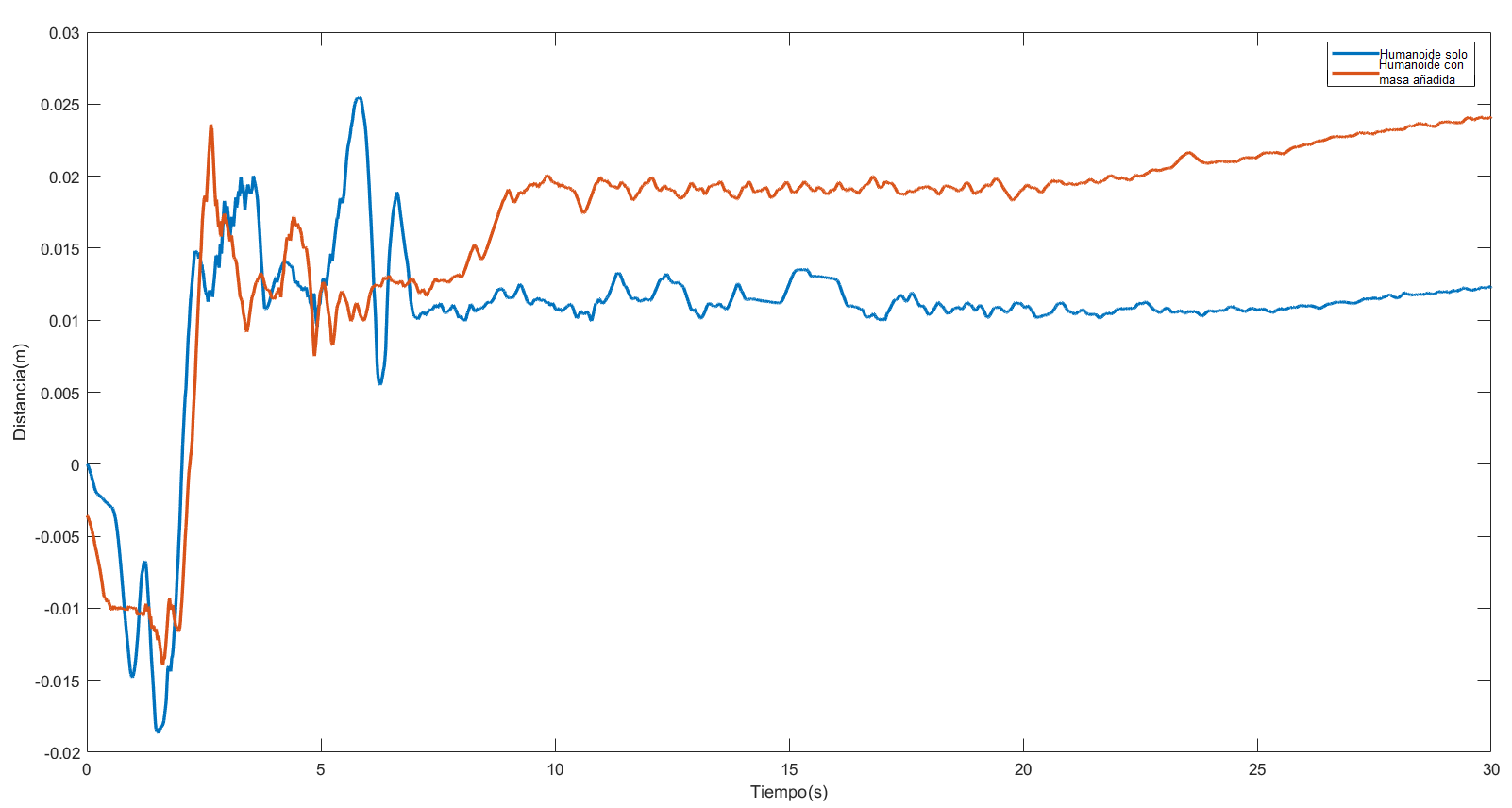

Supplement: Supplementary file 1 [file Data_Sheet_1.ZIP › figures/ComparisonCMY2.png]

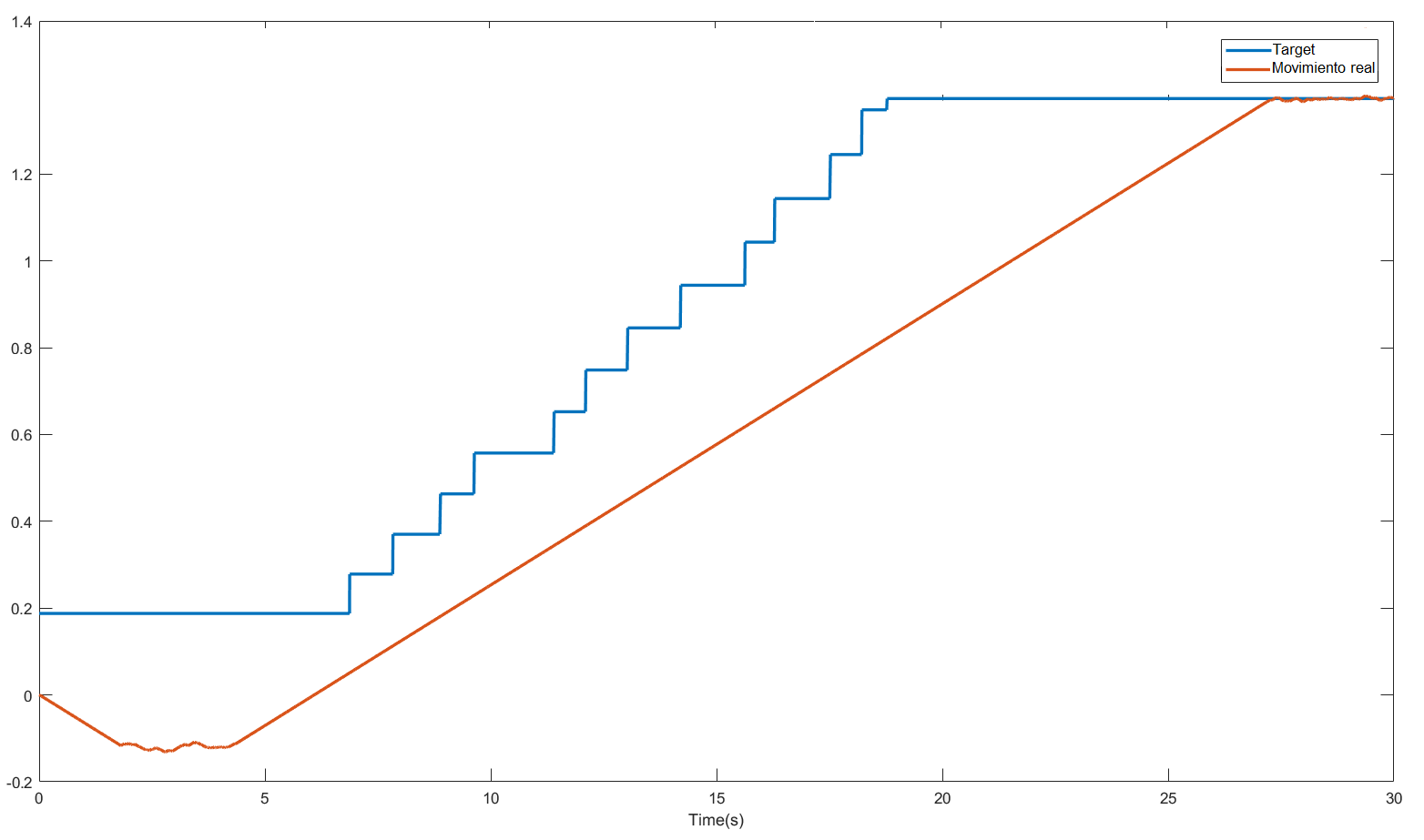

Supplement: Supplementary file 1 [file Data_Sheet_1.ZIP › figures/MovementPath.png]

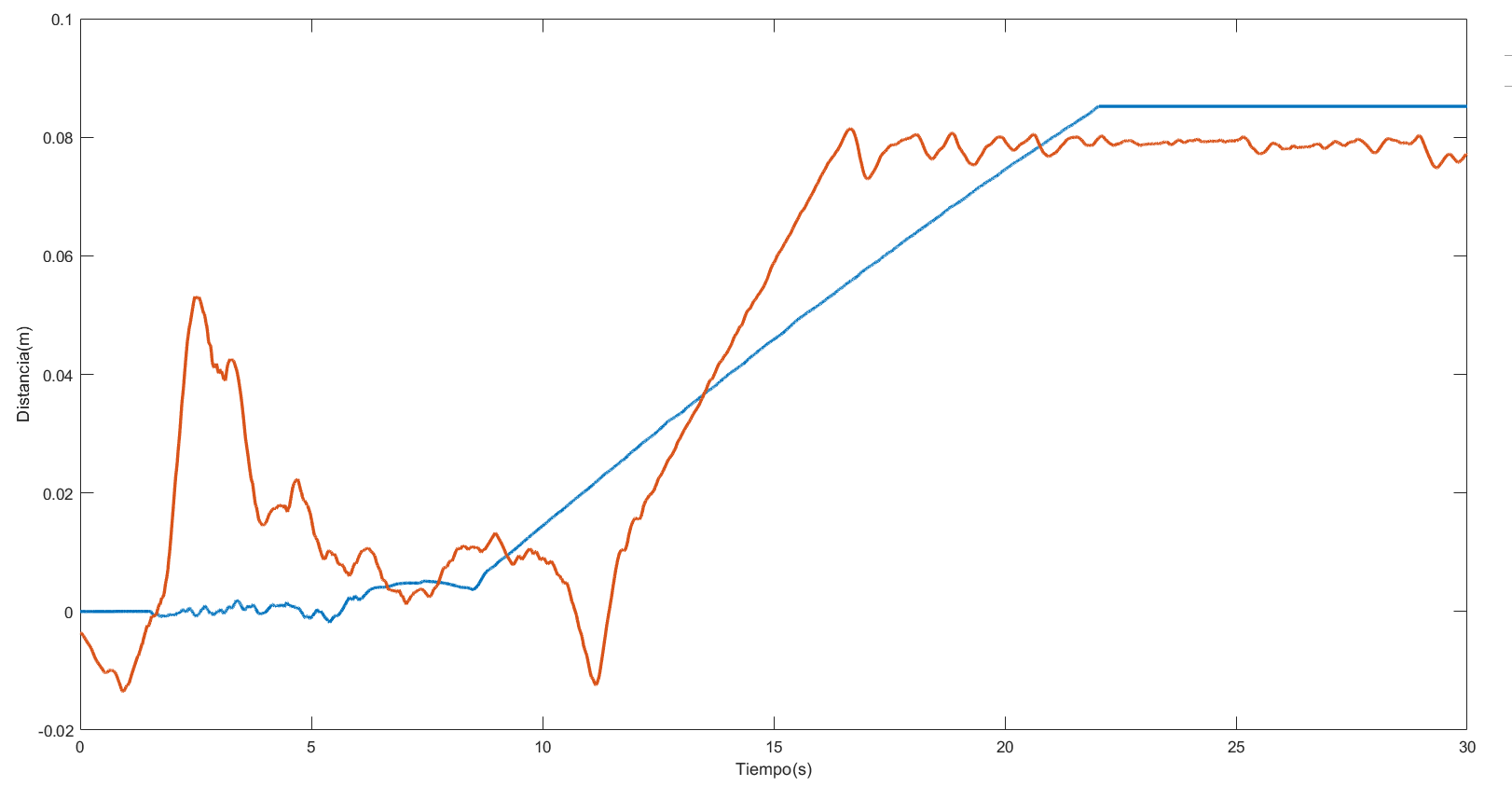

Supplement: Supplementary file 1 [file Data_Sheet_1.ZIP › figures/ComparisonCMY3.png]

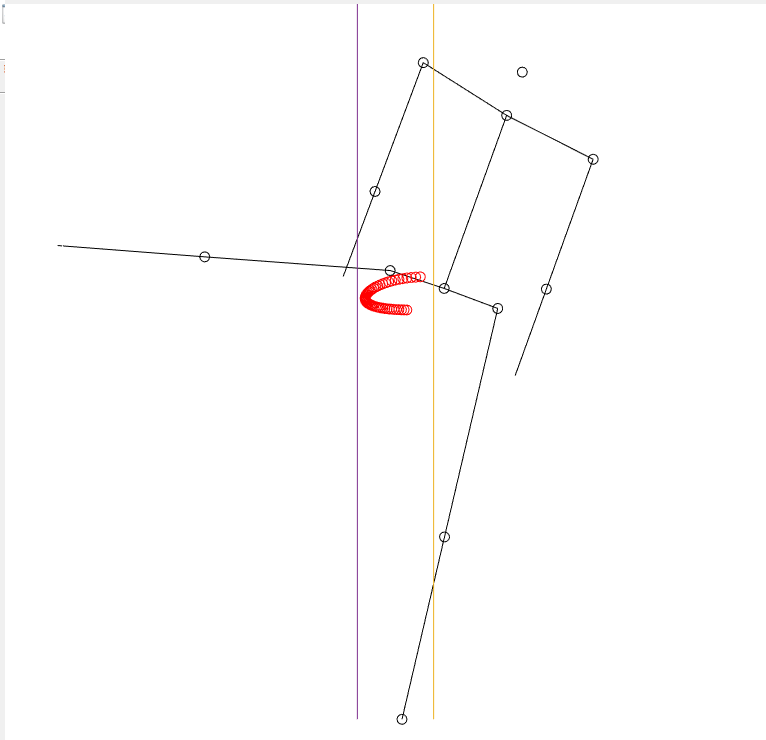

Supplement: Supplementary file 1 [file Data_Sheet_1.ZIP › figures/Approx4.png]

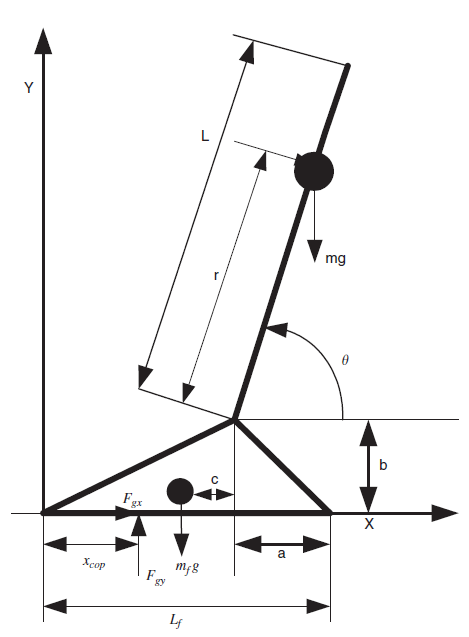

Supplement: Supplementary file 1 [file Data_Sheet_1.ZIP › figures/SimplifiedBipedalModel.png]

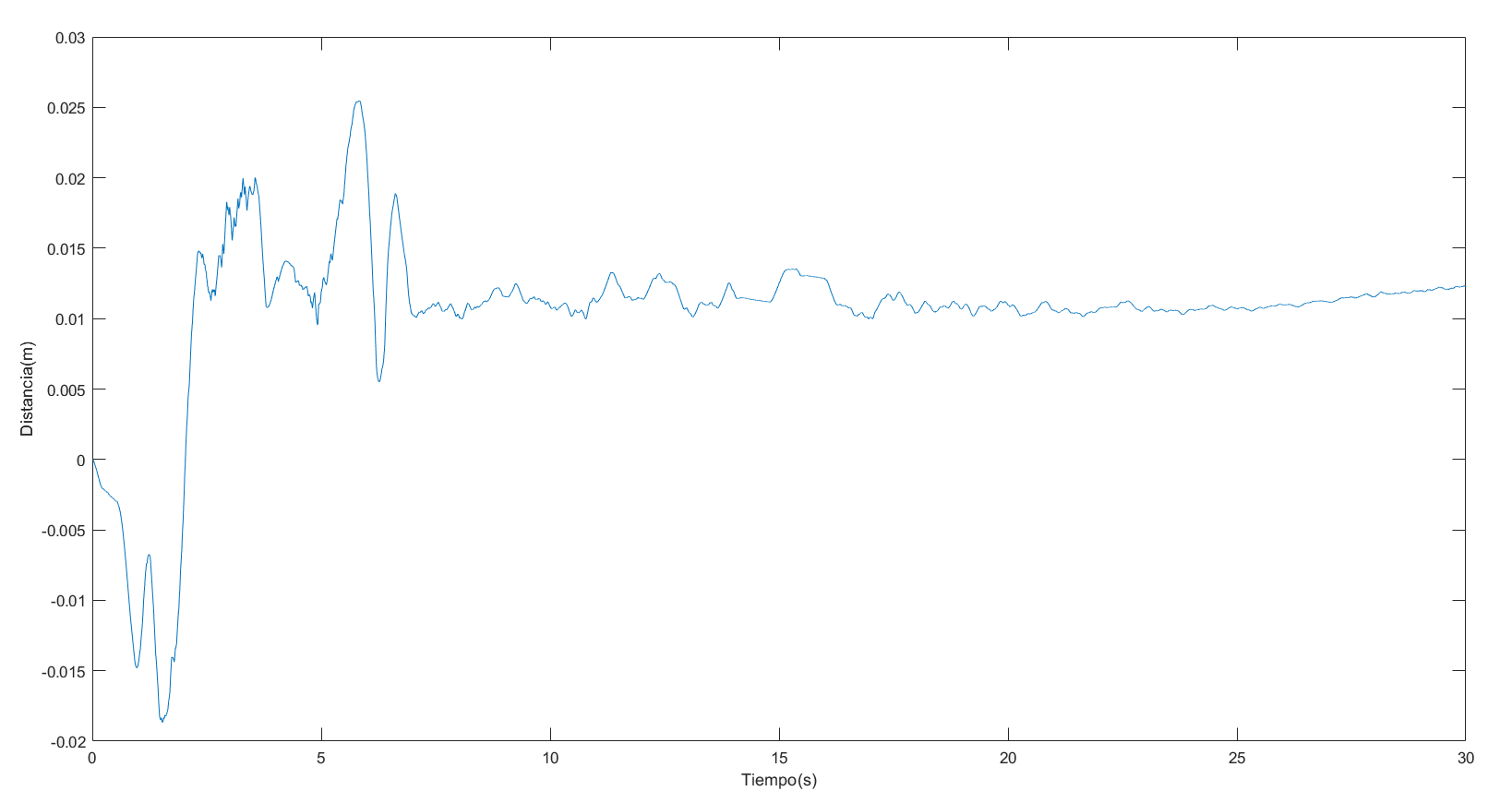

Supplement: Supplementary file 1 [file Data_Sheet_1.ZIP › figures/TrajectoryCMY.png]

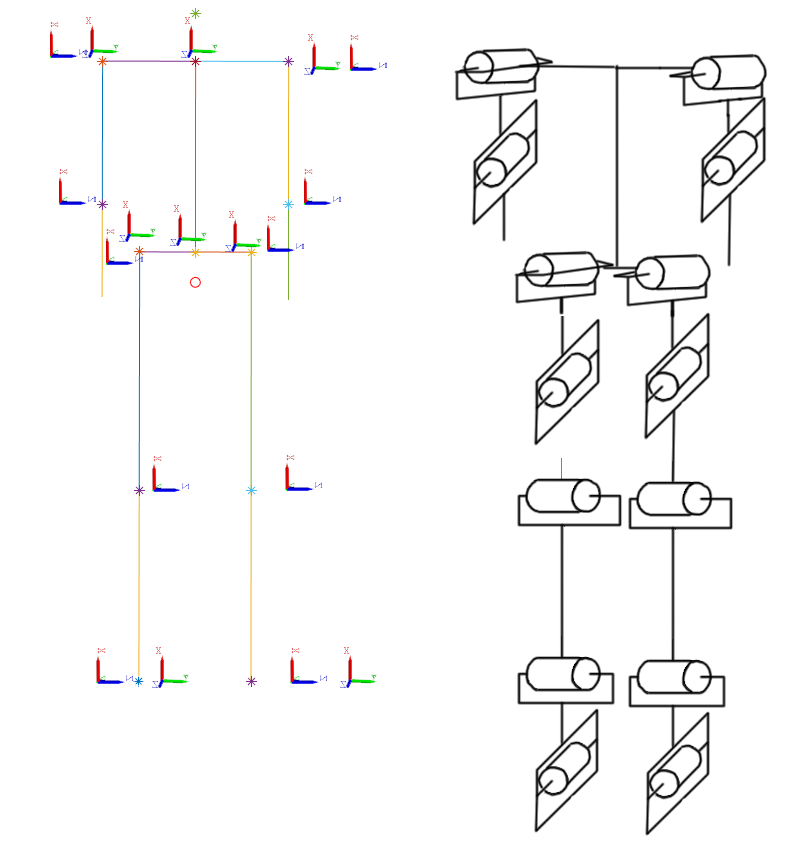

Supplement: Supplementary file 1 [file Data_Sheet_1.ZIP › figures/FullModel3.png]

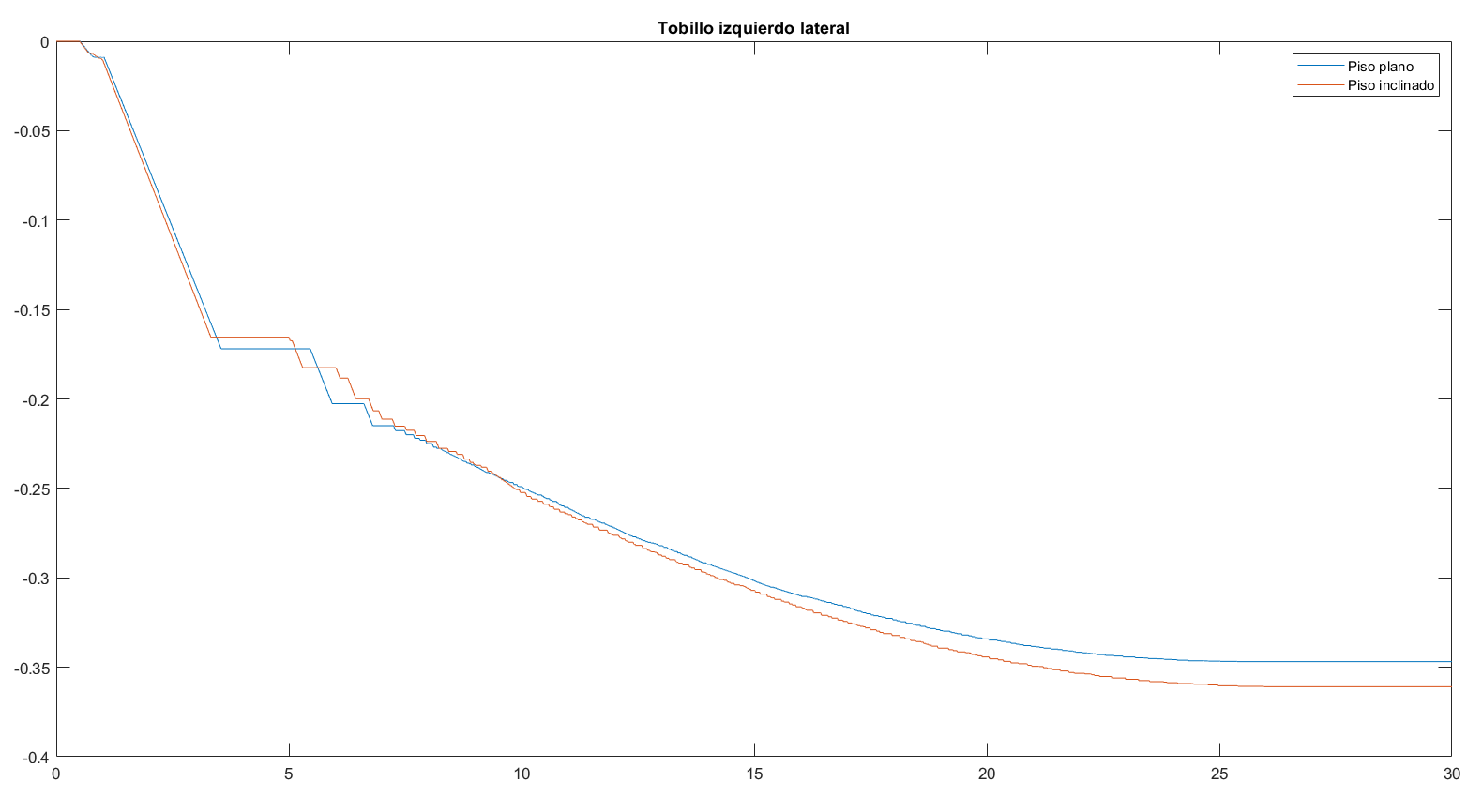

Supplement: Supplementary file 1 [file Data_Sheet_1.ZIP › figures/Comparison1Art3.png]

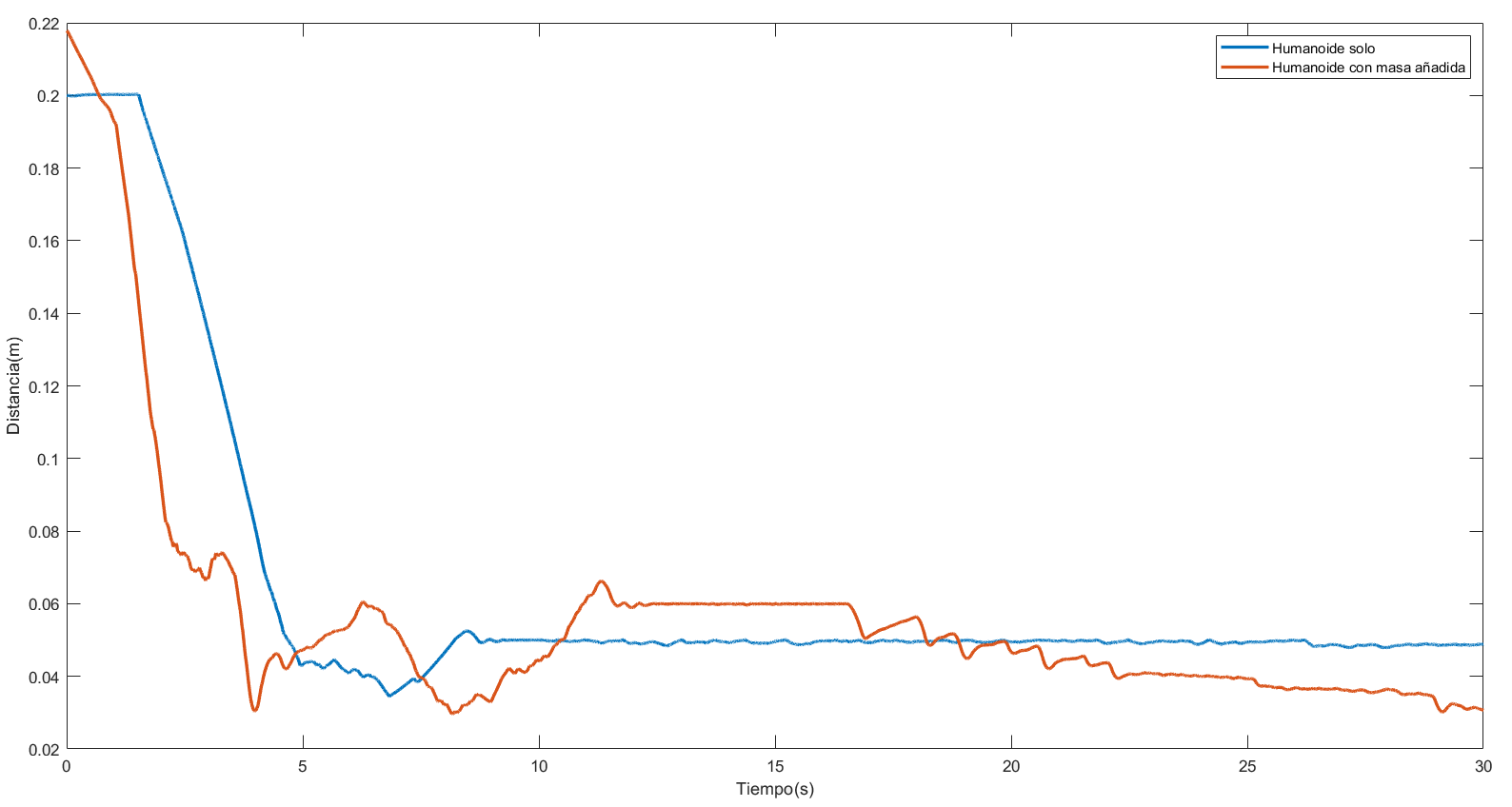

Supplement: Supplementary file 1 [file Data_Sheet_1.ZIP › figures/ComparisonCMX3.png]

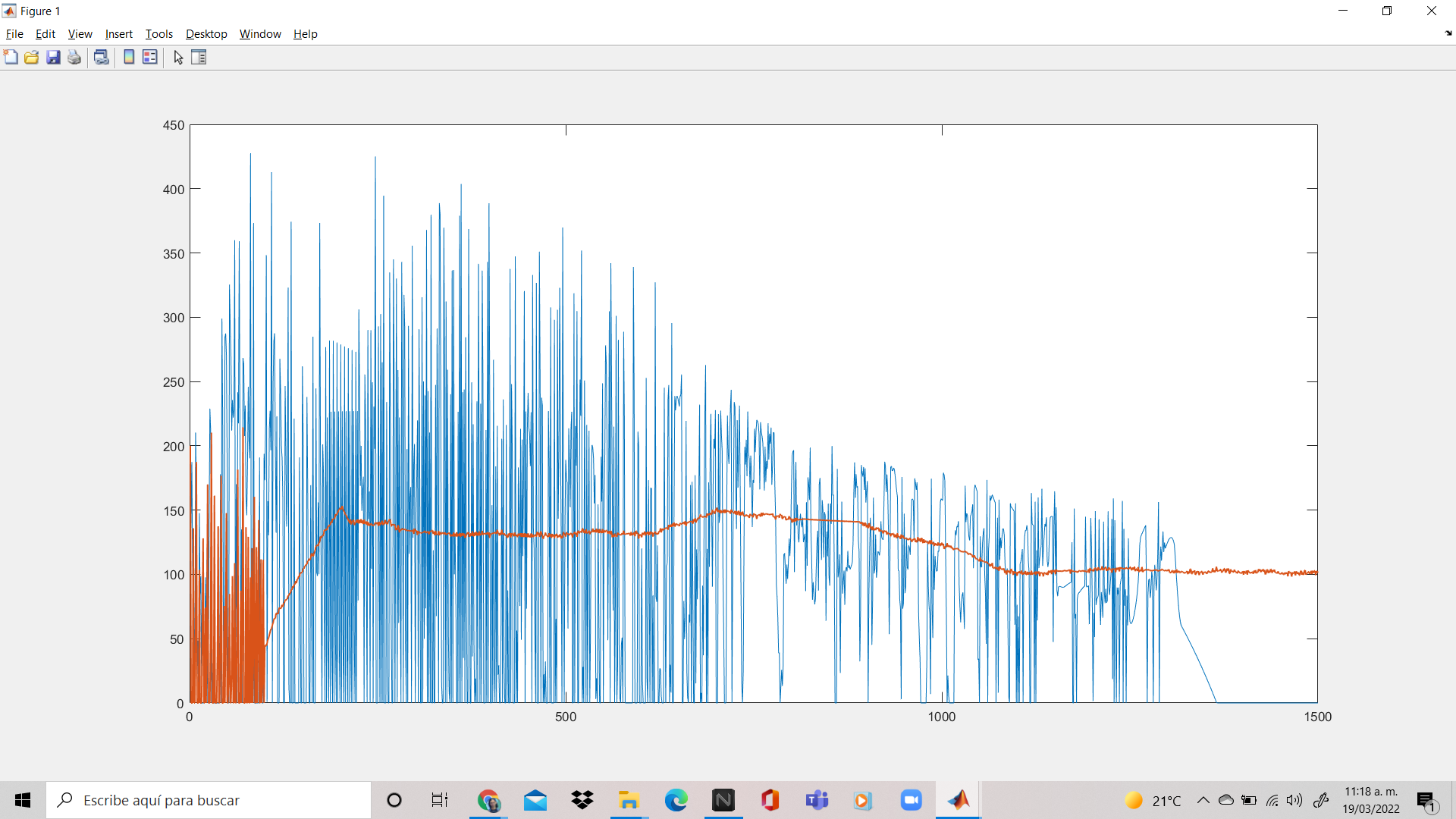

Supplement: Supplementary file 1 [file Data_Sheet_1.ZIP › figures/FilteredSignal.png]

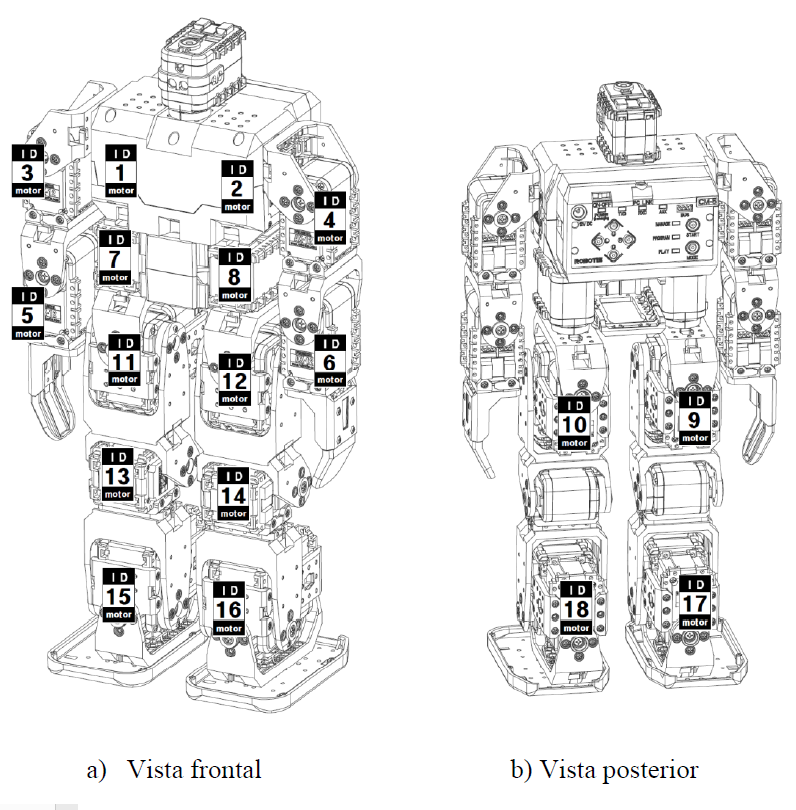

Supplement: Supplementary file 1 [file Data_Sheet_1.ZIP › figures/Bioloid.png]

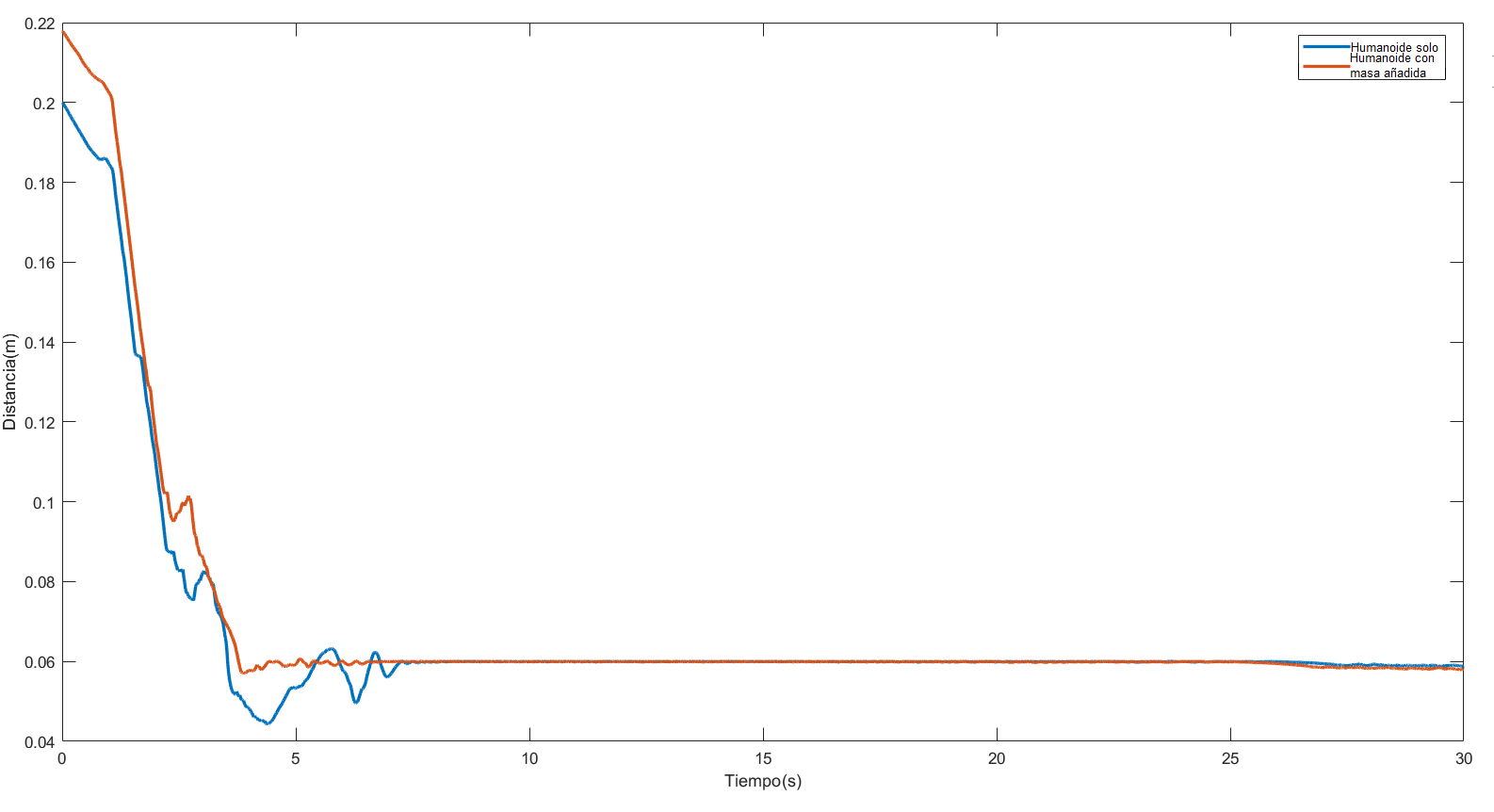

Supplement: Supplementary file 1 [file Data_Sheet_1.ZIP › figures/ComparisonCMX2.png]

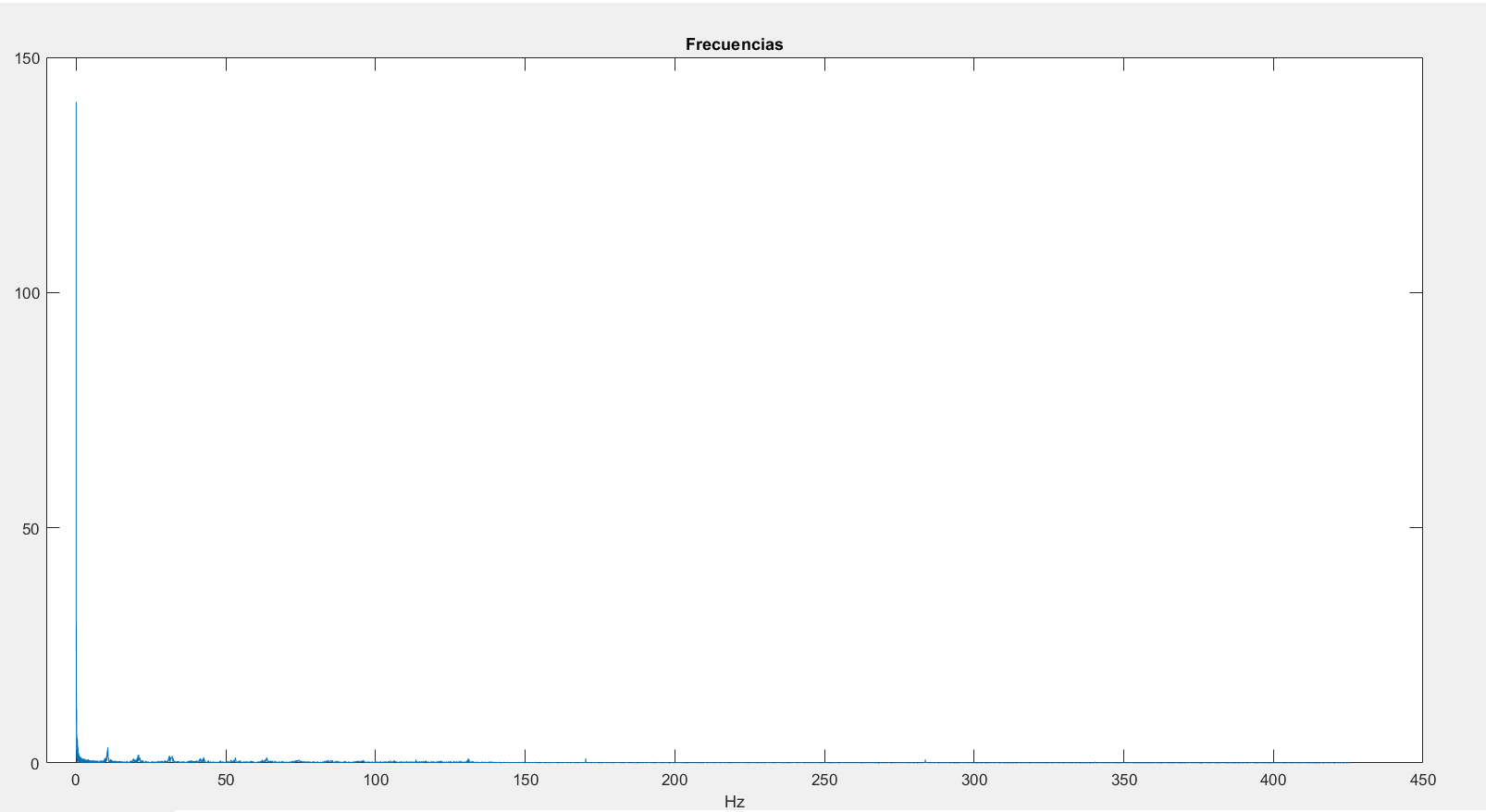

Supplement: Supplementary file 1 [file Data_Sheet_1.ZIP › figures/FrecuenciasSenal.png]

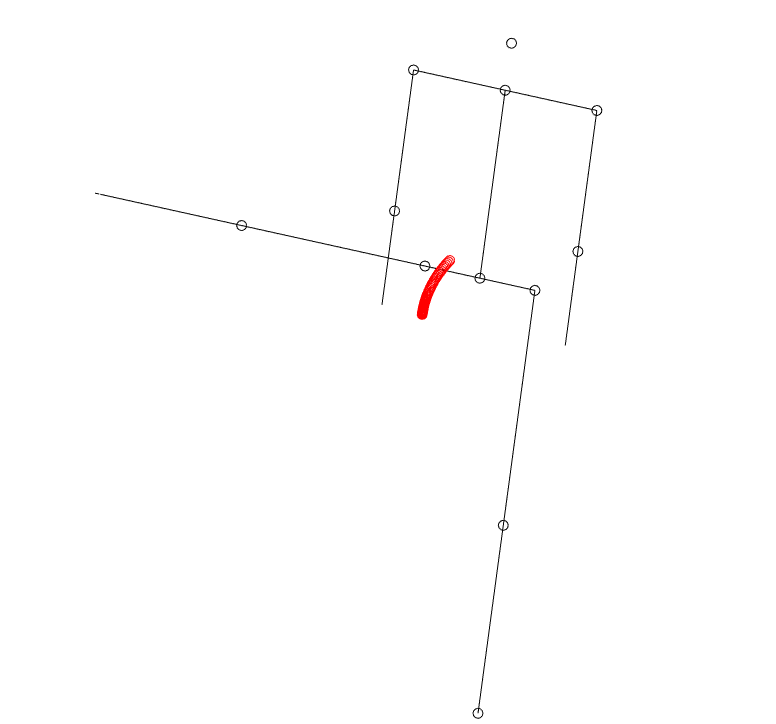

Supplement: Supplementary file 1 [file Data_Sheet_1.ZIP › figures/Approx1.png]

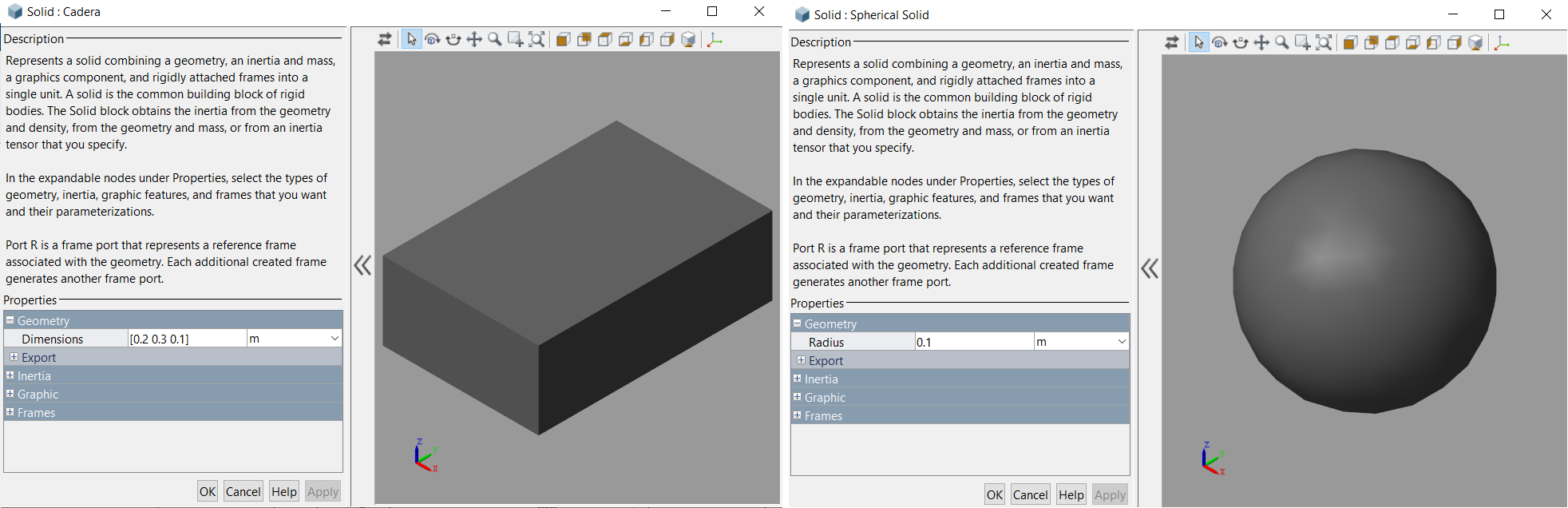

Supplement: Supplementary file 1 [file Data_Sheet_1.ZIP › figures/SimulinkHip.png]

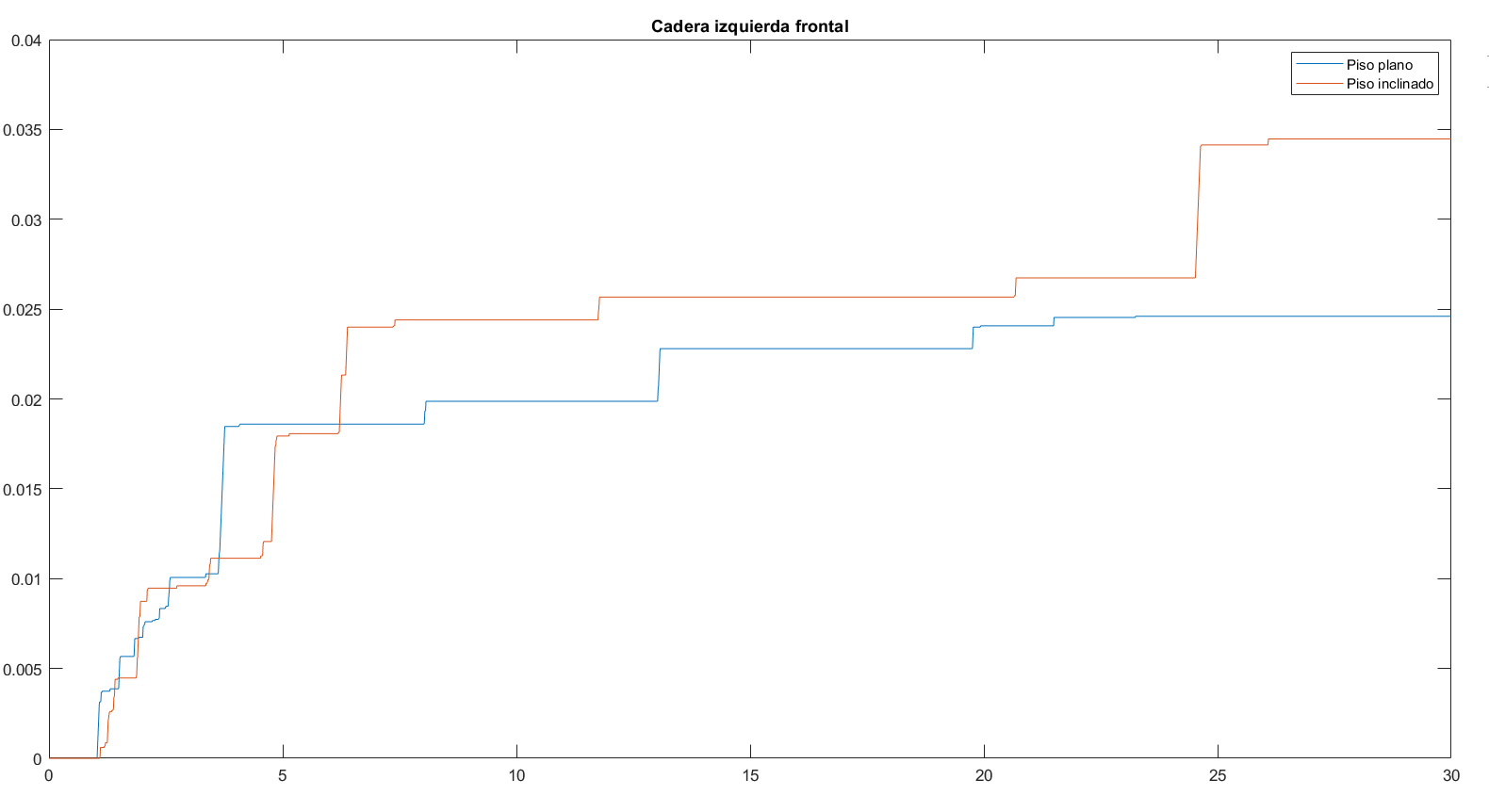

Supplement: Supplementary file 1 [file Data_Sheet_1.ZIP › figures/Comparison1Art2.png]

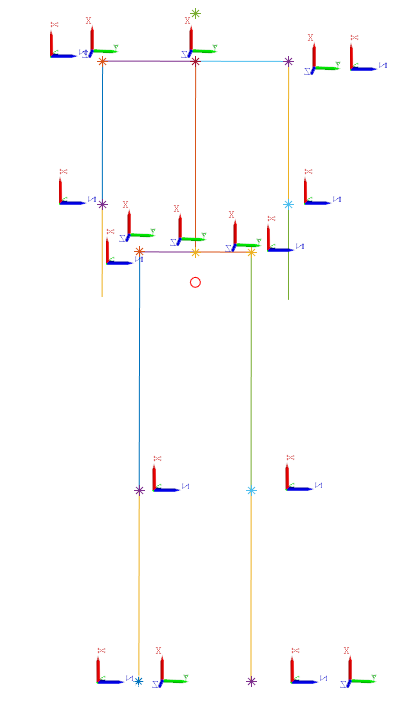

Supplement: Supplementary file 1 [file Data_Sheet_1.ZIP › figures/FullModel2.png]

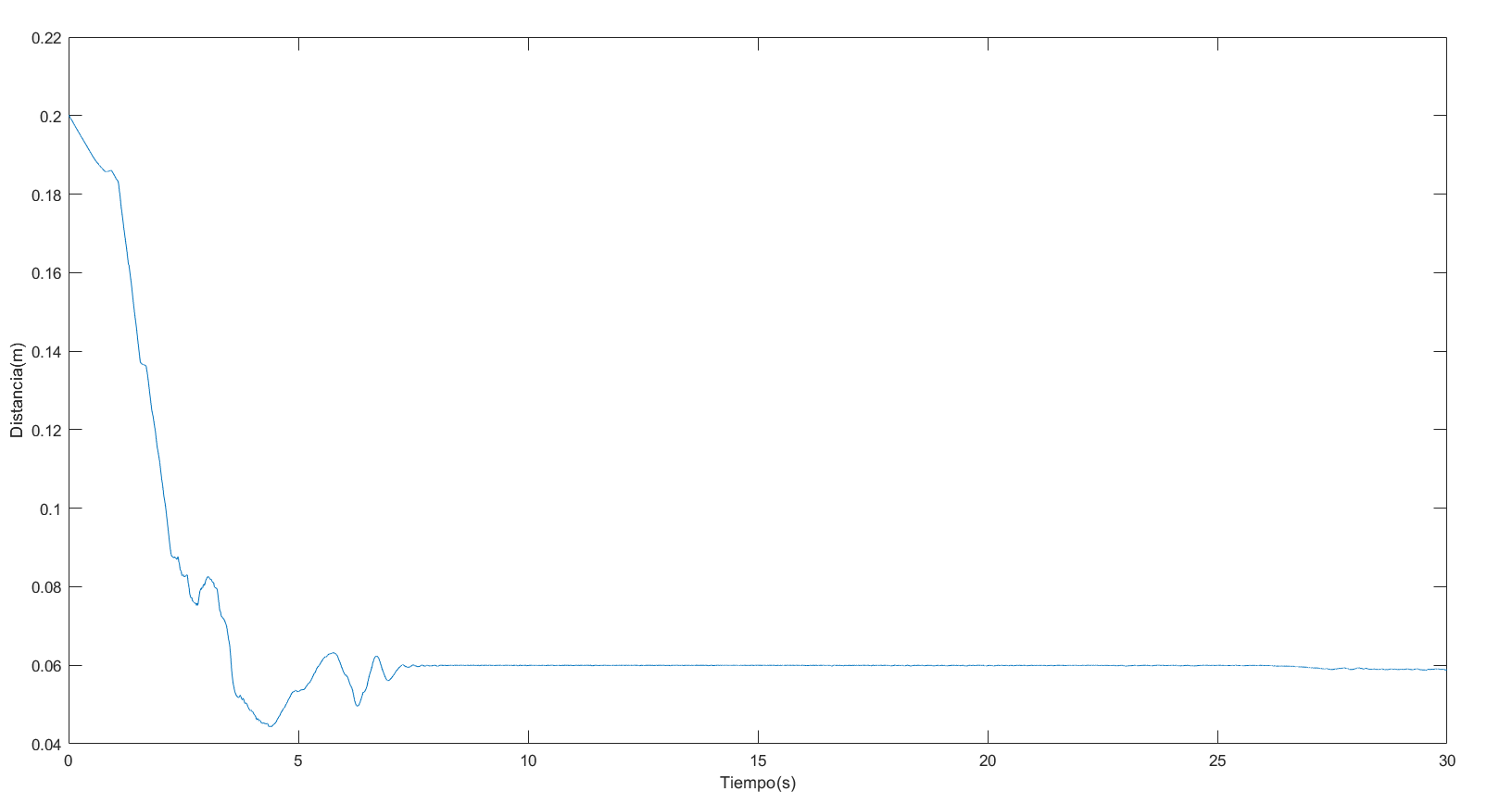

Supplement: Supplementary file 1 [file Data_Sheet_1.ZIP › figures/TrajectoryCMX.png]

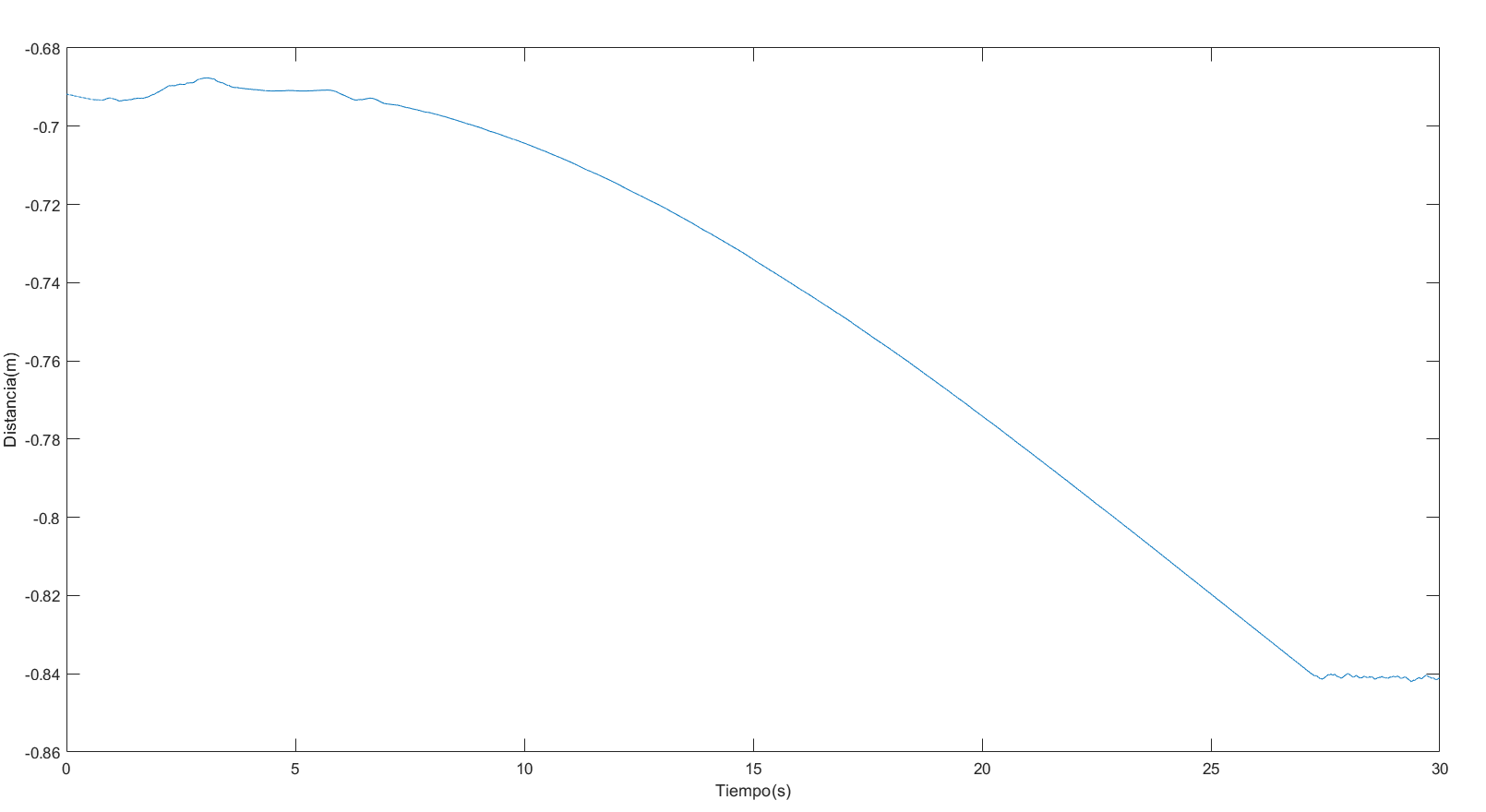

Supplement: Supplementary file 1 [file Data_Sheet_1.ZIP › figures/TrajectoryCMZ.png]

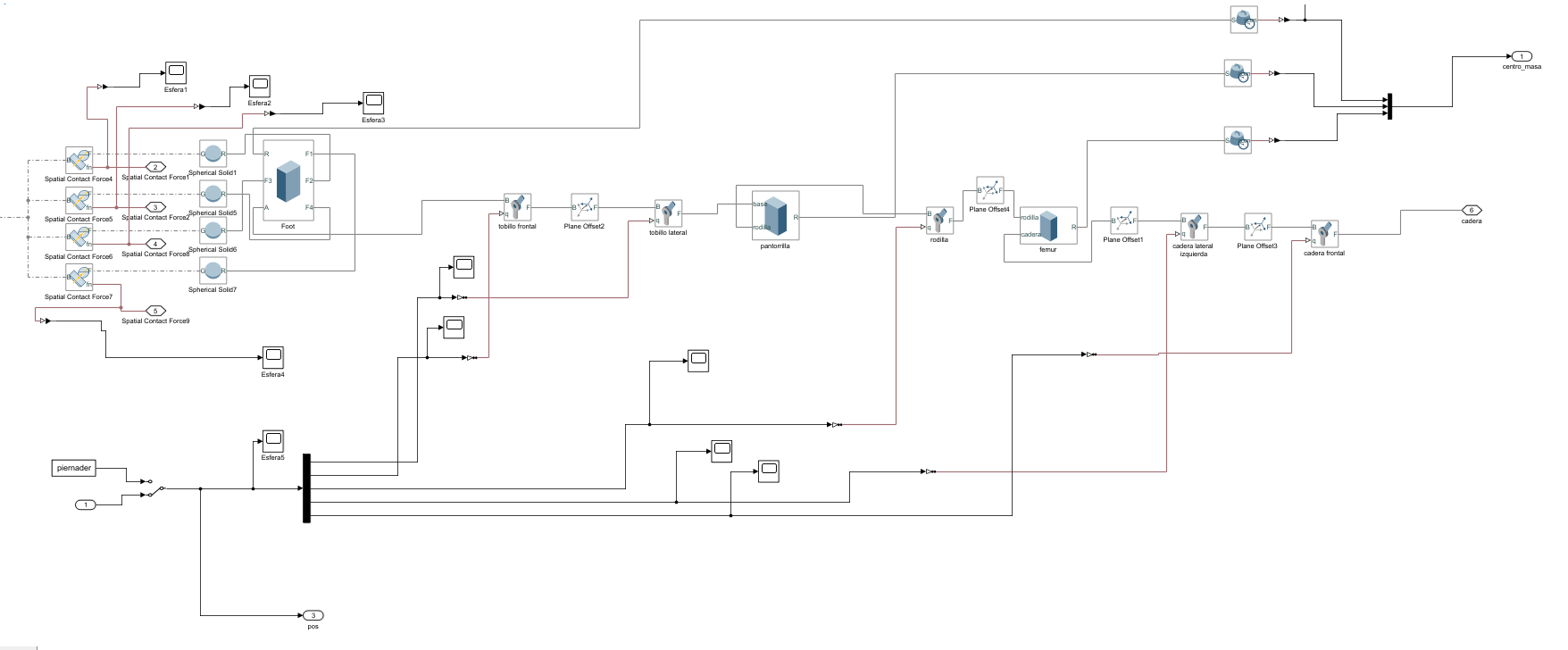

Supplement: Supplementary file 1 [file Data_Sheet_1.ZIP › figures/LegBlocks.png]

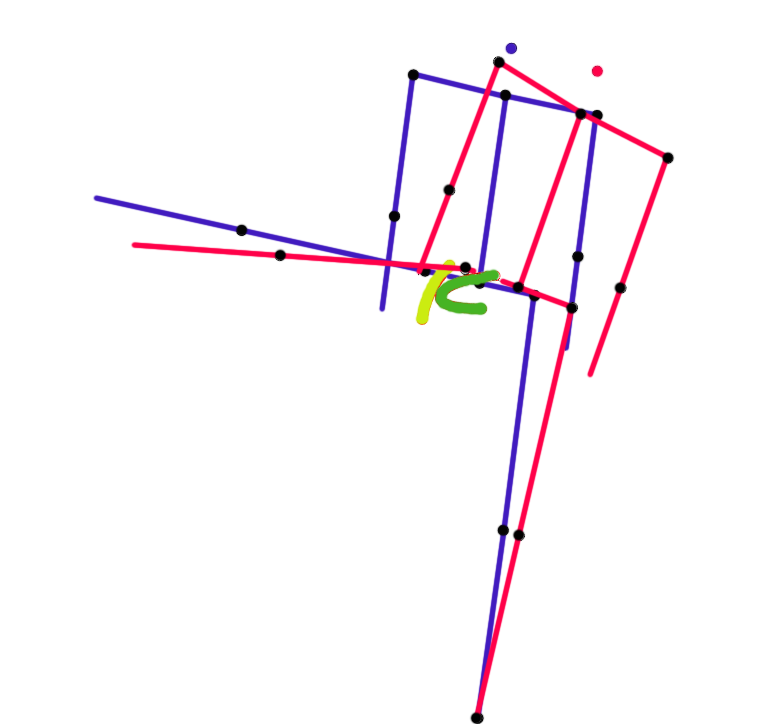

Supplement: Supplementary file 1 [file Data_Sheet_1.ZIP › figures/Approx3.png]

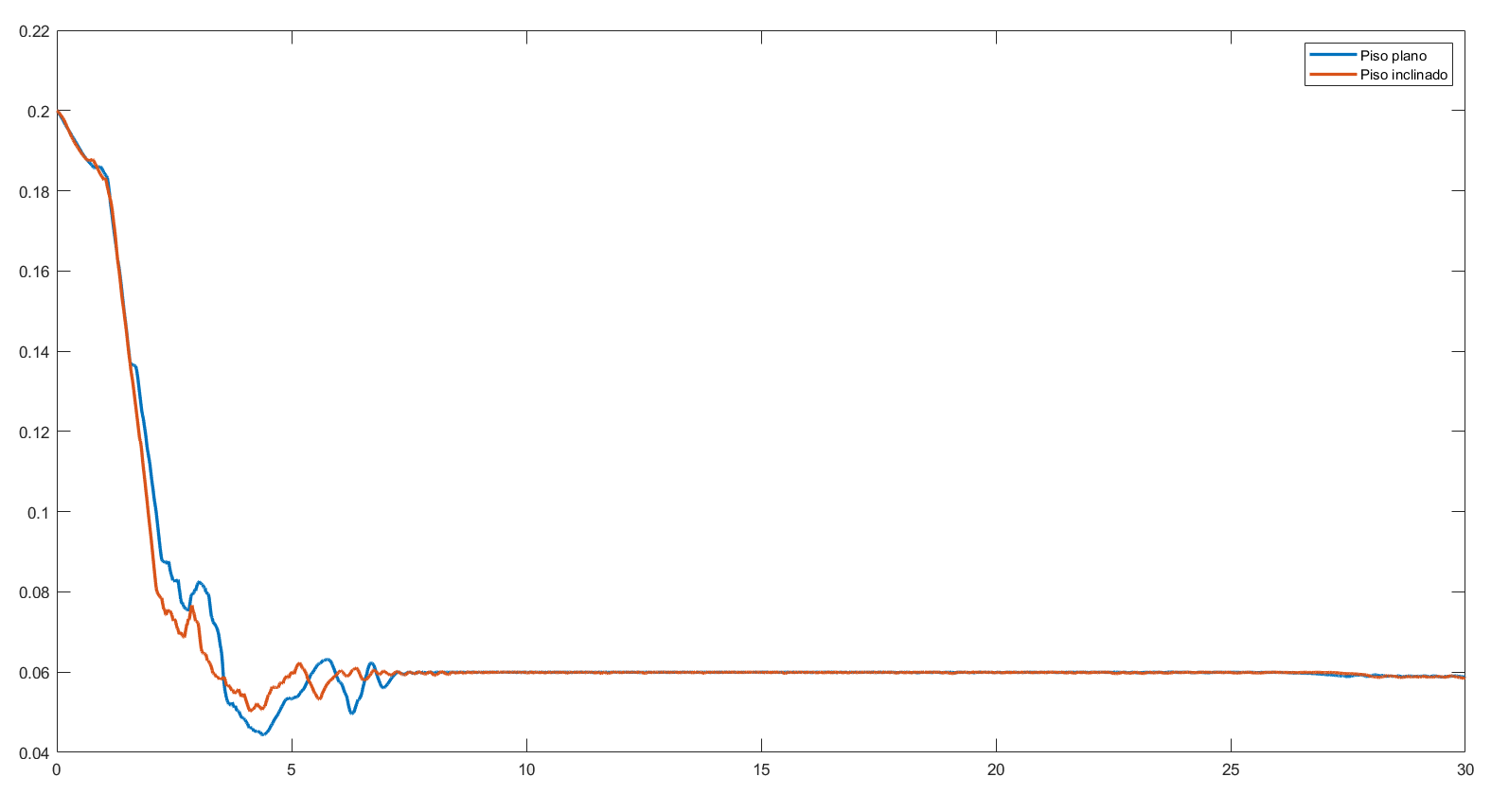

Supplement: Supplementary file 1 [file Data_Sheet_1.ZIP › figures/ComparisonCMX1.png]

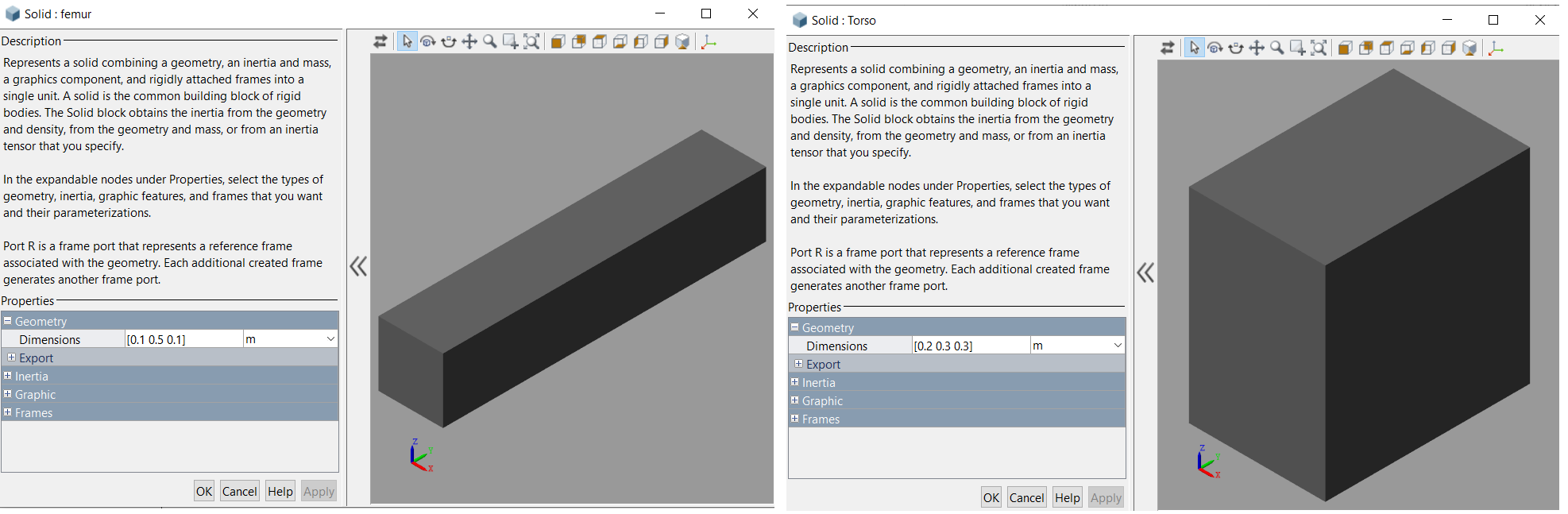

Supplement: Supplementary file 1 [file Data_Sheet_1.ZIP › figures/SimulinkFemur.png]

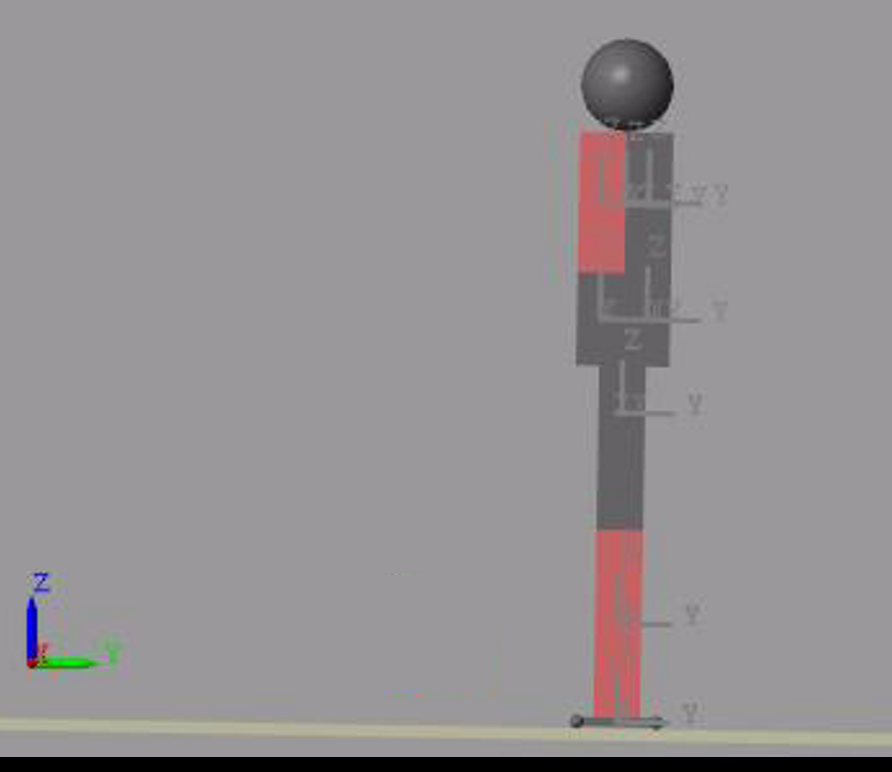

Supplement: Supplementary file 1 [file Data_Sheet_1.ZIP › figures/InclinedPosition1.png]

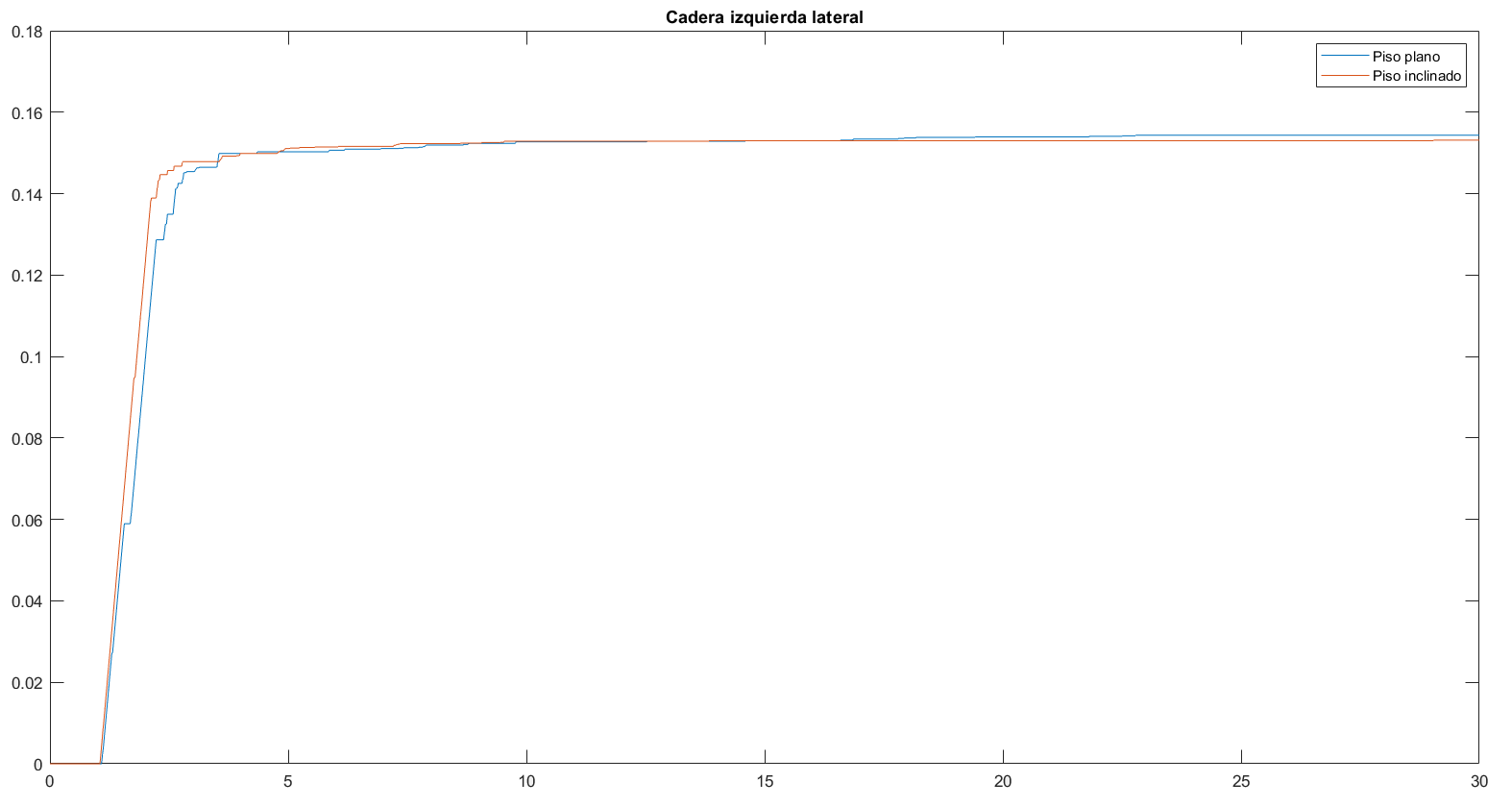

Supplement: Supplementary file 1 [file Data_Sheet_1.ZIP › figures/Comparison1Art1.png]
